# Supplementary material for: ND3 Cys39 in complex I is exposed during mitochondrial respiration
Source: Cell Chem Biol. Author manuscript; Available in PMC 2022 May 18. (PMC9076552; doi:10.1016/j.chembiol.2021.10.010)
Supplement: Article Plus Supplimentary information [file EMS144932-supplement-Article_Plus_Supplimentary_information.pdf]

Article

Cell Chemical Biology

ND3 Cys39 in complex I is exposed during mitochondrial respiration

Graphical abstract

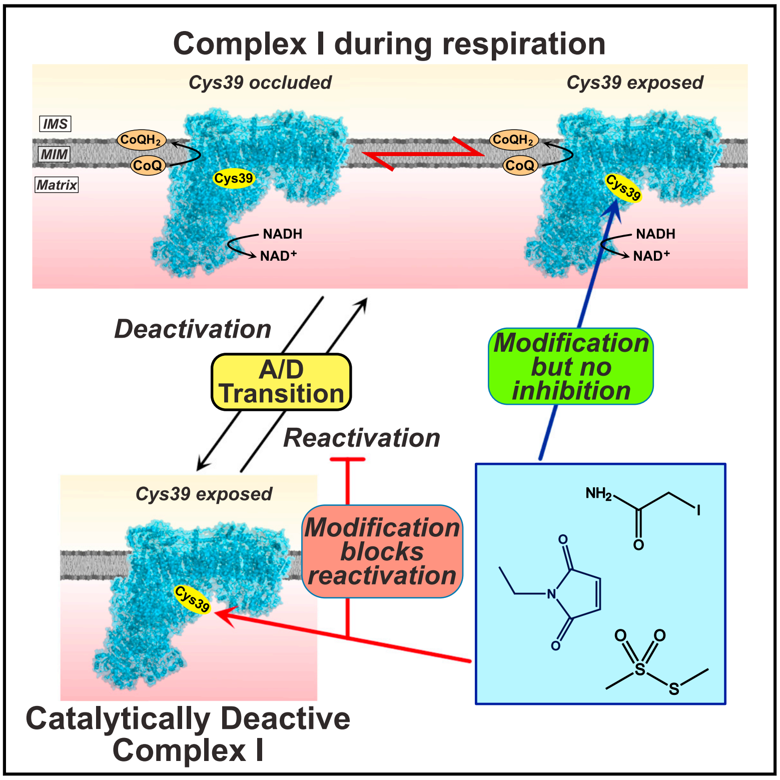

Highlights

- ND3-Cys39 in complex I is exposed during active mitochondrial respiration
- Modification of exposed Cys39 in active complex I does not impair respiration
- Modification of exposed Cys39 in deactive complex I prevents reactivation
- Complex I activity and Cys39 exposure are not linked directly

Authors

Nils Burger, Andrew M. James, John F. Mulvey, ..., Richard C. Hartley, Thomas Krieg, Michael P. Murphy

Correspondence

mpm@mrc-mbu.cam.ac.uk

In brief

The enigmatic active/deactive transition of complex I is linked to the exposure state of the critical ND3-Cys39 residue, functioning as critical indicator. Burger et al. show that Cys39 is exposed during complex I-linked respiration with important implications for the understanding of the active/deactive transition and complex I function.

Burger et al., 2022, Cell Chemical Biology *29*, 636–649
April 21, 2022 © 2021 The Author(s). Published by Elsevier Ltd.
https://doi.org/10.1016/j.chembiol.2021.10.010

CellPress

CellPress
OPEN ACCESS

Cell Chemical Biology

Article

ND3 Cys39 in complex I is exposed during mitochondrial respiration

Nils Burger,[1] Andrew M. James,[1] John F. Mulvey,[2] Kurt Hoogewijs,[1,3,4] Shujing Ding,[1] Ian M. Fearnley,[1] Marta Loureiro-López,[1] Abigail A.I. Norman,[5] Sabine Arndt,[1] Amin Mottahedin,[1,2,6] Olga Sauchanka,[2] Richard C. Hartley,[5] Thomas Krieg,[2] and Michael P. Murphy[1,7,*]
[1]Medical Research Council-Mitochondrial Biology Unit, University of Cambridge, Cambridge CB2 0XY, UK
[2]Department of Medicine, University of Cambridge, Addenbrooke's Hospital, Cambridge CB2 0QQ, UK
[3]The Wellcome Trust Centre for Mitochondrial Research, Institute for Cell and Molecular Biosciences, Newcastle University, Newcastle Upon Tyne NE2 4HH, UK
[4]Medical Research Council-Laboratory of Molecular Biology, Cambridge CB2 0QH, UK
[5]School of Chemistry, University of Glasgow, Glasgow G12 8QQ, UK
[6]Department of Physiology, Institute of Neuroscience and Physiology, Sahlgrenska Academy, University of Gothenburg, 405 30 Gothenburg, Sweden
[7]Lead contact
*Correspondence: mpm@mrc-mbu.cam.ac.uk
https://doi.org/10.1016/j.chembiol.2021.10.010

SUMMARY

Mammalian complex I can adopt catalytically active (A-) or deactive (D-) states. A defining feature of the reversible transition between these two defined states is thought to be exposure of the ND3 subunit Cys39 residue in the D-state and its occlusion in the A-state. As the catalytic A/D transition is important in health and disease, we set out to quantify it by measuring Cys39 exposure using isotopic labeling and mass spectrometry, in parallel with complex I NADH/CoQ oxidoreductase activity. To our surprise, we found significant Cys39 exposure during NADH/CoQ oxidoreductase activity. Furthermore, this activity was unaffected if Cys39 alkylation occurred during complex I-linked respiration. In contrast, alkylation of catalytically inactive complex I irreversibly blocked the reactivation of NADH/CoQ oxidoreductase activity by NADH. Thus, Cys39 of ND3 is exposed in complex I during mitochondrial respiration, with significant implications for our understanding of the A/D transition and the mechanism of complex I.

INTRODUCTION

Mammalian complex I can reversibly adopt catalytically active (A-) and catalytically deactive (D-) states. The ability of mammalian complex I to form these distinct states was foreshadowed in 1950, when a gradual loss in NADH oxidase activity was noted after incubation at 37°C (Slater, 1950) that was prevented by NADH (Luzikov et al., 1970; Rossi et al., 1965). These observations were rationalized by demonstrating the transition between these states in submitochondrial particles (Kotlyar and Vinogradov, 1990). This confirmed that complex I undergoes time-dependent conversion to a catalytically inactive D-state, which can revert to the catalytically active A-state in the presence of NADH to initiate electron transport through the complex to CoQ (Kotlyar and Vinogradov, 1990; Vinogradov, 1998). Complex I inhibition by electrophiles, e.g., *N*-ethyl maleimide (NEM), was subsequently ascribed to modification of the ND3 subunit Cys39 residue, which appeared to be selectively exposed in the catalytically inactive D-state (Galkin et al., 2008). This led to a model of the catalytic A/D transition, in which Cys39 was occluded in the catalytic A-state, rendering complex I resistant to thiol reagents, but exposed to reaction with electrophiles in the D-state, that prevented reactivation of catalytic turnover by NADH (Figure 1).

Structural insights into the catalytic A/D transition emerged from electron cryomicroscopy (CryoEM) analysis of mammalian complex I, which identified two distinct conformations (Agip et al., 2018; Blaza et al., 2018; Fiedorczuk et al., 2016; Zhu et al., 2016). In the CryoEM structure prepared from complex I, Cys39 was found either occluded, or it was not resolved and could not be modeled, presumably because the loop containing Cys39 was mobile and solvent exposed. Together, these catalytic and structural studies can be rationalized by a catalytically active A-state in which Cys39 is fully occluded and a catalytically inactive D-state in which it is fully exposed (Figure 1). However, complex I is a dynamic enzyme and its conformation during catalytic turnover is unlikely to correspond fully to a single, resting structure. Consequently, the correspondence between the CryoEM structure states during NADH/coenzyme Q (CoQ) oxidoreductase activity is unclear. To emphasize this potentially important difference, here we use the terms catalytic A/D-states, and structural A/D-states. Importantly, the structural A-state is a resting state, whereas the catalytic A-state in which the enzyme is actively turning over is likely to encompass several conformations.

This is an open access article under the CC BY license (http://creativecommons.org/licenses/by/4.0/).

CellPress
OPEN ACCESS

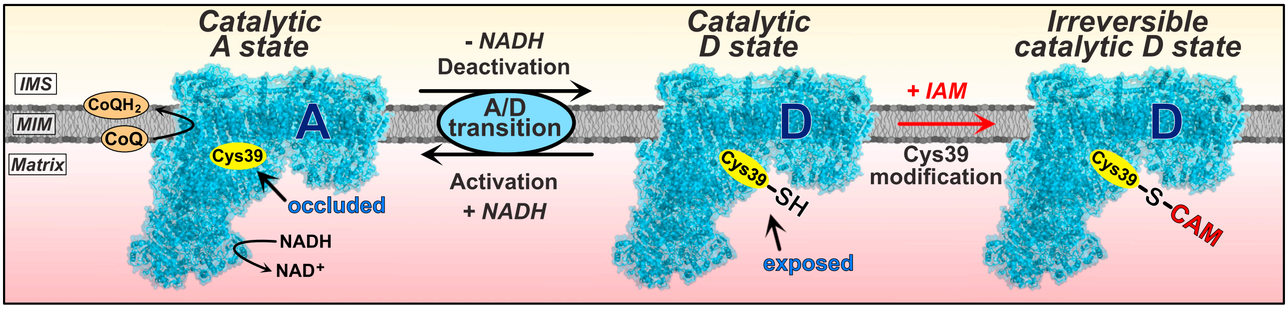

Figure 1. The catalytic active/deactive transition of complex I – the current model

In the presence of its substrate NADH, under conditions that can initiate electron movement through complex I to CoQ, the enzyme adopts a catalytically competent conformation in which ND3 Cys39 is thought to be fully occluded. This state may correspond to the structural A-state of the complex. When complex I is not turning over, in the absence of NADH or in absence of electron flux during ischemia, complex I reversibly transitions into a catalytically inactive D-state that exposes Cys39. This state may correspond to the structural D-state of the complex. Catalytically deactive complex I can reinitiate catalysis in the presence of NADH-driven electron movement through the enzyme. The exposed Cys39 residue of the catalytically deactive enzyme can be irreversibly modified by alkylating agents such as iodoacetamide (IAM; carbamidomethylation [CAM] of Cys39), which permanently locks complex I in a catalytically inactive state.

The catalytic A/D transition occurs in vertebrates and in fungi containing complex I (*Neurospora crassa* and *Yarrowia lipolytica*) (Agip et al., 2018; Blaza et al., 2018; Fiedorczuk et al., 2016; Gorenkova et al., 2013; Grba and Hirst, 2020; Grivennikova et al., 2003; Kalashnikov et al., 2011; Maklashina et al., 2002, 2003, 2004; Siebels and Dröse, 2016; Zhu et al., 2016). In contrast, complex I of invertebrate metazoans as well as bacteria (*Paracoccus denitrificans, Thermus thermophilus*) does not undergo a catalytic A/D transition (Jarman et al., 2021; Kotlyar et al., 1998; Maklashina et al., 2003), although it has been claimed to occur for the *Escherichia coli* enzyme (Belevich and Verkhovskaya, 2016; Belevich et al., 2017a, 2017b). The physiological role for the catalytic A/D transition has been proposed as a mechanism to fine-tune catalytic activity in response to oxygen concentration (Babot et al., 2014; Galkin and Moncada, 2017). In addition, the Na^+/H^+ antiporter activity of the catalytic D-state was suggested to contribute to mitochondrial ion transfer (Roberts and Hirst, 2012). Slow reactivation of complex I upon reperfusion following ischemia could limit excessive reactive oxygen species (ROS) production and oxidative damage (Babot et al., 2014; Galkin, 2019). The exposure of Cys39 might act as a regulatory switch for the modulation of the catalytic A/D transition under physiological conditions, or to enable assembly/degradation of complex I without NADH consumption or ROS production (Babot et al., 2014; Chouchani et al., 2016; Dröse et al., 2016; Galkin and Moncada, 2017; Gorenkova et al., 2013; Kahl et al., 2018) (Figure 1).

The catalytic A/D transition has emerged as a pharmacological target to modulate complex I activity. For example, *S*-nitrosation of Cys39 by NO donors temporarily locks complex I in the catalytic D-state (Clementi et al., 1998; Galkin and Moncada, 2007) and a mitochondria-targeted NO donor MitoSNO (Mito-*S*-nitroso-*N*-acetylpenicillamine) (Prime et al., 2009) that selectively *S*-nitrosated Cys39 *in vivo*, decreased ischemia-reperfusion (IR) injury (Chouchani et al., 2013; Kim et al., 2018; Methner et al., 2014; Wilson et al., 2018).

Consequently, there is considerable interest in fully characterizing ND3 Cys39 exposure during the catalytic A/D transition *in vivo* to understand its physiological function and to explore its potential as a drug target. Previous assessments of Cys39 exposure during the catalytic A/D transition were qualitative or semi-quantitative, and consequently the correspondence between Cys39 exposure and complex I NADH/CoQ oxidoreductase activity was unclear (Chouchani et al., 2013; Galkin et al., 2008; Gavrikova and Vinogradov, 1999; Gorenkova et al., 2013; Hernansanz-Agustín et al., 2017). Here we have quantified Cys39 exposure during the catalytic A/D transition by isotopic chemical labeling and mass spectrometry in parallel with complex I NADH/CoQ oxidoreductase activity in a range of systems. To our surprise, we found that while complex I was respiring a significant proportion of Cys39 in ND3 was exposed to reaction with thiol alkylating agents. Our work also demonstrates that alkylation of exposed Cys39 in the catalytic D-state permanently locks complex I in a catalytically inactive state. However, when complex I is alkylated during respiration, the NADH/CoQ oxidoreductase activity is retained, despite the exposure and alkylation of Cys39. These findings have important implications for our understanding of complex I function.

RESULTS

Assessing complex I catalytic A/D transition by NADH/CoQ oxidoreductase activity

To assess the catalytic A/D transition, we used bovine heart mitochondrial membranes (BHMMs), which have an accessible complex I NADH binding site, enabling us to quantify the catalytic A- and D-states from the rotenone-sensitive NADH/dQ (decylubiquinone) oxidoreductase activity. To induce the catalytic D-state, we incubated samples at 37°C without NADH. The catalytic A-state was generated by reactivating a portion of the deactivated sample with NADH on ice (Figure 2A). Complex I in the catalytic A-state rapidly reached its maximum NADH/dQ oxidoreductase activity, while the catalytic D-state was initially inactive, but reactivated over 8 to 10 min with NADH (Figure 2B).

Next, we assessed the effect of the thiol alkylating reagent iodoacetamide (IAM) (Figure S1A) on NADH/dQ oxidoreductase activity. Incubating the catalytic D-state with IAM prevented its reactivation with NADH, but did not affect the activity of the catalytic A-state (Figure 2C). A dose-response showed that [IAM] ≥ 20 mM prevented reactivation of the catalytic D-state (Figure 2D).

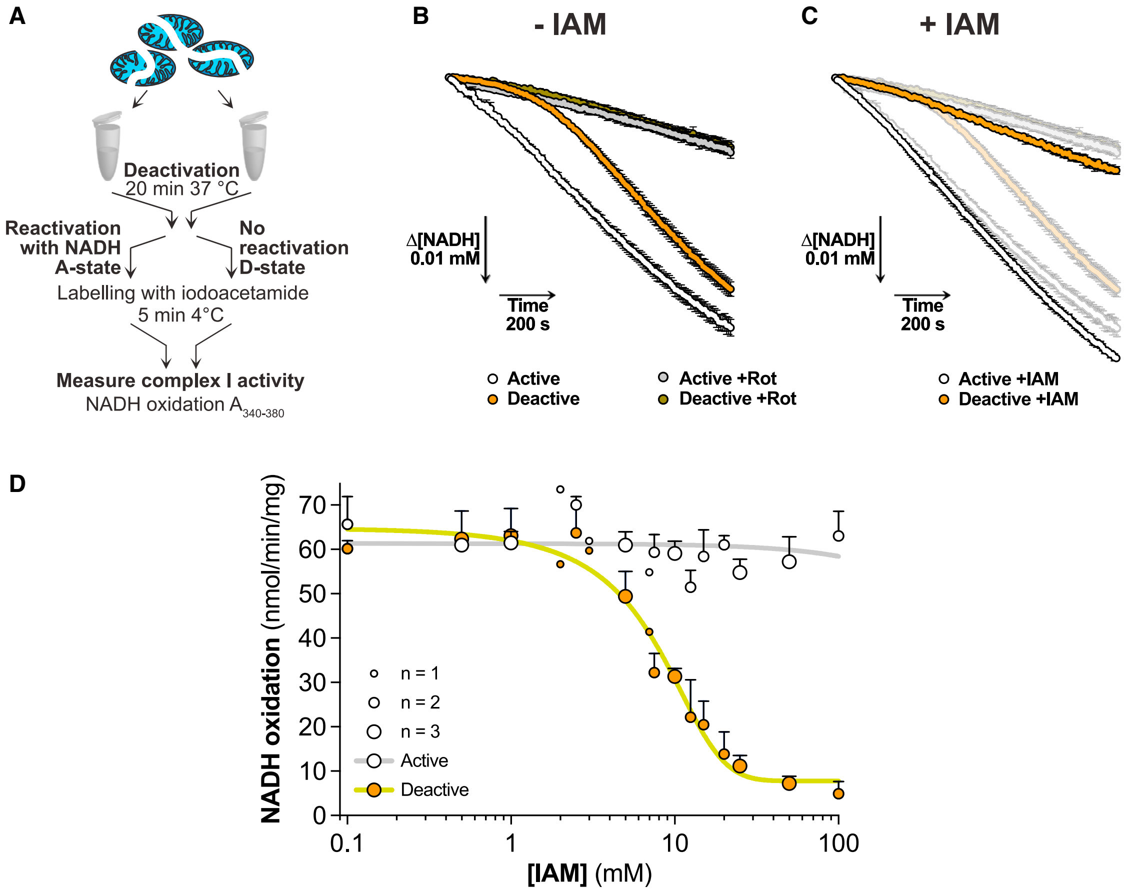

Figure 2. Iodoacetamide (IAM) selectively inhibits NADH/CoQ oxidoreductase activity of catalytically deactive complex I

(A) Schematic of the preparation of catalytically active and deactive complex I in BHMMs with subsequent iodoacetamide labeling for activity assessment.

(B) Representative traces of NADH oxidation by catalytically active and deactive BHMMs ± rotenone. Mean ± SEM of three wells. Under these conditions the maximal rate of NADH consumption was ~82% sensitive to rotenone. The length of the arrows indicates the scale.

(C) Representative traces of NADH oxidation by catalytically active and deactive BHMMs labeled with 100 mM of IAM. Mean ± SEM of three wells. A shadow of Figure 2B is underlaid for comparison. The length of the arrows indicates the scale.

(D) NADH/dQ oxidoreductase activity in catalytically active and deactive BHMMs upon labeling with increasing concentrations of IAM for 5 min on ice. The number of replicate experiments is indicated by the dot size. Data are presented as mean ± range (n = 2) or mean ± SEM (n = 3). Each experiment represents the average value of three wells.

Other thiol reagents, *S*-methyl methanethiosulfonate (MMTS) and NEM (Figure S1A), and TPP-IAM, a triphenylphosphonium (TPP)-tagged IAM (Figure S1B), also prevented D-state reactivation without affecting the catalytic A-state (Figures S1C–S1E). Therefore, the NADH/dQ oxidoreductase activity of the complex I catalytic A-state is unaffected by thiol reagents, while treatment of the catalytic D-state with these reagents prevents complex I reactivation.

ND3 Cys39 is exposed in catalytically active complex I

We quantified Cys39 exposure on activated or deactivated complex I in BHMMs by first labeling exposed Cys39 with light (L-) IAM, followed by denaturation and chemical reduction to expose occluded Cys39 for labeling with isotopically heavy ($^{13}C_2$, 2-d_2) (H-)IAM (note, the sequence of light and heavy labeling can be interchanged) (Figure 3A). Following trypsin cleavage, the H- and L-labeled peptides were quantified by mass spectrometry, either by untargeted proteomics (UTP), or by targeted multiple reaction monitoring (MRM) (method details: Labeling of Cys39 during respiration, Figure S2). Unexpectedly, UTP analysis of BHMMs treated to generate the catalytic A- and D-states of complex I (Figure 2A) showed that ~65% of Cys39 was exposed in the catalytic A-state, compared with ~93% in the catalytic D-state (Figure 3B). UTP analysis using H- and L-NEM or TPP-IAM found similar levels of Cys39 exposure in the catalytic A-state (Figure 3C). To more directly relate complex I NADH/dQ oxidoreductase activity and Cys39 exposure, we combined their assessment in a single sequential experiment (Figure 3D). Again, this showed significant Cys39 alkylation in the catalytic A-state that did not affect NADH/dQ oxidoreductase activity (Figure 3E). We carried out extensive technical controls, to assess the effectiveness of the Cys39 alkylation (method details: Labeling of Cys39 during respiration, Figure S3). Therefore, we conclude that when complex I is in the catalytic A-state, Cys39 is largely exposed and can be alkylated without altering its NADH/dQ oxidoreductase activity.

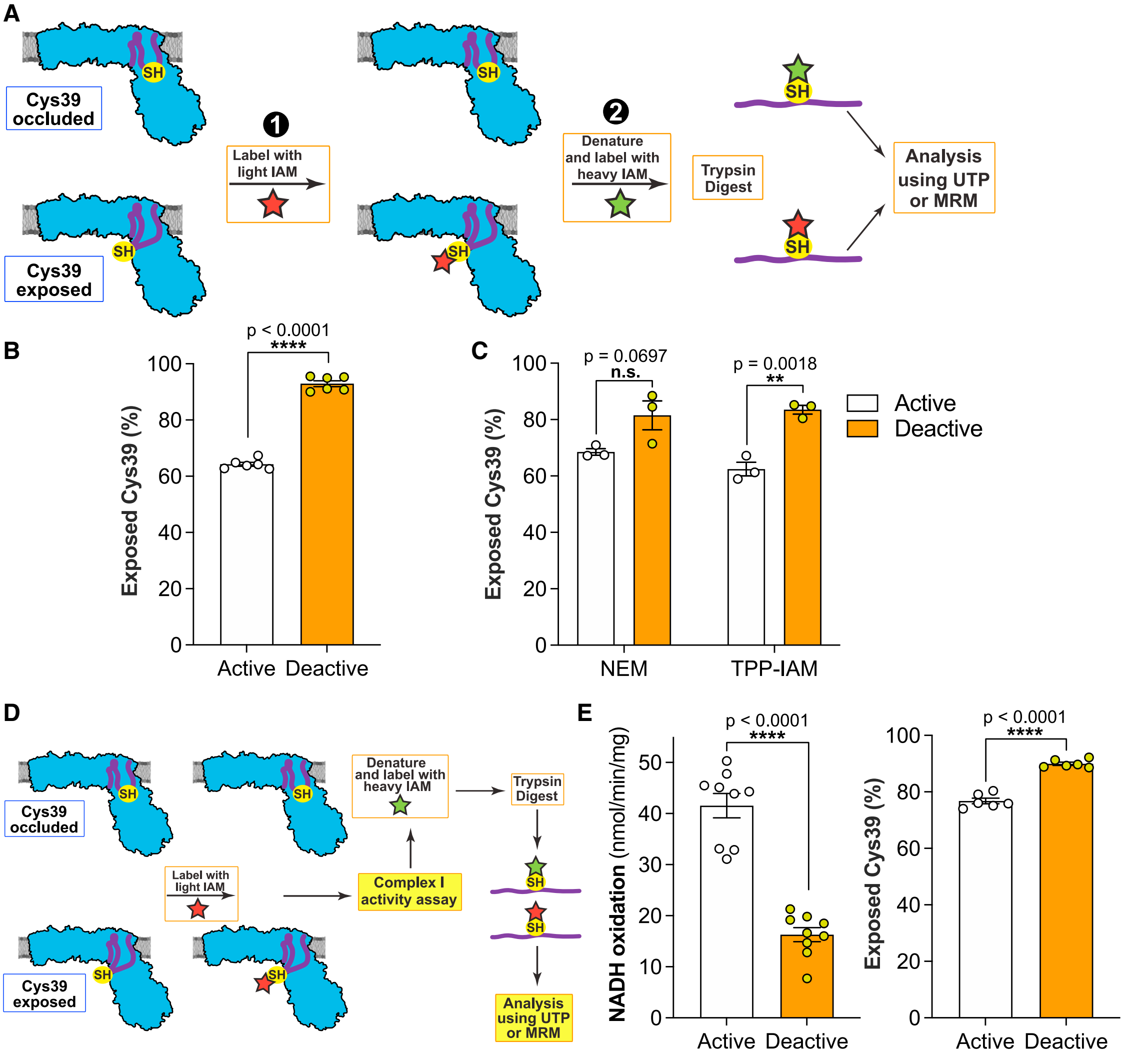

Figure 3. Cys39 is exposed in catalytically active complex I

(A) Schematic of the differential labeling strategy employed to quantify Cys39 exposure by LC-MS.

(B) Proportion of exposed Cys39 by catalytically active and deactive complex I in BHMMs labeled with 20 mM IAM for 5 min on ice. Data are represented as mean ± SEM (n = 6) processed in two independent experiments. Data were evaluated using an unpaired Student's t test.

(C) Proportion of exposed Cys39 by catalytically active (kept on ice prior to activation with NADH) and deactive complex I in BHMMs labeled with 10 mM d_5-NEM for 5 min on ice or 10 mM TPP-IAM for 5 min at room temperature (active samples in presence of 0.2 mM NADH). Data are represented as mean ± SEM (n = 3). Data were evaluated using an unpaired Student's t test.

(D) Schematic of sequential complex I activity and Cys39 exposure measurements. Related to Figures 3E, 5E, 5H, S3E, and S3F.

(E) Complex I activity and proportion of Cys39 exposure by catalytically active and deactive complex I in BHMMs in a combined sequential analysis upon labeling with 20 mM IAM for 5 min on ice, followed by washing with 1 mM GSH. Data are presented as mean ± SEM (complex I activity: n = 9; Cys39 exposure n = 6). Data were obtained from three independent experiments. Data were evaluated using an unpaired Student's t test.

Our finding of Cys39 exposure in the catalytic A-state disagrees with the original study that found Cys39 was only exposed in the catalytic D-state (Galkin et al., 2008). An alternative explanation is that inhibition of catalytic D-form reactivation by thiol alkylation reagents is due to a cysteine residue other than ND3 Cys39, which is fully occluded in the catalytic A-state and fully exposed in the catalytic D-state. However, such a residue might have been expected to have been detected by the fluorescent tagging in earlier reports (Galkin et al., 2008), where only a single band consistent with migration of ND3 was detected. We analyzed conserved residues among species that undergo the A/D transition, calculated the surface exposure of γ-sulfur atoms of complex I cysteines using the cryoEM structural active (PDB:6G2J) and deactive (PDB:6G72) mouse complex I (Agip et al., 2018), and carried out an liquid chromatography-mass spectrometry (LC-MS) survey of complex I peptides containing cysteine residues that were differentially labeled by sequential reaction with L-IAM and H-IAM in the A- and D-states (Table S1). Of the 116 unique cysteine residues in bovine complex I, of which 114 were found to be conserved in mice and therefore included into the analysis, we quantified the exposure status of 43 (37.7%; see Tables S1 and S2 for details/quantifications)

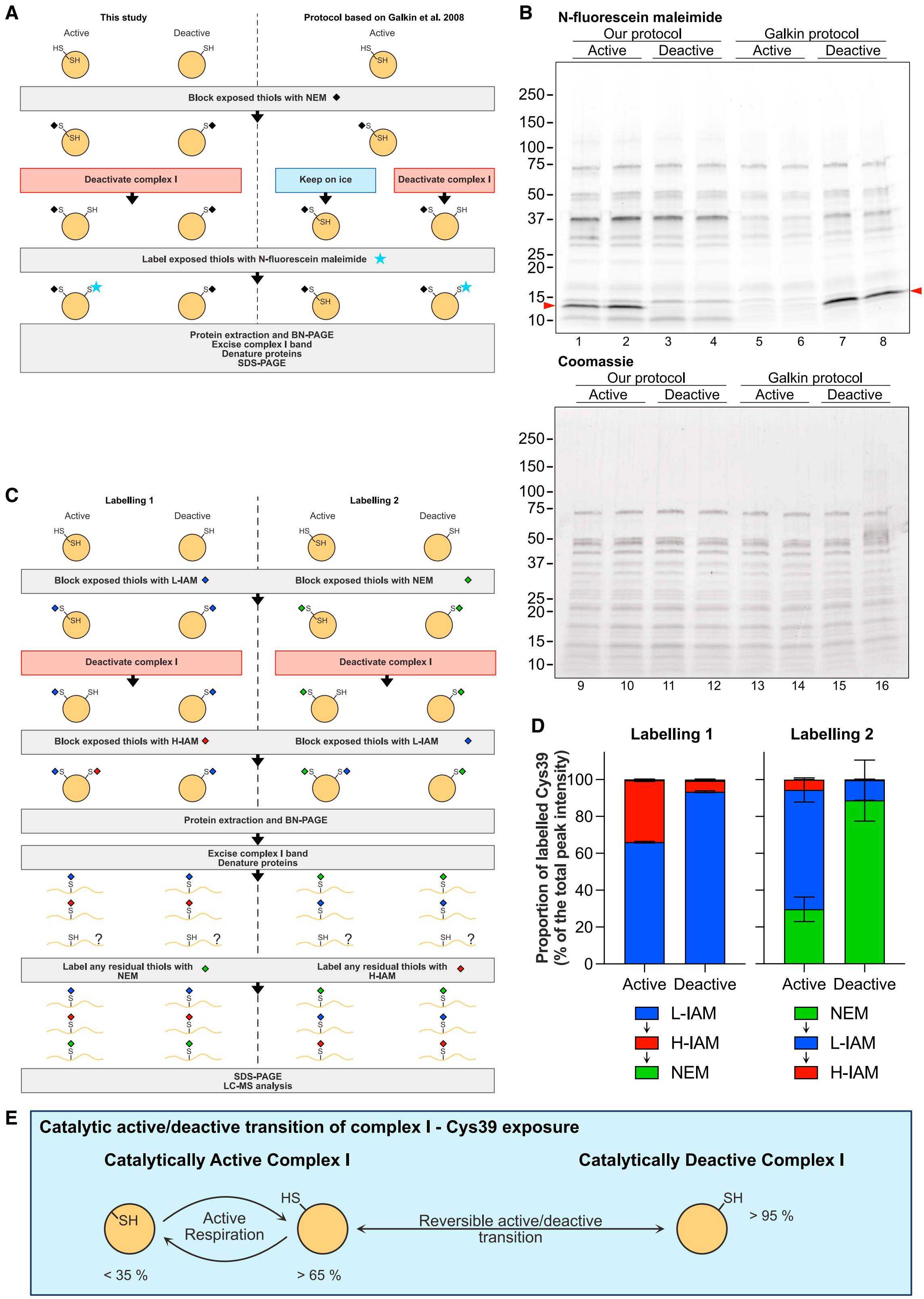

Figure 4. Comparison of Cys39 exposure by fluorescence and quantitative mass spectrometry

(A) Schematic of the fluorescent labeling approach for Cys39. Two different labeling strategies were applied, followed by separation of labeled proteins via BN-PAGE. Proteins in the complex I band were then resolved by SDS-PAGE.

(legend continued on next page)

with none matching the expected pattern. Of the 62 residues with no data, 19 (30.7%) form FeS centers, and 18 (29.0%) are predicted/modeled to form intramolecular disulfides. A total of 32 (51.6%) of the residues without data were classified as undetectable due to the properties of the tryptic peptides. This leaves 71 (62.3% of total) unquantified potential candidate cysteines, of which 44 (62.0%) form FeS centers or are predicted/modeled to form intramolecular disulfides (Table S2). However, none of the remaining 27 unquantified residues (of which eight are within proteins of a size that might match the fluorescent signal) showed a marked increase in solvent accessibility of the γ-sulfur atom in the structural deactive compared with the structural active mouse structure (Table S1). This makes it most probable that the inhibitory effect of thiol alkylating reagents on the reactivation of the catalytic D-form is due to ND3 Cys39 alkylation.

We next sought to reconcile our results with the earlier observation of no Cys39 exposure in the catalytic A-state (Galkin et al., 2008) (Figure 4A). In that approach, complex I in the catalytic A-state was reacted with NEM, then one portion was kept on ice while the other was deactivated, and thiols exposed following the catalytic A/D transition were labeled with the thiol reactive N-fluorescein maleimide, followed by Blue native- (BN-) and SDS-PAGE and interrogation of the fluorescently labeled bands (Galkin et al., 2008). This showed labeling of Cys39, which we replicated with our protocol (Figure 4B). We further extended this study using a different fluorescent tag (Cy5 maleimide) while labeling under three different conditions (Figures S4A–S4D). With both methods, fluorescent labeling of Cys39 was only observed in samples where the catalytic A-state was labeled with NEM, followed by deactivation and then fluorescent labeling. This is consistent with a large proportion of Cys39 residues being exposed in the catalytic A-state that NEM modifies and renders undetectable. Next, we applied our UTP approach to quantify Cys39 exposure using a sample preparation equivalent to that used in our fluorescent labeling approach (Figure 4C). The protocol was extended using two different labeling sequences: labeling exposed Cys39 with L-IAM or NEM, deactivation and labeling with H-IAM or L-IAM, BN-PAGE followed by denaturing and labeling of any residual free Cys39 with NEM or H-IAM. These two distinct labeling sequences were necessary to quantify all cysteine residues, as MS detection of NEM-labeled ND3 peptides may differ from that of IAM-labeled peptides, thereby distorting relative peptide levels. MS analysis of the first labeling sequence again showed that there was a significant amount of Cys39 exposed in the catalytic A-state, but with some further Cys39 residues becoming accessible upon deactivation, corresponding to the signal detected by fluorescent labeling (Figure 4D). In addition, in the earlier study, NADPH was used to activate complex I (Galkin et al., 2008). We found that NADPH decreased Cys39 exposure to a greater extent than NADH (Figure S4E). Hence, the experimental approach used earlier is technically valid (Galkin et al., 2008), but the alkylation protocol renders invisible any Cys39 that is exposed in the catalytic A-state. Therefore, the conclusion that Cys39 is only exposed in the catalytic D-state of complex I is incorrect. Instead, in the catalytic A-state a significant proportion of Cys39 is reactive with alkylating agents, with more becoming reactive upon deactivation (Figure 4E).

Effect of CoQ redox state and complex I inhibitors on Cys39 exposure

To explore how Cys39 exposure correlated with CoQ redox state, we switched to analyzing NADH-driven respiration in BHMMs (Figure 5A), where electrons are passed via the endogenous CoQ pool onto O_2. During respiration on NADH, when the CoQ pool is expected to be relatively oxidized (Burger et al., 2020), Cys39 was largely exposed. For BHMMs respiring on succinate, Cys39 was almost completely exposed. In membranes that were deactivated, Cys39 was largely exposed and subsequent addition of NADH to reactivate complex I decreased Cys39 exposure to the level of membranes oxidizing NADH, while succinate with antimycin A, to fully reduce the CoQ pool, did not decrease Cys39 exposure. Analysis by MRM and UTP of mouse heart mitochondrial membranes (MHMMs) as isolated, without further treatment, showed that complex I Cys39 was largely occluded (Figure 5B), consistent with a previous structural study (Agip et al., 2018). When complex I in MHMMs was converted to the catalytic D-state, ~62% of Cys39 was exposed (Figure 5B). However, during NADH-driven respiration, Cys39 was ~39% exposed (Figure 5C). Although Cys39 exposure was qualitatively the same in bovine and mouse mitochondrial membranes, there seems to be a quantitative difference in exposure or reactivity of Cys39 between the two species.

Next, we explored complex I Q-site inhibitors, which are thought to reorder the Q channel and force complex I into a state in which Cys39 is largely occluded, as shown by CryoEM (Blaza et al., 2018; Bridges et al., 2020; Grivennikova et al., 1997). Cys39 exposure was markedly decreased by rotenone and even more so by piericidin A (Figures 5A and 5C). Importantly, the high extent of Cys39 occlusion by piericidin A (Figures 5C and 5F) indicates that our methodology can cover a large dynamic range (from ~10% to 95%) and the low level of Cys39 occlusion seen earlier was not an artifact of the experimental procedures used. Reactivating complex I from the catalytic D-state by NADH decreases Cys39 exposure, whereas

(B) Fluorescent scan (top) of labeled proteins after differential labeling with NEM and N-fluorescein maleimide as detailed in Figure 4A. Proteins were first separated by BN-PAGE followed by separation of the proteins within the complex I band by SDS-PAGE (labeled ND3 indicated with red arrows). As loading control the same gel was stained with Coomassie (bottom).

(C) Schematic of the differential labeling approach for Cys39 for quantitative LC-MS (UTP) analysis. Two different labeling regimens were employed to allow for the quantification of Cys39 at different stages throughout the protocol. Labeled native proteins were separated by BN-PAGE followed by denaturing of the proteins within the complex I band, reduction and labeling of any residual unlabeled cysteines and subsequent SDS-PAGE separation. Proteins were cleaved in-gel with trypsin and analyzed by LC-MS.

(D) Proportions of Cys39 in complex I of BHMMs labeled with NEM or H- or L-IAM following the differential labeling approach detailed in Figure 4C. The proportion of peak areas of differentially labeled ND3 peptides out of the sum of all peak areas is shown. Data are mean ± range of two independently processed samples per condition.

(E) Schematic of the proposed dynamics of Cys39 exposure by active and deactive complex I. During complex I respiration, Cys39 is exposed to a large extent with some Cys39 remaining occluded. Cys39 is completely exposed in catalytically deactive complex I.

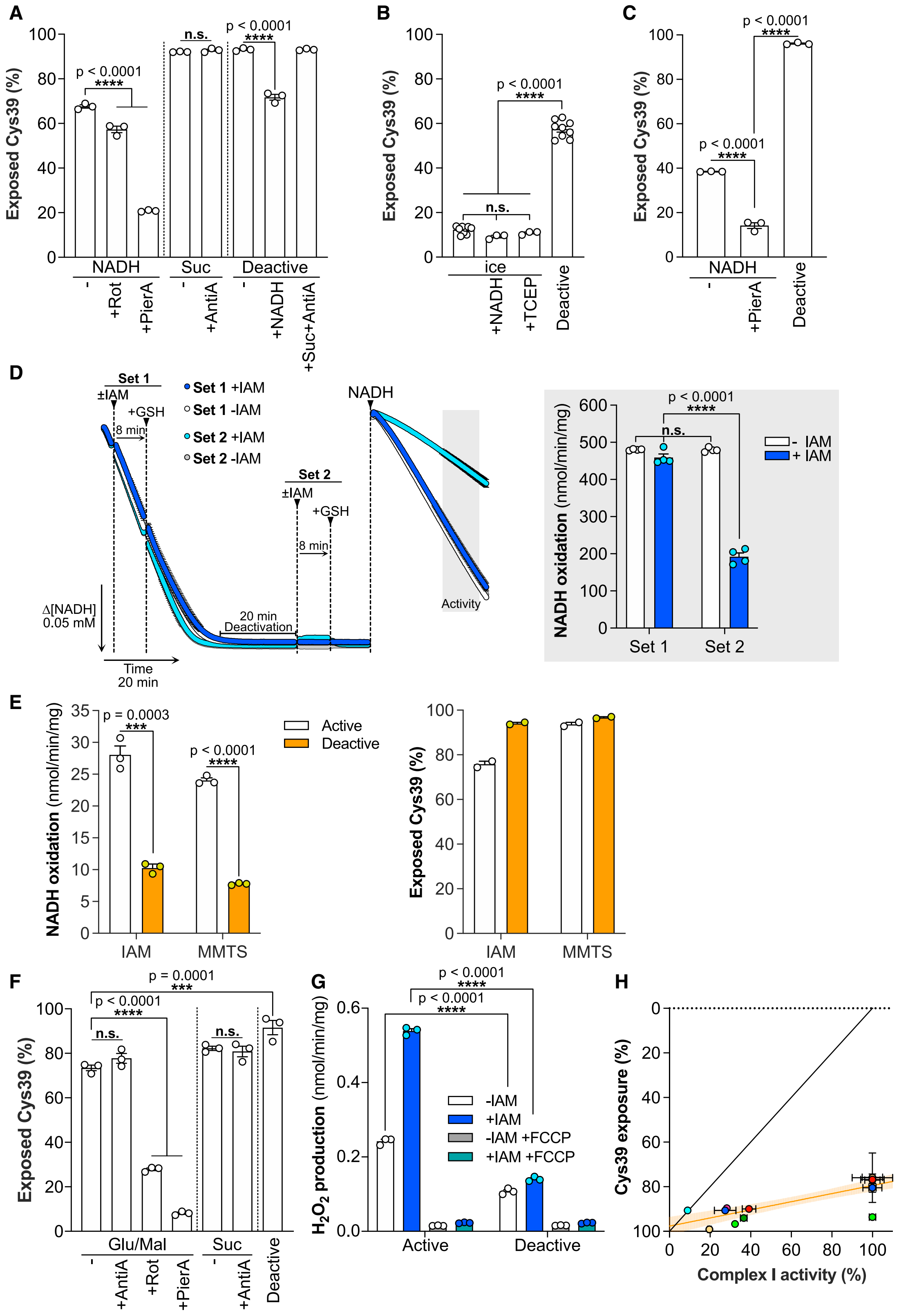

Figure 5. Cys39 is exposed during complex I respiration irrespective of the protonmotive force and CoQ redox state

(A) Cys39 exposure by complex in BHMMs during net turnover conditions respiring on NADH or succinate (10 mM each), supplemented with rotenone (2 μM), piericidin A (2 μM), or antimycin A (5 μM) if indicated. Deactive samples were incubated for 20 min at 37°C prior to addition of indicated substrates. Exposed

(legend continued on next page)

reduction of the CoQ pool by succinate does not (Figure 5A). Thus, we conclude that Cys39 is significantly exposed during NADH/CoQ oxidoreductase activity, that CoQ redox state does not impact on the extent of this exposure and that the complex I inhibitor piericidin A locks complex I into a state where Cys39 is largely occluded.

Exploring the D/A transition after Cys39 alkylation of the catalytic A-state

We next assessed whether alkylation of Cys39 during NADH/O_2 oxidoreductase activity prevented subsequent deactivation. To do this, complex I in BHMMs was labeled with IAM either during active respiration, or after deactivation. Then we quenched unreacted IAM with glutathione (GSH), followed by replenishing NADH and assessing complex I activity. Remarkably, complex I, labeled during active net turnover and then deactivated, was subsequently reactivated to the same extent as the control. Thus, Cys39 labeling under turnover conditions prevents complex I from becoming locked irreversibly in a catalytically inactive state following exposure to conditions that promote deactivation (Figure 5D). We conclude that alkylating Cys39 of the catalytic D-state locks complex I in a catalytically inactive state from which it cannot be reactivated. In contrast, labeling of Cys39 during turnover does not block complex I activity. Then, the labeled complex I can potentially undergo a cycle that would normally lead to its deactivation and reactivation, without losing NADH/CoQ oxidoreductase activity. However, in this case it is unclear if the labeled active enzyme adopts a “catalytic D-like” state upon deactivation, or if alkylation locked it permanently in the catalytic A-state.

Assessment of complex I Cys39 exposure in mitochondria

In mitochondrial membranes, the NADH/CoQ oxidoreductase activity is presumably coupled to proton pumping, but this will not be associated with the development of a protonmotive force (Δp) in the absence of an intact mitochondrial inner membrane. To explore the effects of Δp on Cys39 exposure and the A/D transition, we measured Cys39 exposure in rat heart mitochondria (RHM). The catalytic D-state was generated by incubating RHM at 37°C to deplete endogenous substrates, while for reactivation intramitochondrial NADH was generated by glutamate and malate in the presence of ADP. Exposed cysteines were labeled, and NADH/dQ oxidoreductase activity and Cys39 exposure were measured after permeabilization with alamethicin to allow access of NADH (Figures 3D and 5E). Combining the assessment of complex I activity and Cys39 exposure in isolated mitochondria showed that catalytically active complex I was extensively labeled by IAM or MMTS without affecting its activity, while in the catalytic D-state there is a further increase in Cys39 exposure (Figure 5E). Then, we assessed Cys39 exposure in RHM respiring on glutamate/malate or succinate, upon addition of inhibitors and also in mitochondria treated to convert complex I to the catalytic D-state. We found that Cys39 was highly exposed under these conditions, except when rotenone or piericidin A was present (Figure 5F). Reducing the CoQ pool with succinate, or in combination with antimycin A, did not occlude Cys39, while deactivation of complex I increased Cys39 exposure. Next, we measured the ROS production associated with reverse electron transport (RET) in RHM (Figure 5G). As expected, RHM respiring on succinate led to extensive ROS production, while in deactivated RHM, addition of succinate did not increase ROS (Figure 5G). Treatment of active RHM with IAM under conditions that will label the catalytic A-state of complex I did not impair RET. In fact, ROS production increased, possibly due to inhibition of thiol-dependent peroxidases. In contrast, IAM labeling only slightly increased the very low ROS production in mitochondria treated to convert complex I to its catalytic D-state.

cysteines were labeled with 20 mM IAM starting 1 to 1.5 min after initiating respiration for 5 min at 37°C. Data are presented as mean ± SEM of three independently processed replicates. Data were evaluated using a 1-way ANOVA test with Tukey’s multiple comparisons correction.

(B) Proportion of exposed Cys39 (as prepared) and by catalytically active (+NADH) and catalytically deactive (incubated for 30 min at 37°C prior to labeling) complex I in MHMMs labeled with 20 mM IAM, if indicated in the presence of 5 mM TCEP, for 5 min on ice. Data are represented as mean ± SEM (n = 3–9). Data were evaluated using a 1-way ANOVA test with Tukey’s multiple comparisons correction.

(C) Cys39 exposure by complex I in MHMMs during turnover conditions respiring on NADH, supplemented with piericidin A (2 μM) if indicated. Deactive samples were incubated for 30 min at 37°C prior to labeling. Exposed cysteines were labeled with 20 mM IAM starting 1 to 1.5 min after initiating respiration for 5 min at 37°C. Data are presented as mean ± SEM (n = 3). Data were evaluated using a 1-way ANOVA test with Tukey’s multiple comparisons correction.

(D) NADH oxidation (NADH:O_2 oxidoreductase activity) in BHMMs upon labeling with 5 mM IAM for 8 min during net turnover (Set 1) or after deactivation for 20 min following NADH depletion (Set 2). The labeling was quenched upon addition of 20 mM GSH. After labeling for both sets was completed, NADH was replenished and NADH oxidation rates were quantified (light gray section; right). Data are presented as mean ± SEM (n = 4). Data were evaluated using a 2-way ANOVA test with Tukey’s multiple comparisons correction.

(E) NADH/dQ oxidoreductase activity and proportion of Cys39 exposure by catalytically active and deactive complex I in RHM in a combined sequential analysis upon labeling with 20 mM IAM or 0.5 mM MMTS for 5 min on ice. Data are presented as mean ± SEM (complex I activity: n = 3) or mean ± range (Cys39 exposure n = 2). Data were evaluated using an unpaired Student’s t test.

(F) Cys39 exposure by complex I in RHM respiring on indicated substrates (10 mM) with addition of antimycin A (5 μM), rotenone (2 μM), or piericidin A (2 μM), if indicated. Deactive samples were incubated for 30 min without substrates at 37°C. Shortly after initiation of respiration (1.5 min or after 30 min of deactivation) 20 mM IAM was added and samples were labeled for 10 min at 37°C during active respiration. Data are presented as mean ± SEM of three independently processed replicates. Data were evaluated using a 1-way ANOVA test with Tukey’s multiple comparisons correction.

(G) ROS formation via RET in catalytically active and deactive RHM. Mitochondria were labeled with 20 mM IAM for 5 min on ice. Succinate (10 mM) driven ROS production by complex I was measured via AmplexRed. Data are presented as mean ± SEM of three independently processed samples that were each measured in triplicate. Data were evaluated using a 2-way ANOVA test with Tukey’s multiple comparisons correction.

(H) Correlation of complex I activity and Cys39 exposure in catalytically active and deactive BHMMs and RHM upon labeling of exposed thiols with 20 mM IAM or 0.5 mM MMTS for 5 min on ice in different buffers. The line of optimal inverse correlation and a linear regression line with 95% confidence including all datapoints are shown. The complex I activity in catalytically active samples was set to 100%. Data are presented as mean ± SEM or mean ± range (for n = 2) (complex I activity: n = 3–9; Cys39 exposure n = 2–6). Combined representation of Figures 3E, 5E, S3E, and S3F (each condition/experiment is indicated with a different color that is used for both active and deactive samples).

We conclude that within mitochondria when complex I is actively turning over, Cys39 is partially exposed. In particular, conditions that allow RET (succinate ± antimycin A) and conditions ± large Δp (modulated using antimycin A) do not affect Cys39 labeling. Therefore, Cys39 exposure is not determined by RET, CoQ redox state, or Δp. Furthermore, addition of IAM under conditions that label Cys39 while complex I is in the catalytic A-state do not block RET, suggesting that alkylation of Cys39 does not decouple electron transport from proton pumping, at least during RET.

Correlation of Cys39 exposure with complex I NADH/CoQ oxidoreductase activity

We combined sequential measurements of complex I NADH/dQ oxidoreductase activity and Cys39 exposure from the range of experiments assessed here with complex I in catalytic A- and D-states in BHMMs and RHM. From this, a clear pattern emerged with all points lying well below the line expected if the measurement of the catalytic A to D transition by enzyme activity directly correlated with Cys39 exposure (Figure 5H). We conclude that there is a correlation between the catalytic A- and D-states of complex I and Cys39 exposure, but that Cys39 exposure is not a unique characteristic of the catalytic D-state.

Contribution of damaged complex I to quantification of Cys39 exposure

The data described above from BHMMs, MHMMs, and isolated mitochondria suggest that Cys39 is partially exposed in complex I undergoing net turnover. As this was unexpected, we carried out extensive technical controls to validate our MS quantification of Cys39 alkylation (see method details: Labeling of Cys39 during respiration). However, the possibility remains that our samples also contained a pool of damaged, catalytically inactive complex I, or of partially assembled/degraded complex I, that contained exposed ND3 Cys39. In this scenario, alkylation of a catalytically active complex I preparation would, through modification of the catalytically inactive complex I, give the false impression of Cys39 alkylation of complex I during respiration. However, interrogation of the data presented above suggests that this possibility is unlikely, for the following reasons:

1 In Figure S3D, following labeling with IAM of the catalytically active or deactive complex I, the intact complex was isolated by BN-PAGE and then further analyzed by MS. This showed greater than 80% Cys39 exposure in the catalytically active sample under these conditions, where any contribution from unincorporated ND3 subunits, or partially assembled/degraded complex I are eliminated.
2 In Figure 5A, BHMMs, that are incubated directly from frozen with NADH, the exposure of Cys39 is the same as if they had been deactivated for 20 min at 37°C and then reactivated with NADH. Thus, any accumulation of damaged complex upon incubation at 37°C for 20 min does not contribute to increased Cys39 alkylation.
3 In Figure 5A, the presence of the Q-site inhibitor piericidin A, which forces complex I to occlude Cys39, the Cys39 exposure decreases from ~70% to ~20%, suggesting that at most 20% of complex I could be damaged and contribute artifactually to the Cys39 alkylation. Furthermore, the effect of piericidin A was rapid, as it was only added 1 to 1.5 min prior to addition of IAM, so its effect was not due to stabilizing complex I in some way in comparison with the deactive incubation, which was for 20 min at 37°C. The results with MHMMs in Figure 5C showed a qualitatively similar effect of piericidin A.
4 In Figures 5E and 5F, freshly isolated heart mitochondria also show ~75% exposure of Cys39 when complex I is turning over.
5 When freshly isolated heart mitochondria are actively respiring on the NADH-linked substrates glutamate and malate, addition of piericidin A decreases Cys39 exposure from about 75% to about 10% (Figure 5F). So, again, the maximum proportion of damaged complex I that could be contributing to the measurement of Cys39 alkylation during turnover of complex I is less than 10%.

Therefore, we conclude that it is unlikely that alkylation of Cys39 in pools of damaged or partially assembled complex I contributes significantly to our finding of Cys39 exposure in complex I during respiration.

Assessing the A/D transition in mouse tissues during ischemia and reperfusion

Finally, we assessed the complex I catalytic A/D transition and Cys39 exposure within mouse tissues. To do this, tissues were rapidly removed and clamp frozen after having been maintained under normoxic or ischemic conditions. The Cys39 exposure state and complex I NADH/dQ oxidoreductase activity measurements were optimized in tissue homogenates (method details: Tissue homogenate and Figures S5 and S6). Comparison of CI activity for normoxic and ischemic tissues against Cys39 exposure showed a clear inverse correlation between declining activity and increasing Cys39 exposure (Figure 6A). This suggests that complex I shifts from the catalytic A-state to the catalytic D-state during ischemia. We also found that the Cys39 exposure by complex I was somewhat decreased in the supercomplex fraction compared to the monomeric fraction (Figure S6E). This distribution was also maintained after exposure to ischemia but with overall increased Cys39 exposure. As Cys39 is on the loop between ND3 transmembrane helices (TMH) 1 and 2 and in supercomplexes TMH1 is on the side of the complex III dimer interface with complex I, lipid packing might help to stabilize this helix and therefore the TMH1–2 loop (Guo et al., 2017); however, further exploration of this potentially interesting finding is beyond the scope of this work.

Next, we explored how rapidly complex I activity declined in mouse heart during ischemia. There was rapid Cys39 exposure in the first minutes of ischemia in the heart and brain, which reached a plateau after 20 min, consistent with previous literature (Gorenkova et al., 2013) (Figures 6B and 6C). Combining data from heart and brain indicated that complex I activity and Cys39 exposure during ischemia correlated to the same extent in both organs (Figure 6D).

This was extended to assess changes in complex I upon reperfusion of the ischemic heart *in vivo* (Figure 6E). Our data revealed inhibition of complex I activity and increase in Cys39 exposure upon ischemia, which was reversed upon 5 min of reperfusion, with severe complex I impairment after a further 2 h of

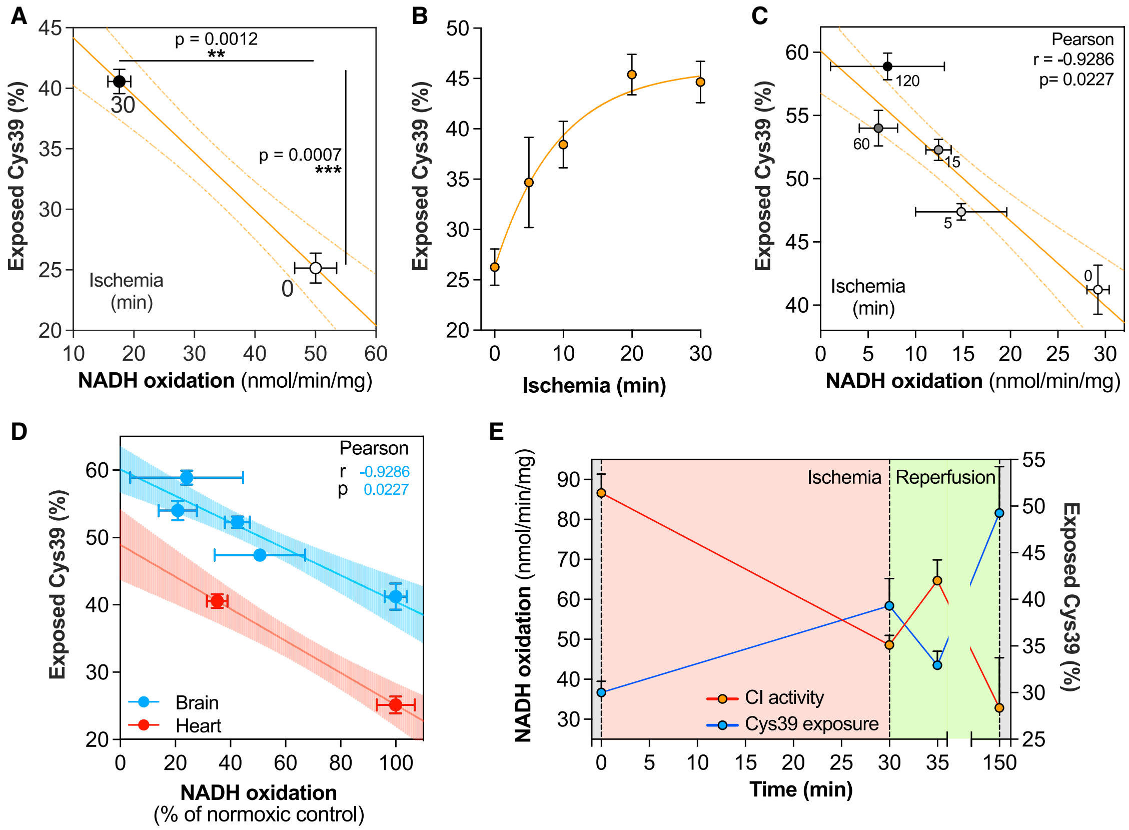

Figure 6. Complex I Cys39 exposure and activity during ischemia and reperfusion in tissues

(A) Correlative representation of NADH/dQ oxidoreductase activity and Cys39 exposure in normoxic and ischemic mouse heart. NADH oxidation was assessed in mouse heart homogenate in the presence of 0.025% *n*-Dodecyl D β-maltoside (DDM). Cys39 exposure was assessed following labeling of exposed thiols with 20 mM of IAM for 5 min on ice. Data are presented as mean ± SEM of three individual hearts. Data were evaluated using an unpaired Student's t test.

(B) Cys39 exposure by complex I in mouse heart upon increasing length of ischemia. Exposed thiols were labeled with 20 mM IAM for 5 min on ice. Data are presented as mean ± SEM (n = 5–8) per time point.

(C) Correlative representation of NADH/dQ oxidoreductase activity and Cys39 exposure in normoxic and ischemic mouse brain. NADH oxidation was assessed in mouse brain homogenate in the presence of 0.025% DDM. Cys39 exposure was assessed after labeling of exposed thiols with 20 mM of IAM for 5 min on ice. Data are presented as mean ± SEM of three individual brains.

(D) Correlative representation of NADH/dQ oxidoreductase activity (normalized to normoxic control) and Cys39 exposure in normoxic and ischemic mouse heart and brain. NADH oxidation activity was assessed in tissue homogenate in the presence of 0.025% DDM. Cys39 exposure was assessed by labeling of exposed thiols with 20 mM of IAM for 5 min on ice. Data are presented as mean ± SEM of three individual hearts or brains. Figure related to Figures 6A and 6C.

(E) Cys39 exposure and NADH/dQ oxidoreductase activity were measured in risk area of mouse hearts on which the left anterior descending coronary artery myocardial infarct model was performed. NADH oxidation activity was assessed in heart homogenate in the presence of 0.025% DDM. Cys39 exposure was assessed after labeling of exposed thiols with 20 mM of IAM for 5 min on ice. Data are presented as mean ± SEM of three individual hearts per time point.

reperfusion, presumably due to the ROS production associated with IR injury damaging complex I.

DISCUSSION

Mammalian complex I is the principal entry point for electrons into the respiratory chain, while also being a major source of ROS in physiological signaling and in pathology. Biochemical and structural studies suggested a binary model, in which complex I can adopt two distinct functional states, a catalytically active A-state and a catalytically inactive D-state, in a process termed the catalytic A/D transition. These states had been defined by occlusion (catalytic A-state) and exposure (catalytic D-state) of the ND3 Cys39 residue. This view was consistent with CryoEM studies that determined a structural A-state in which Cys39 was occluded and a structural D-state in which Cys39 was presumed to be solvent exposed.

Here we applied quantitative proteomics to demonstrate that Cys39 is largely exposed in the catalytic D-state in agreement with previous studies. However, a large proportion of Cys39 is also exposed when complex I is active. This finding stands in stark contrast to the generally accepted binary model (Figure 1). Furthermore, we demonstrated that only complex I residing in a catalytic D-state can be irreversibly inactivated by alkylation of Cys39. In contrast, alkylation of exposed Cys39 during respiration did not impair NADH/CoQ reductase activity. If Cys39 exposure occurs during normal respiration by complex I, we would expect to find complete alkylation of Cys39; however, the maximum alkylation we found was ~80%. The reasons for this are unclear, but may be due to incomplete reaction of Cys39

under our conditions. It is also unclear if the Cys39 exposed on complex I during respiration occurs as an intermediate in the catalytic cycle of complex I, or as a transient off-pathway state. Future experiments on purified complex I in fully defined liposome systems will be required to address this point. In summary, this work provides unexpected and fundamental insights into the catalytic A/D transition and the role of Cys39 in the mechanism of complex I.

While Cys39 was regarded as a key indicator for the complex I state (Galkin et al., 2008), it is likely to be only one feature of a broad set of concerted conformational rearrangements within the enzyme throughout the catalytic A/D transition. Some of the potential conformational changes were recently visualized in CryoEM structures of complex I that identified two major classes of the enzyme, which were correlated with complex I activity and therefore were proposed to closely match the structures of complex I in the catalytic A- and D-states (Agip et al., 2018; Blaza et al., 2018; Bridges et al., 2020; Fiedorczuk et al., 2016; Grba and Hirst, 2020; Yin et al., 2021; Zhu et al., 2016). The two structural A- and D-states of complex I show that in the structural, resting A-state, the ND3 loop between TMH1 and 2 is on the matrix-facing surface of the hinge region between the membrane and matrix arms, with Cys39 occluded in a pocket formed by ND1, NDUFS2, and NDUFS7. In contrast, in the structural D-state, the ND3 loop is disordered and not resolved by CryoEM, suggesting that Cys39 is exposed to the solvent. Our data show that this binary model does not necessarily apply to catalytically active complex I. Instead, we show that Cys39 is exposed, suggesting that these CryoEM studies only captured two resting states and that there are likely to be other states with different levels of exposure of Cys39. Interestingly, we found that the inhibitor piericidin A stabilized complex I in a conformational state with fully occluded Cys39, which is in agreement with the structure of piericidin A-bound complex I in the structural A-state (Bridges et al., 2020). That binding of piericidin A into the Q site mediates Cys39 occlusion is consistent with the suggestion that Cys39 occlusion and complex I activation may be mediated by the ubiquinone head group of CoQ acting as a template, around which the CoQ binding site can reform (Blaza et al., 2018). It is also particularly interesting to note that in complex I containing a mutation in its ND6 subunit, Cys39 is susceptible to alkylation when active (Yin et al., 2021) but that in this case alkylation leads to irreversible inhibition of complex I.

Furthermore, we also showed that Cys39 exposure was independent of Δp or the CoQ redox state. This constrains a potential role for Cys39 exposure as part of the normal catalytic cycle of complex I. In recent structural work, Kampjut and Sazanov (2020) describe different structural states of complex I. They suggest that "closed" and "open" conformations (roughly equivalent to the structural A- and D-states) exist as on-pathway catalytic turnover states, and propose a distinct deactive resting state in which a tilt of ND6 TMH4 inserts the TMH3-4 loop between the membrane and matrix arm. In this and previous work (Kampjut and Sazanov, 2020; Letts et al., 2019), a large proportion of complex I was found in an "open" conformation with a disordered ND3 TMH1-2 loop exposing Cys39 in catalytically active complex I preparations. However, the assignments of these "open" states as catalytic intermediates have been questioned, linked to the suggestion that they may instead represent structural states occurring during the catalytic A/D transition (Hirst and Kaila, 2021).

We showed that Cys39 alkylation of respiring complex I does not impair electron transfer from NADH to CoQ. In contrast, labeling exposed Cys39 of complex I in the catalytic D-state locks the enzyme in a catalytically inactive state and prevents the enzyme from re-entering the catalytic cycle. This suggests that there may be distinct structural features that are present in the catalytic D-state but not in the active Cys39-exposed state. We also showed that Cys39 alkylation has no effect on RET ROS production. As RET requires coupling of electron movement with that of protons driven by Δp (Murphy, 2009; Pryde and Hirst, 2011), this finding suggests that alkylation of Cys39 under these conditions does not uncouple electron and proton transfer, at least during RET. This contrasts with work from *Yarrowia lipolytica*, which showed that immobilizing the ND3 loop with a disulfide bond between its Cys40 (equivalent to mammalian Cys39) and a cysteine residue Q133C (introduced into PSST(NDUFS7)) locks the complex in a structural D-like state, uncoupling proton pumping from electron transport (Cabrera-Orefice et al., 2018). However, this disulfide locked the ND3 loop in a fixed position, which may not provide sufficient flexibility to couple proton pumping. In addition, the evolutionarily distance between yeast and mammals makes the functional relevance of this finding for mammals unclear. More detailed work to measure proton pumping by isolated mammalian complex I directly in energized vesicles will be required to address the role of Cys39 alkylation on complex I coupling.

The catalytic A/D transition of complex I is of great interest for understanding the role of complex I in ischemia and reperfusion. In addition, targeting Cys39 by *S*-nitrosation was protective against IR injury in mice by preventing complex I reactivation and RET following ischemia (Chouchani et al., 2013; Kim et al., 2018; Methner et al., 2014; Wilson et al., 2018). However, our findings suggest that labeling Cys39 of actively turning over complex I does not impair its function, which implies that strategies to selectively lock deactive complex I in a catalytically inactive state may also modify Cys39 of the active enzyme, but without affecting electron transfer.

In summary, we suggest a model for the role of Cys39 in the catalytic A/D transition of complex I (see *graphical abstract*). During respiration when complex I is catalyzing electron transfer, Cys39 is exposed, perhaps intermittently. This may be because during catalytic turnover, complex I cycles through different conformational state(s), during which Cys39 is exposed. Alternatively, during respiration, catalytically active complex I could be in equilibrium with transient state(s) in which Cys39 is exposed that will not be formally regarded as part of the catalytic cycle, once it is fully defined. Upon catalytic deactivation, complex I adopts an "off-pathway" resting catalytic D-state that exposes Cys39 completely. Alkylation of exposed Cys39 during the catalytic D-state locks the enzyme in an inactive state. In contrast, alkylation of Cys39 while in the catalytic A-state seems to lock complex I permanently into an electron transfer-competent conformation. These findings suggest that the mechanistic consequences of Cys39 alkylation are closely linked to the structural features of complex I. Future CryoEM investigations of complex I, and particularly of the ND6 mutated form of complex I (Yin et al., 2021), in which Cys39 has been alkylated during various

catalytic states, in various species, will enable comparison between the structural and catalytic A- and D-states and will likely provide insight into the role of Cys39 in the catalytic cycle of complex I.

SIGNIFICANCE

The catalytic A/D transition of complex I is a long-known but enigmatic phenomenon. The deactivation of complex I is marked by significant conformational changes, with solvent exposure of ND3 Cys39 believed to be a key indicator. Complex I is a major source of pathological ROS production in IR injury, and therapeutic approaches have been developed to target exposed Cys39 to prevent reactivation of complex I and ROS formation post-ischemia. To shed light on the dynamics of the catalytic A/D transition, we have developed mass spectrometry-based strategies to quantify Cys39 exposure *in vitro* and in tissues and correlate it with complex I activity. Here, we demonstrate that Cys39 is exposed during respiration by the active mammalian enzyme. However, only catalytically deactive complex I can be inhibited by alkylation of Cys39, locking complex I in a catalytically inactive conformation. In contrast, alkylation of exposed Cys39 during respiration does not impair complex I NADH/CoQ oxidoreductase activity. This implies that unique structural features of the catalytically deactive enzyme are involved in Cys39-mediated complex I inhibition. These findings have implications for the understanding of complex I function and the catalytic A/D transition.

STAR★METHODS

Detailed methods are provided in the online version of this paper and include the following:

- KEY RESOURCES TABLE
- RESOURCE AVAILABILITY
  - Lead contact
  - Materials availability
  - Data and code availability
- EXPERIMENTAL MODEL AND SUBJECT DETAILS
- METHOD DETAILS
  - Synthesis of labelled ND3 peptides
  - Synthesis of TPP-IAM and d_{15}-TPP-IAM
  - Clamp freezing of tissue samples
  - *In situ* heart ischemia model
  - LAD occlusion myocardial infarct model
  - *Ex vivo* brain ischemia model
  - Preparation of mitochondrial membranes
  - Mitochondrial isolations
  - Mitochondrial membrane incubations
  - Labelling of membranes for activity assays
  - Complex I activity assay
  - Citrate synthase activity assay
  - Differential thiol labelling for LC-MS
  - Fluorescent labelling of Cys39 exposure
  - Mitochondrial ROS measurements
  - Blue native (BN)-PAGE
  - SDS-PAGE
  - In-gel protein cleavage and desalting
  - In-solution protein cleavage
  - LC-MS analysis of labelled ND3 peptides
  - Untargeted proteomics (UTP)
  - Targeted MRM analysis of ND3 peptides
  - Iodoacetamide labelling kinetics of GSH
  - Comparative analysis of all cysteine residues
- QUANTIFICATION AND STATISTICAL ANALYSIS

SUPPLEMENTAL INFORMATION

Supplemental information can be found online at https://doi.org/10.1016/j.chembiol.2021.10.010.

ACKNOWLEDGMENTS

This research was funded by the Medical Research Council (MC_U105663142 [M.P.M.], MC_UU_00015/7 [M.P.M], and MR/P000320/1 [T.K.]) and by the Wellcome Trust (WT110158/Z/15/Z, 110159/Z/15/Z, RG88195, 202905/Z/16/Z, and 206171/Z/17/Z). A.M. is supported by the Swedish Research Council (2018-00623). A.A.I.N is supported by the University of Glasgow. We thank Judy Hirst, A.-Noor A. Agip, Daniel Grba, and Hannah R. Bridges (MRC-MBU) for useful discussion and critical feedback to this work. We thank Hiran A. Prag and Injae Chung for providing bovine and mouse heart mitochondrial membranes, respectively. For the purpose of open access, the author has applied a CC BY public copyright license to any Author Accepted Manuscript version arising from this submission.

AUTHOR CONTRIBUTIONS

N.B., A.M.J., and M.P.M. carried out study conception and design. N.B. designed, performed, and analyzed most experiments. M.L.-L. helped in method development. J.F.M, A.M., and O.S. assisted with *in vivo* and *ex vivo* experiments and tissue sampling. T.K. supervised mouse experiments. S.D and I.M.F. performed UTP analysis and assisted with data analysis. N.B. and S.A. developed, optimized, and performed MRM analysis of ND3 peptides. K.H. designed and synthesized ND3 peptides. A.A.I.N. and R.C.H. designed and synthesized TPP-IAM. The manuscript was written by N.B. and M.P.M. with assistance from all other authors. The study was directed by M.P.M.

DECLARATION OF INTERESTS

The authors declare no competing financial interest.

Received: May 22, 2021
Revised: July 21, 2021
Accepted: October 7, 2021
Published: November 4, 2021

REFERENCES

Agip, A.-N.A., Blaza, J.N., Bridges, H.R., Viscomi, C., Rawson, S., Muench, S.P., and Hirst, J. (2018). Cryo-EM structures of complex I from mouse heart mitochondria in two biochemically defined states. Nat. Struct. Mol. Biol. *25*, 548–556.

Antonucci, S., Mulvey, J.F., Burger, N., di Sante, M., Hall, A.R., Hinchy, E.C., Caldwell, S.T., Gruszczyk, A.V., Deshwal, S., Hartley, R.C., et al. (2019). Selective mitochondrial superoxide generation in vivo is cardioprotective through hormesis. Free Radic. Biol. Med. *134*, 678–687.

Babot, M., Birch, A., Labarbuta, P., and Galkin, A. (2014). Characterisation of the active/de-active transition of mitochondrial complex I. Biochim. Biophys. Acta *1837*, 1083–1092.

Belevich, N., and Verkhovskaya, M. (2016). Resting state of respiratory complex I from *Escherichia coli*. FEBS Lett. *590*, 1570–1575.

Belevich, N., Belevich, G., Chen, Z., Sinha, S.C., and Verkhovskaya, M. (2017a). Activation of respiratory complex I from *Escherichia coli* studied by fluorescent probes. Heliyon *3*, e00224.

Belevich, N., von Ballmoos, C., and Verkhovskaya, M. (2017b). Activation of proton translocation by respiratory complex I. Biochemistry *56*, 5691–5697.

Benson, E.S. (1955). Composition and state of protein in heart muscle of normal dogs and dogs with experimental myocardial failure. Circ. Res. *3*, 221–228.

Blaza, J.N., Vinothkumar, K.R., and Hirst, J. (2018). Structure of the deactive state of mammalian respiratory complex I. Structure *26*, 312–319.

Bridges, H.R., Fedor, J.G., Blaza, J.N., di Luca, A., Jussupow, A., Jarman, O.D., Wright, J.J., Agip, A.-N.A., Gamiz-Hernandez, A.P., Roessler, M.M., et al. (2020). Structure of inhibitor-bound mammalian complex I. Nat. Commun. *11*, 5261.

Burger, N., Logan, A., Prime, T.A., Mottahedin, A., Caldwell, S.T., Krieg, T., Hartley, R.C., James, A.M., and Murphy, M.P. (2020). A sensitive mass spectrometric assay for mitochondrial CoQ pool redox state in vivo. Free Radic. Biol. Med. *147*, 37–47.

Cabrera-Orefice, A., Yoga, E.G., Wirth, C., Siegmund, K., Zwicker, K., Guerrero-Castillo, S., Zickermann, V., Hunte, C., and Brandt, U. (2018). Locking loop movement in the ubiquinone pocket of complex I disengages the proton pumps. Nat. Commun. *9*, 4500.

Chouchani, E.T., Methner, C., Nadtochiy, S.M., Logan, A., Pell, V.R., Ding, S., James, A.M., Cochemé, H.M., Reinhold, J., Lilley, K.S., et al. (2013). Cardioprotection by S-nitrosation of a cysteine switch on mitochondrial complex I. Nat. Med. *19*, 753–759.

Chouchani, E.T., Pell, V.R., James, A.M., Work, L.M., Saeb-Parsy, K., Frezza, C., Krieg, T., and Murphy, M.P. (2016). A unifying mechanism for mitochondrial superoxide production during ischemia-reperfusion injury. Cell Metab. *23*, 254–263.

Clementi, E., Brown, G.C., Feelisch, M., and Moncada, S. (1998). Persistent inhibition of cell respiration by nitric oxide: crucial role of S-nitrosylation of mitochondrial complex I and protective action of glutathione. Proc Natl Acad Sci U S A *95*, 7631–7636.

Dröse, S., Stepanova, A., and Galkin, A. (2016). Ischemic A/D transition of mitochondrial complex I and its role in ROS generation. Biochim. Biophys. Acta *1857*, 946–957.

Ellman, G.L. (1959). Tissue sulfhydryl groups. Arch. Biochem. Biophys. *82*, 70–77.

Fedor, J.G., Jones, A.J.Y., di Luca, A., Kaila, V.R.I., and Hirst, J. (2017). Correlating kinetic and structural data on ubiquinone binding and reduction by respiratory complex I. Proc. Natl. Acad. Sci. U. S. A. *114*, 12737–12742.

Fernández-Vizarra, E., Ferrín, G., Pérez-Martos, A., Fernández-Silva, P., Zeviani, M., and Enríquez, J.A. (2010). Isolation of mitochondria for biogenetical studies: an update. Mitochondrion *10*, 253–262.

Fiedorczuk, K., Letts, J.A., Degliesposti, G., Kaszuba, K., Skehel, M., and Sazanov, L.A. (2016). Atomic structure of the entire mammalian mitochondrial complex I. Nature *538*, 406–410.

Galkin, A. (2019). Brain ischemia/reperfusion injury and mitochondrial complex I damage. Biochemistry *84*, 1411–1423.

Galkin, A., and Moncada, S. (2007). S-nitrosation of mitochondrial complex I depends on its structural conformation. J Biol Chem *282*, 37448–37453.

Galkin, A., and Moncada, S. (2017). Modulation of the conformational state of mitochondrial complex I as a target for therapeutic intervention. Interface Focus *7*, 20160104.

Galkin, A., Meyer, B., Wittig, I., Karas, M., Schägger, H., Vinogradov, A., and Brandt, U. (2008). Identification of the mitochondrial ND3 subunit as a structural component involved in the active/deactive enzyme transition of respiratory complex I. J. Biol. Chem. *283*, 20907–20913.

Gavrikova, E.V., and Vinogradov, A.D. (1999). Active/de-active state transition of the mitochondrial complex I as revealed by specific sulfhydryl group labeling. FEBS Lett. *455*, 36–40.

Gorenkova, N., Robinson, E., Grieve, D.J., and Galkin, A. (2013). Conformational change of mitochondrial complex I increases ROS sensitivity during ischemia. Antioxid. Redox Signal. *19*, 1459–1468.

Gostimskaya, I.S., Grivennikova, V.G., Zharova, T.V., Bakeeva, L.E., and Vinogradov, A.D. (2003). In situ assay of the intramitochondrial enzymes: use of alamethicin for permeabilization of mitochondria. Anal. Biochem. *313*, 46–52.

Grba, D.N., and Hirst, J. (2020). Mitochondrial complex I structure reveals ordered water molecules for catalysis and proton translocation. Nat. Struct. Mol. Biol. *27*, 892–900.

Grivennikova, V.G., Maklashina, E.O., Gavrikova, E.V., and Vinogradov, A.D. (1997). Interaction of the mitochondrial NADH-ubiquinone reductase with rotenone as related to the enzyme active/inactive transition. Biochim. Biophys. Acta *1319*, 223–232.

Grivennikova, V.G., Serebryanaya, D.V., Isakova, E.P., Belozerskaya, T.A., and Vinogradov, A.D. (2003). The transition between active and de-activated forms of NADH:ubiquinone oxidoreductase (Complex I) in the mitochondrial membrane of *Neurospora crassa*. Biochem. J. *369*, 619–626.

Guo, R., Zong, S., Wu, M., Gu, J., and Yang, M. (2017). Architecture of human mitochondrial respiratory megacomplex I2III2IV2. Cell *170*, 1247–1257.e12.

Hernansanz-Agustín, P., Ramos, E., Navarro, E., Parada, E., Sánchez-López, N., Peláez-Aguado, L., Cabrera-García, J.D., Tello, D., Buendia, I., Marina, A., et al. (2017). Mitochondrial complex I deactivation is related to superoxide production in acute hypoxia. Redox Biol. *12*, 1040–1051.

Hirst, J., and Kaila, V.R.I. (2021). Coupling mechanism of mammalian respiratory complex I remains elusive despite structural advances, eLetter https://science.sciencemag.org/content/370/6516/eabc4209/tab-e-letters.

James, A.M., Smith, A.C., Smith, C.L., Robinson, A.J., and Murphy, M.P. (2018). Proximal cysteines that enhance lysine N-acetylation of cytosolic proteins in mice are less conserved in longer-living species. Cell Rep. *24*, 1445–1455.

Jarman, O.D., Biner, O., Wright, J.J., and Hirst, J. (2021). *Paracoccus denitrificans*: a genetically tractable model system for studying respiratory complex I. Sci. Rep. *11*, 10143.

Kahl, A., Stepanova, A., Konrad, C., Anderson, C., Manfredi, G., Zhou, P., Iadecola, C., and Galkin, A. (2018). Critical role of flavin and glutathione in complex I–mediated bioenergetic failure in brain ischemia/reperfusion injury. Stroke *49*, 1223–1231.

Kalashnikov, D.S., Grivennikova, V.G., and Vinogradov, A.D. (2011). Submitochondrial fragments of brain mitochondria: general characteristics and catalytic properties of NADH:ubiquinone oxidoreductase (Complex I). Biochemistry *76*, 209–216.

Kampjut, D., and Sazanov, L.A. (2020). The coupling mechanism of mammalian respiratory complex I. Science *370*, eabc4209.

Kim, M., Stepanova, A., Niatsetskaya, Z., Sosunov, S., Arndt, S., Murphy, M.P., Galkin, A., and Ten, V.S. (2018). Attenuation of oxidative damage by targeting mitochondrial complex I in neonatal hypoxic-ischemic brain injury. Free Rad. Biol. Med. *124*, 517–524.

Kohlhauer, M., Pell, V.R., Burger, N., Spiroski, A.M., Gruszczyk, A., Mulvey, J.F., Mottahedin, A., Costa, A.S.H., Frezza, C., Ghaleh, B., et al. (2019). Protection against cardiac ischemia-reperfusion injury by hypothermia and by inhibition of succinate accumulation and oxidation is additive. Basic Res. Cardiol. *114*, 18.

Kotlyar, A.B., and Vinogradov, A.D. (1990). Slow active/inactive transition of the mitochondrial NADH-ubiquinone reductase. Biochim. Biophys. Acta *1019*, 151–158.

Kotlyar, A.B., Albracht, S.P.J., and van Spanning, R.J.M. (1998). Comparison of energization of complex I in membrane particles from *Paracoccus denitrificans* and bovine heart mitochondria. Biochim. Biophys. Acta *1365*, 53–59.

Letts, J.A., Fiedorczuk, K., Degliesposti, G., Skehel, M., and Sazanov, L.A. (2019). Structures of respiratory supercomplex I+III2 reveal functional and conformational crosstalk. Mol. Cell *75*, 1131–1146.

Luzikov, V.N., Saks, V.A., and Berezin, I.V. (1970). Comparative study of thermal degradation of electron transfer particles and reconstituted respiratory

chain. Relation of electron transfer to reactivation of submitochondrial particles. Biochim. Biophys. Acta *223*, 16–30.

Maklashina, E., Sher, Y., Zhou, H.-Z., Gray, M.O., Karliner, J.S., and Cecchini, G. (2002). Effect of anoxia/reperfusion on the reversible active/de-active transition of NADH–ubiquinone oxidoreductase (complex I) in rat heart. Biochim. Biophys. Acta *1556*, 6–12.

Maklashina, E., Kotlyar, A.B., and Cecchini, G. (2003). Active/de-active transition of respiratory complex I in bacteria, fungi, and animals. Biochim. Biophys. Acta *1606*, 95–103.

Maklashina, E., Kotlyar, A.B., Karliner, J.S., and Cecchini, G. (2004). Effect of oxygen on activation state of complex I and lack of oxaloacetate inhibition of complex II in Langendorff perfused rat heart. FEBS Lett. *556*, 64–68.

Methner, C., Chouchani, E.T., Buonincontri, G., Pell, V.R., Sawiak, S.J., Murphy, M.P., and Krieg, T. (2014). Mitochondria selective S-nitrosation by mitochondria-targeted S-nitrosothiol protects against post-infarct heart failure in mouse hearts. Eur. J. Heart Fail. *16*, 712–717.

Murphy, M.P. (2009). How mitochondria produce reactive oxygen species. Biochem. J. *417*, 1–13.

Pell, V.R., Spiroski, A.-M., Mulvey, J., Burger, N., Costa, A.S.H., Logan, A., Gruszczyk, A.V., Rosa, T., James, A.M., Frezza, C., et al. (2018). Ischemic preconditioning protects against cardiac ischemia reperfusion injury without affecting succinate accumulation or oxidation. J. Mol. Cell. Cardiol. *123*, 88–91.

Pfanner, N., Warscheid, B., and Wiedemann, N. (2019). Mitochondrial proteins: from biogenesis to functional networks. Nat. Rev. Mol. Cell Biol. *20*, 267–284.

Prime, T.A., Blaikie, F.H., Evans, C., Nadtochiy, S.M., James, A.M., Dahm, C.C., Vitturi, D.A., Patel, R.P., Hiley, C.R., Abakumova, I., et al. (2009). A mitochondria-targeted S-nitrosothiol modulates respiration, nitrosates thiols, and protects against ischemia-reperfusion injury. Proc Natl Acad Sci U S A *106*, 10764–10769.

Pryde, K.R., and Hirst, J. (2011). Superoxide is produced by the reduced flavin in mitochondrial complex I: a single, unified mechanism that applies during both forward and reverse electron transfer. J. Biol. Chem. *286*, 18056–18065.

Reichert, K., Colantuono, B., McCormack, I., Rodrigues, F., Pavlov, V., and Abid, M.R. (2017). Murine left anterior descending (LAD) coronary artery ligation: an improved and simplified model for myocardial infarction. J. Vis. Exp. *122*, e55353.

Requejo, R., Hurd, T.R., Costa, N.J., and Murphy, M.P. (2010). Cysteine residues exposed on protein surfaces are the dominant intramitochondrial thiol and may protect against oxidative damage. FEBS J. *277*, 1465–1480.

Roberts, P.G., and Hirst, J. (2012). The deactive form of respiratory complex I from mammalian mitochondria is a Na^+/H^+ antiporter. J. Biol. Chem. *287*, 34743–34751.

Rossi, C., Cremona, T., Machinist, J.M., and Singer, T.P. (1965). Studies on the respiratory chain-linked reduced nicotinamide adenine dinucleotide Dehydrogenase: VIII. Inactivation, fragmentation, and protection by substrates. J. Biol. Chem. *240*, 2634–2643.

Sharpley, M.S., Shannon, R.J., Draghi, F., and Hirst, J. (2006). Interactions between phospholipids and NADH:ubiquinone oxidoreductase (complex I) from bovine mitochondria. Biochemistry *45*, 241–248.

Shevchenko, A., Wilm, M., Vorm, O., and Mann, M. (1996). Mass spectrometric sequencing of proteins silver-stained polyacrylamide gels. Anal. Chem. *68*, 850–858.

Shevchenko, A., Tomas, H., Havli, J., Olsen, J.v., and Mann, M. (2006). In-gel digestion for mass spectrometric characterization of proteins and proteomes. Nat. Protoc. *1*, 2856–2860.

Siebels, I., and Dröse, S. (2016). Charge translocation by mitochondrial NADH:ubiquinone oxidoreductase (complex I) from Yarrowia lipolytica measured on solid-supported membranes. Biochem. Biophys. Res. Commun. *479*, 277–282.

Sievers, F., Wilm, A., Dineen, D., Gibson, T.J., Karplus, K., Li, W., Lopez, R., McWilliam, H., Remmert, M., Söding, J., et al. (2011). Fast, scalable generation of high-quality protein multiple sequence alignments using Clustal Omega. Mol. Syst. Biol. *7*, 539.

Slater, E.C. (1950). The dihydrocozymase-cytochrome c reductase activity of heartmuscle preparation. Biochem. J. *46*, 499–503.

Srere, P.A. (1969). Citrate synthase. Meth. Enzymol. *13*, 3–11.

Trujillo, J.G., Ceballos, G., Yañez, R., and Joseph-Nathan, P. (1991). Regioselective synthesis of (+)-*S*-2-amino-5-iodoacetamidopentanoic and (+)-*S*-2-amino-6-iodoacetamidohexanoic acids. Synth. Commun. *21*, 683–691.

Tyler, D.D., and Gonze, J. (1967). The preparation of heart mitochondria from laboratory animals. Methods Enzymol. *10*, 74–77.

Ushakova, A.V., Grivennikova, V.G., Ohnishi, T., and Vinogradov, A.D. (1999). Triton X-100 as a specific inhibitor of the mammalian NADH-ubiquinone oxidoreductase (complex I). Biochim. Biophys. Acta *1409*, 143–153.

Vinogradov, A.D. (1998). Catalytic properties of the mitochondrial NADH–ubiquinone oxidoreductase (complex I) and the pseudo-reversible active/inactive enzyme transition. Biochim. Biophys. Acta *1364*, 169–185.

Wiegand, G., and Remington, S.J. (1986). Citrate synthase: structure, control, and mechanism. Annu. Rev. Biophys. Biophys. Chem. *15*, 97–117.

Wilson, R.J., Drake, J.C., Cui, D., Lewellen, B.M., Fisher, C.C., Zhang, M., Kashatus, D.F., Palmer, L.A., Murphy, M.P., and Yan, Z. (2018). Mitochondrial protein S-nitrosation protects against ischemia reperfusion-induced denervation at neuromuscular junction in skeletal muscle. Free Radic. Biol. Med. *117*, 180–190.

Wittig, I., Karas, M., and Schägger, H. (2007). High resolution clear native electrophoresis for in-gel functional assays and fluorescence studies of membrane protein complexes. Mol. Cell Proteom. *6*, 1215–1225.

Wollenberger, A., Ristau, O., and Schoffa, G. (1960). Eine einfache Technik der extrem schnellen Abkühlung größerer Gewebestücke. Pflugers Arch. Gesamte Physiol. Menschen Tiere *270*, 399–412.

Yin, Z., Burger, N., Kula-Alwar, D., Aksentijević, D., Bridges, H.R., Prag, H.A., Grba, D.N., Viscomi, C., James, A.M., Mottahedin, A., et al. (2021). Structural basis for a complex I mutation that blocks pathological ROS production. Nat. Commun. *12*, 707.

Zhu, J., Vinothkumar, K.R., and Hirst, J. (2016). Structure of mammalian respiratory complex I. Nature *536*, 354–358.

STAR★METHODS

KEY RESOURCES TABLE

REAGENT or RESOURCE	SOURCE	IDENTIFIER
Chemicals, peptides, and recombinant proteins		
Alamethicin from *Trichoderma viride*	Sigma-Aldrich	Cat#A4665; CAS#27061-78-5
Amplex Red	ThermoFisher	Cat#A12222; CAS#119171-73-2
Bovine serum albumin (BSA) fatty acid free	Sigma-Aldrich	Cat#A3803; CAS#9048-46-8
Carbonyl cyanide 4-(trifluoromethoxy) phenylhydrazone (FCCP)	Sigma-Aldrich	Cat#C2920; CAS#370-86-5
Horseradish peroxidase	Sigma-Aldrich	Cat#P8250; CAS#9003-99-0
Iodoacetamide (light (L-)IAM)	Sigma-Aldrich	Cat#I1149; CAS#144-48-9
$^{13}C_2$, 2-d_2 Iodoacetamide (heavy (H-)IAM)	Sigma-Aldrich	Cat#721328; CAS#144-48-9
N-ethylmaleimide (NEM)	Sigma-Aldrich	Cat#E3876; CAS#1619234-07-9
d_5-*N*-ethylmaleimide (d_5-NEM)	Cambridge Isotope Laboratories	Cat#DLM-6711-10; CAS#36078-37-2
N-(5-fluorescein) maleimide	Sigma-Aldrich	Cat#38132; CAS#75350-46-8
Cy5 maleimide	GE Healthcare	Cat#PA25001
TPP-Iodoacetamide (TPP-IAM)	This manuscript	N/A
d_{15}-TPP-Iodoacetamide (d_{15}-TPP-IAM)	This manuscript	N/A
S-Methyl methanethiosulfonate (MMTS)	Sigma-Aldrich	Cat#64306; CAS#2949-92-0
Rotenone	Santa Cruz Biotechnology	Cat#sc-203242; CAS#83-79-4
Piericidin A	Stratech	Cat#3535-APE; CAS#2738-64-9
Superoxide dismutase from bovine liver	Sigma-Aldrich	Cat#S8160; CAS#9054-89-1
Precision Plus Protein™ Dual Color Standard	Bio-Rad	Cat#161-0374
NADH reduced disodium salt hydrate	Sigma-Aldrich	Cat#N8129; CAS#606-68-8
NADPH reduced tetra sodium salt hydrate	Sigma-Aldrich	Cat#N7505; CAS#2646-71-1
Decylubiquinone	Sigma-Aldrich	Cat#D7911; CAS#55486-00-5
Potassium cyanide	Fluka	Cat#60179; CAS#151-50-8
Antimycin A	Sigma-Aldrich	Cat#A8674; CAS#1397-94-0
Cytochrome *c* from equine heart	Sigma-Aldrich	Cat#C2506; CAS#9007-43-6
L-Glutathione reduced (GSH)	Sigma-Aldrich	Cat#G4251; CAS#70-18-8
Glutamate	Sigma-Aldrich	Cat#G1251; CAS#56-86-0
Malate	Sigma-Aldrich	Cat#112577; CAS#97-67-6
Succinate	Sigma-Aldrich	Cat#S3674; CAS#110-15-6
ADP monopotassium salt	Sigma-Aldrich	Cat#A5285; CAS#72696-48-1
Pierce™ TCEP-HCl	ThermoFisher	Cat#20490; CAS#51805-45-9
DTNB (5,5-dithio-bis-(2-nitrobenzoic acid)	Sigma-Aldrich	Cat#D8130; CAS#69-78-3
Acetyl-CoA sodium salt	Sigma-Aldrich	Cat#A2056; CAS#102029-73-2
Oxaloacetate	Sigma-Aldrich	Cat#O4126; CAS#328-42-7
QC Colloidal Coomassie Stain	Bio-Rad	Cat#161-08-03
Triton™ X-100	ThermoFisher	Cat#BP151-500; CAS#9002-93-1
n-Dodecyl D β-maltoside (DDM)	Sigma-Aldrich	Cat#D4641; CAS#69227-93-6
Digitonin	Sigma-Aldrich	Cat#D141; CAS#11024-24-1
Sodium dodecyl sulfate	Sigma-Aldrich	Cat#L3771; CAS#151-21-3
Trypsin Sequencing Grade	Roche	Cat#11418475001
Dithiothreitol (DTT)	Sigma-Aldrich	Cat#D0632; CAS#3483-12-3
Ammonium bicarbonate	Fluka	Cat#40867-50G; CAS#1066-33-7
Potassium dihydrogen orthophosphate	Fisher Scientific	Cat#10783611; CAS#7778-77-0

(*Continued on next page*)

Continued

REAGENT or RESOURCE	SOURCE	IDENTIFIER
Potassium Chloride	Fisher Scientific	Cat#10375810; CAS#7447-40-7
HEPES	Sigma-Aldrich	Cat#H3375; CAS#7365-45-9
Trizma Base (Tris-(hydroxymethyl)-aminomethan)	Sigma-Aldrich	Cat#93350; CAS#77-86-1
Sucrose	Sigma-Aldrich	Cat#S0389; CAS#57-50-1
Fmoc-Arg(Pbf)-Wang resin	Sigma-Aldrich	Cat# 47362-1G
Fmoc-Ala-OH-2,3,3,3-d_4	Anaspec inc.	CAS# 225101-69-9
Fmoc-L-Ala-OH*H2O	Iris-Biotech (Germany)	Cat# FAA1000; CAS# 79990-15-1
Fmoc-L-Asn(Trt)-OH	Iris-Biotech (Germany)	Cat# FAA1015; CAS# 132388-59-1
Fmoc-L-Pro-OH*H2O	Iris-Biotech (Germany)	Cat# FAA1185; CAS# 71989-31-6
Fmoc-Tyr-OH	Iris-Biotech (Germany)	Cat# FAA1230; CAS# 71989-38-3
Fmoc-Glu(tBu)-OH	Iris-Biotech (Germany)	Cat# FAA1045; CAS# 71989-18-9
Fmoc-Cys(Trt)-OH	Iris-Biotech (Germany)	Cat# FAA1040; CAS# 103213-32-7
Fmoc-Gly-OH	Iris-Biotech (Germany)	Cat# FAA1050; CAS# 29022-11-5
Fmoc-Phe-OH	Iris-Biotech (Germany)	Cat# FAA1175; CAS# 35661-40-6
Fmoc-Asp(tBu)-OH	Iris-Biotech (Germany)	Cat# FAA1020; CAS# 71989-14-5
Fmoc-Thr(tBu)-OH	Iris-Biotech (Germany)	Cat# FAA1210; CAS# 71989-35-0
Fmoc-Ser(tBu)-OH	Iris-Biotech (Germany)	Cat# FAA1190; CAS# 71989-33-8
Dimethylformamide (DMF) peptide synthesis grade	Sigma-Aldrich	Cat# 1.00397; CAS# 68-12-2
piperidine	Iris-Biotech (Germany)	Cat# SOL-010; CAS# 110-89-4
Ethyl (hydroxyimino)cyanoacetate	Sigma-Aldrich	Cat# 233412-50G; CAS# 3849-21-6
N,N'-Diisopropylcarbodiimide (DIC)	Iris-Biotech (Germany)	Cat# RL-1015; CAS# 693-13-0
Trifluoroacetic acid	Iris-Biotech (Germany)	Cat# SOL-011; CAS# 76-05-1
Triisopropylsilane (TIS)	Iris-Biotech (Germany)	Cat# RL-1102; CAS# 6485-79-6
2,2′-(Ethylenedioxy)diethanethiol (DODT)	Sigma-Aldrich	Cat# 465178; CAS# 14970-87-7
diethyl ether	Sigma-Aldrich	Cat# 296082; CAS# 60-29-7
dichloromethane	Sigma-Aldrich	Cat# 34856; CAS# 75-09-2
Critical commercial assays		
Pierce™ BCA Protein Assay Kit	ThermoFisher	Cat#23225
Deposited data		
Raw NMR data and transformed spectra for TPP-Iodoacetamide and d_{15}-TPP-Iodoacetamide	This manuscript	https://doi.org/10.5525/gla.researchdata.1143
Experimental models: Organisms/strains		
C57BL/6J mice	Charles River	Cat#632
Female Wistar rats	Charles River	Cat#003
Software and algorithms		
GraphPad Prism 9	GraphPad Software	https://www.graphpad.com
ImageJ	NIH	https://imagej.nih.gov/ij/
MassLynx 4.1	Waters	https://www.waters.com/waters/en_US/MassLynx-MS-Software/nav.htm?locale=en_US&cid=513662
MaxQuant (v1.6.10.43 and v1.6.17.1)	Max Planck Institute of Biochemistry	https://maxquant.org
Thermo Xcalibur software	Thermo Fisher Scientific	https://www.thermofisher.com/order/catalog/product/OPTON-30965#/OPTON-30965
Thermo Proteome Discoverer (v1.4)	Thermo Fisher Scientific	https://www.thermofisher.com/us/en/home/industrial/mass-spectrometry/liquid-chromatography-mass-spectrometry-lc-ms/lc-ms-software/multi-omics-data-analysis/proteome-discoverer-software.html

(*Continued on next page*)

Continued

REAGENT or RESOURCE	SOURCE	IDENTIFIER
Other		
Sodium pentobarbital solution (Euthatal)	Merial Animal Health	N/A
MINIVENT Mouse Ventilator	Hugo Sachs Elektronik Harvard Apparatus	N/A
7-0 Prolene suture (TF-6)	Ethicon	N/A
Fine Bore Polyethene Tubing (used as snare)	Portex	N/A
ACQUITY UPLC® BEH C18 MS Column (1.7 µm, 130 Å, 50 × 1 mm)	Waters	Cat#186002344
Precellys24 tissue homogeniser	Bertin Instruments	N/A
Precellys CK14 tissue lysis tubes	Bertin Instruments	P000973-LYSK0-A.0
Fisherbrand™ Pre-Filled Bead Mill Tubes 1.4 mm ceramic beads	Fisher Scientific	15-340-153
OMIX C18 tips	Agilent	Cat#A57003100
Eppendorf Protein LoBind tubes 1.5 ml	Eppendorf	Cat#022431081
ACQUITY UPLC® I-Class	Waters	N/A
Xevo TQ-S mass spectrometer	Waters	N/A
Acclaim PepMap C18 reversed-phase column (2 µm, 100 Å, 50 µM × 150 mm)	Thermo Fisher Scientific	164562
Proxeon EASY- nLC 1000 system	Thermo Fisher Scientific	N/A
Q-Exactive Plus mass spectrometer	Thermo Fisher Scientific	N/A
Mini-PROTEAN® TGX Protein Gels 12%	Bio-Rad	Cat#456-1044
Micro Bio-Spin 6 columns	Bio-Rad	Cat#732-6221
NativePAGE™ 3 to 12% Bis-Tris	Thermo Fisher Scientific	Cat#BN1001BOX
ClarioSTAR Plus	BMG Labtech	N/A
SPECTRAmax Plus 384 plate reader	Molecular Device	N/A
Amersham Typhoon RGB Biomolecular Imager	GE Lifescience	N/A
Liberty Blue peptide synthesizer	CEM, UK	N/A
Varian 940-LC	Varian inc.	N/A
Luna C18 column (250 × 10 mm, 10 µm)	Phenomenex	00G-4253-N0

RESOURCE AVAILABILITY

Lead contact

Further information and requests for resources and reagents should be directed to and will be fulfilled by the Lead Contact, Michael P. Murphy (mpm@mrc-mbu.cam.ac.uk).

Materials availability

This study generated the following unique reagents:

TPP-IAM and d_{15}-TPP-IAM

ND3 and d_8-ND3 peptides (labelled with light or heavy ($^{13}C_2$, 2-d_2) iodoacetamide).

These reagents are available from the lead contact under Materials Transfer Agreements.

Data and code availability

Original source data for Figures 2, 3, 4, 5, 6, and S1–S6 is available from the corresponding author upon reasonable request. Raw NMR data for synthesized compounds together with transformed spectra are available at https://doi.org/10.5525/gla.researchdata.1143. This paper does not report original code. Any additional information required to analyze the data reported in this paper is available from the lead contact upon request.

EXPERIMENTAL MODEL AND SUBJECT DETAILS

All procedures were carried out in accordance with the UK Animals (Scientific Procedures) Act 1986 and the University of Cambridge Animal Welfare Policy. Procedures were approved to be carried out under the Project Licenses: 70/7963, 70/8238. Female Wistar rats, or male or female C57BL/6J mice (both Charles River Laboratories, UK) were maintained in pathogen-free facilities with *ad libitum* chow and water until being 8–20 weeks of age for experimental use.

METHOD DETAILS

Synthesis of labelled ND3 peptides

ND3 and d_8-ND3 (containing two fully deuterated alanine residues) with the sequence ANPYEC(carbamidomethyl)GFDPTSSAR were synthesized on a Liberty Blue peptide synthesizer using microwave irradiation on Fmoc-Arg(Pbf)-Wang resin using Fmoc chemistry with five equivalents of Fmoc-amino acids, N,N′-diisopropyl-carbodiimide (DIC) and ethyl (hydroxyimino)cyanoacetate (Oxyma) in a 1: 1:1 ratio in dimethylformamide (DMF). Fmoc-Cys(Trt)-OH was coupled at 50°C, other amino acids were coupled at 75°C for 10 min. The resin was washed with DCM and diethylether (3 × 30 seconds each), and dried *in vacuo*. Peptide was deprotected and cleaved off the resin using 5 ml of TFA/DODT/TIS/H_2O (94:2.5:1:2.5) for 90 min. Purification of peptides was performed on a Varian 940-LC equipped with a Phenomenex Luna C18 column (250 × 10 mm, 10 μm) using a flow rate of 4 ml min^{-1} and a gradient of 0–100% B (3 min 100% A (99.9% H_2O, 0.1% TFA), then to 100% B (99.9% ACN, 0.1% TFA) in 22 min). Peptides (500 μl, 3.3 mM, 1.67 μmol) were alkylated with iodoacetamide or $^{13}C_2$, 2-d_2 iodoacetamide (3 μmol, 0.57 mg) in pH 7 for 1 hour until completion. Alkylated peptides were purified by HPLC as described above.

Synthesis of TPP-IAM and d_{15}-TPP-IAM

TPP-IAM and d_{15}-TPP-IAM were synthesized from 5-amino-1-pentanol **1** in three steps (Figure S1B). 5-amino-1-pentanol **1** was first converted into 5-iodopent-1-ylammonium iodide salt **2** with hydrogen iodide, then the iodide was displaced by triphenylphosphine or d_{15}-triphenylphosphine to give the aminopentyl-TPP derivative **3** or aminopentyl-d_{15}-TPP derivative **4**. These were then iodoacetylated using 4-nitrophenyl iodoacetate (prepared from iodoacetic acid and 4-nitrophenol) to give TPP-IAM and d_{15}-TPP-IAM.

5-Iodopent-1-ylammonium iodide. A solution of 5-amino-1-pentanol (3.00 g, 29.1 mmol, 1.00 eq) in hydroiodic acid (aqueous, 55%, 11.0 mL, 3.00 eq) was allowed to stir for 16 h at 100°C then concentrated under reduced pressure. Trituration from diethyl ether followed by recrystallization from acetone gave the title compound as large colorless crystals (4.27 g, 43%). δ_H (400 MHz, CD_3OD): 1.46–1.54 (2H, m, CH_2, H-3), 1.64–1.72 (2H, m, CH_2, H-2), 1.82–1.88 (2H, m, CH_2, H-4), 2.94 (2H, t, *J* = 7.6 Hz, CH_2N), 3.25 (2H, t, *J* = 6.8 Hz, CH_2I); δ_C (101 MHz, CD_3OD): 4.9 (CH_2, C-5), 26.1 (CH_2, C-4), 26.9 (CH_2, C-3), 32.6 (CH_2, C-2), 32.9 (CH_2, C-1); HRMS (ESI$^+$, m/z): found $[M + H]^+$ 214.0085. $C_5H_{13}IN^+$ requires 214.0087. ν_{max} (CD_3OD): 2933 (CH), 2991 (CH), 3345 (NH). MP: 137–139°C.

(5-Aminopent-1-yl)triphenylphosphonium iodide, hydroiodide salt. A solution of 5-iodopent-1-ylamine hydroiodide (1.50 g, 4.40 mmol, 1.00 eq) and triphenylphosphine (2.31 g, 8.80 mmol, 2.00 eq) in anhydrous acetonitrile (60.0 mL) in a flame-dried flask under argon was allowed to stir at 85°C for 72 h then concentrated under reduced pressure. Trituration from diethyl ether gave the title compound as a colorless hygroscopic powder (2.60 g, 98%). δ_H (400 MHz, CD_3OD): 1.55–1.95 (6H, m, 3 × CH_2, H-3, H-4, H-2), 2.85–3.11 (2H, m, CH_2N), 3.50–3.68 (2H, m, CH_2P), 7.60–7.98 (15H, m, Ph_3P); δ_C (101 MHz, CD_3OD): 21.6 (d, *J* = 46.1 Hz, CH_2, C-1), 21.8 (CH_2, C-4), 26.5 (d, *J* = 1.8 Hz, CH_2, C-3), 27.0 (d, *J* = 17.3 Hz, CH_2, C-2), 39.0 (CH_2, C-5), 118.4 (d, *J* = 86.7 Hz, 3 × C, P-C PPh_3), 130.2 (d, *J* = 12.7 Hz, 6 × CH, *o*-C PPh_3), 133.6 (d, *J* = 9.8 Hz, 6 × CH, *m*-C PPh_3), 134.9 (d, *J* = 3.0 Hz, 3 × CH, *p*-C PPh_3); δ_P (67 MHz, CD_3OD): 23.5; HRMS (ESI$^+$, m/z): found 348.1860. $C_{23}H_{27}NP^+$ requires 348.1876. n_{max} (CD_3OD): 1435 (PPh), 2881 (CH), 2985 (CH), 3363 (NH).

(5-Aminopent-1-yl)tri(pentadeuterophenyl)phosphonium iodide, hydroiodide salt. A solution of 5-iodopent-1-ylamine hydroiodide (200 mg, 0.587 mmol, 1.00 eq) and d_{15}-triphenylphosphine (326 mg, 1.17 mmol, 2.00 eq) in anhydrous acetonitrile (20.0 mL) in a flame-dried flask under argon was allowed to stir under argon at 85°C for 72 h then concentrated under reduced pressure. Trituration from diethyl ether gave the title compound as a fine colorless hygroscopic powder (336 mg, 93%). δ_H (400 MHz, CD_3OD): 1.56–1.90 (6H, m, 3 × CH_2, H-3, H-4, H-2), 2.86–2.99 (2H, m, CH_2, H-5), 3.46–3.58 (2H, m, CH_2, H-1); δ_C (101 MHz, CD_3OD): 21.5 (d, *J* = 48.1 Hz, CH_2, C-1), 21.8 (CH_2, C-4), 26.5 (d, *J* = 1.7 Hz, CH_2, C-3), 27.0 (d, *J* = 17.4 Hz, CH_2, C-2), 39.0 (CH_2, C-5), 118.2 [d, *J* = 85.9 Hz, 3 × C, P-C $P(C_6D_5)$], 129.7 [td, *J* = 25.4, 12.7 Hz, 6 × CD, *o*-C $P(C_6D_5)$], 133.1 [td, *J* = 24.4, 9.3 Hz, 6 × CD, *m*-C $P(C_6D_5)$], 134.4 [td, *J* = 23.9, 2.9 Hz, 3 × CD, *p*-C $P(C_6D_5)$]; δ_P (67 MHz, CD_3OD): 24.2; HRMS (ESI$^+$, m/z): found 363.2814. $C_{23}H_{12}D_{15}NP^+$ requires 363.2817. ν_{max} (CD_3OD): 2881 (CH), 2981 (CH), 3444 (NH).

4-Nitrophenyl iodoacetate; To a solution of 4-nitrophenol (1.79 g, 12.9 mmol, 1.20 eq) in anhydrous dichloromethane (120 mL) in a flame-dried flask under argon at 0°C was added iodoacetic acid (2.00 g, 10.8 mmol, 1.00 eq) and *N,N′*-dicyclohexylcarbodiimide (2.88 g, 14.0 mmol, 1.30 eq). The reaction mixture was allowed to stir under argon at 0°C for 1 h then for 16 h at room temperature. The solid was filtered and discarded and the filtrate concentrated under reduced pressure. The resulting residue was dissolved in toluene, filtered and concentrated. Recrystallisation from ethanol gave the title compound as fine colorless crystals (0.696 g, 21%). δ_H (400 MHz, $CDCl_3$): 3.93 (2H, s, CH_2), 7.31 (d, 2H, *J* = 9.1 Hz, 2 × CH, H-2), 8.29 (d, 2H, *J* = 9.1 Hz, 2 × CH, H-3); δ_C (101 MHz, $CDCl_3$): −6.7 (CH_2), 122.0 (2 × CH, C-2), 125.4 (2 × CH, C-3), 145.7 (C-1), 155.2 (C-4), 166.7 (C); HRMS (ESI$^+$, m/z): found $[M + Na]^+$ 329.9234. $C_8H_6INNaO_4^+$ requires 329.9234. Spectral data agree with the literature (Trujillo et al., 1991).

[5-(2′-iodoacetylamino)pent-1-yl]triphenylphosphonium iodide; To a solution of (5-aminopent-1-yl)triphenylphosphonium iodide hydroiodide (1.29 g, 2.13 mmol, 1.00 eq) and triethylamine (0.13 mL, 2.13 mmol, 1.00 eq) in anhydrous dichloromethane (100 mL) at −78°C in a flame-dried flask under argon was added 4-nitrophenyl iodoacetate (654 mg, 2.13 mmol, 1.00 eq). The reaction mixture was allowed to stir under argon at −78°C for 20 min then concentrated under reduced pressure. The resulting solid was partitioned between dichloromethane and water and the extracts separated. The organic layer was washed with water, dried over anhydrous magnesium sulfate and concentrated under reduced pressure. Column chromatography [SiO_2, dichloromethane: methanol (100:0–90:10)] followed by trituration from diethyl ether gave the title compound as a fine off-white hygroscopic powder (691 mg, 50%). δ_H (400 MHz, CD_3OD): 1.50–1.77 (6H, m, 3 × CH_2, H-3, H-4, H-2), 3.14 (2H, t, *J* = 6.3 Hz, CH_2N), 3.38–3.46 (2H, m, CH_2P), 3.66

(2H, s, CH_2, H-2′), 7.71–7.94 (15H, m, Ph_3P); δ_C (101 MHz, CD_3OD): −2.5 (CH_2, C-2′), 21.5 (d, J = 54.6 Hz, CH_2, C-1), 21.7 (CH_2, C-3), 27.2 (d, J = 17.1 Hz, CH_2, C-2), 27.8 (CH_2, C-4), 38.8 (CH_2, C-5), 118.5 (d, J = 85.0 Hz, 3 × C, P-C PPh_3), 130.2 (d, J = 12.9 Hz, 6 × CH, *o*-C PPh_3), 133.5 (d, J = 9.8 Hz, 6 × CH, *m*-C PPh_3), 134.9 (d, J = 3.0 Hz, 3 × CH, *p*-C PPh_3), 169.9 (C-1′); δ_P (67 MHz, CD_3OD): 23.8; HRMS (ESI^+, m/z): found $[M]^+$ 516.0950. $C_{25}H_{28}INOP^+$ requires 516.0948. ν_{max} (CD_3OD): 1435 (PPh), 1653 (C=O), 2868 (CH), 2922 (CH), 3444 (NH).

[5-(2′-iodoacetylamino)pent-1-yl]tri(pentadeuterophenyl)phosphonium iodide; To a solution of (5-aminopentyl)tri(pentadeuterophenyl)phosphonium iodide hydroiodide (150 mg, 0.240 mmol, 1.00 eq) and triethylamine (0.500 mL, 0.240 mmol, 1.00 eq) in anhydrous dichloromethane (10.0 mL) at −78°C in a flame-dried flask under argon was added 4-nitrophenyl iodoacetate (90 mg, 0.0290 mmol, 1.20 eq) and the reaction mixture was allowed to stir for 40 min at −78°C then warmed to room temperature and diluted with dichloromethane. The solution was washed with water and the combined organic extracts were dried over anhydrous magnesium sulfate and concentrated under reduced pressure. Column chromatography [SiO_2, dichloromethane: methanol (100:0–90:10)] followed by trituration from chloroform/diethyl ether gave the title compound as a pale-yellow hygroscopic powder (80 mg, 51%). δ_H (400 MHz, CD_3OD): 1.51–1.77 (6H, m, 3 × CH_2, H-3, H-4, H-2), 3.14 (2H, t, J = 6.6 Hz, CH_2N), 3.44–3.51 (2H, m, CH_2P), 3.68 (2H, s, CH_2, H-2′); δ_C (101 MHz, CD_3OD): −2.6 (CH_2, C-2′), 21.5 (d, J = 47.6 Hz, CH_2, C-1), 21.7 (CH_2, C-3), 27.2 (d, J = 16.2 Hz, CH_2, C-2), 27.8 (CH_2, C-4), 38.9 (CH_2, C-5), 118.3 [d, J = 87.4 Hz, 3 × C, P-C $P(C_6D_5)_3$], 129.7 [(td, J = 25.7, 12.2 Hz, 6 × CD, *o*-C $P(C_6D_5)_3$], 133.1 [td, J = 25.1, 10.2 Hz, 6 × CD, *m*-C $P(C_6D_5)_3$], 134.4 [td, J = 23.1, 2.7 Hz, 3 × CD, *p*-C $P(C_6D_5)$], 170.4 (C-1′); δ_P (67 MHz, CD_3OD): 24.2; HRMS (ESI^+, m/z): found $[M]^+$ 531.1882. $C_{25}H_{13}D_{15}INOP^+$ requires 531.1889. ν_{max} (CD_3OD): 1655 (C=O), 2874 (CH), 2956 (CH), 3454 (NH).

Clamp freezing of tissue samples

In order to preserve the metabolic *in vivo* state of tissues for subsequent analysis, retrieved tissue samples were rapidly frozen with a Wollenberger clamp (Wollenberger et al., 1960). The metal clamp was pre-cooled in liquid nitrogen until the boiling ceased (~10 min) and the clamp had reached liquid nitrogen temperature. Tissue sections were immediately clamped after excision, which increases the surface area of the tissue and results in almost instantaneous freezing.

In situ heart ischemia model

For retrieval of tissues, male or female mice, aged 8–22 weeks were culled by cervical dislocation. The heart was retrieved after sternal thoracotomy and non-ischemic tissue was immediate frozen using a Wollenberger clamp (Wollenberger et al., 1960) cooled in liquid nitrogen and subsequently stored at −70°C. To induce ischemia, excised hearts were kept in the thorax of the warmed mouse (37°C core temperature) for indicated times and then clamp frozen and stored as stated above.

LAD occlusion myocardial infarct model

In the well-established left anterior descending coronary artery (LAD) occlusion myocardial infarction model the left anterior descending (LAD) coronary artery is temporarily ligated and then reopened (Antonucci et al., 2019; Kohlhauer et al., 2019; Pell et al., 2018; Reichert et al., 2017). Mice (8–10 weeks of age; C57BL/6J; Charles River Laboratories, UK) were anesthetized with sodium pentobarbital (70 mg/kg body weight, intraperitoneally) and the depth of anesthesia was monitored via the pedal reflex to administer additional anesthesia as required. Mice were intubated endotracheally and ventilated with 3 cm H_2O positive end-expiratory pressure. Ventilation frequency was maintained at 110 breaths per min with a tidal volume of 125–150 μl. A left side lateral thoracotomy was performed with the pericardium being stripped to expose the heart. The LAD was surrounded by a 7-0 Prolene suture, with both ends passed through a small plastic tube to create a snare. The blood flow through the LAD was occluded by tightening the snare, inducing ischemia for 30 min in the left anterior ventricle wall. Then, the suture was released to reperfuse the tissue for either 5 min or 2 hrs. The left ventricle was excised and collected rapidly at different stages during the procedure (the normoxic control samples were excised before occlusion of the LAD), immediately clamp frozen and stored at −70°C.

Ex vivo brain ischemia model

Mice, at the age of 8–10 weeks, were sacrificed by cervical dislocation followed by decapitation. For the normoxic control, the whole head was immediately frozen in liquid nitrogen (within 5–8 s following cervical dislocation). Ischemic samples were produced by incubating the whole heads at 37°C for indicated time periods to induce global ischemia in the brain. At the end of ischemic period, the brains were rapidly dissected out and clamp-frozen at liquid nitrogen temperature and stored at −70°C until use.

Preparation of mitochondrial membranes

Bovine heart mitochondrial membranes (BHMMs) were kindly provided by Dr Hiran A. Prag and Prof Judy Hirst's lab (MRC Mitochondrial Biology Unit, University of Cambridge, UK) and were isolated as described previously (Sharpley et al., 2006), with all steps being performed at 4°C in a cold room. Bovine heart mitochondria were prepared by differential centrifugation in 250 mM sucrose, 10 mM Tris-Cl, 0.2 mM EDTA (pH 7.8 at 4°C) and stored at −80°C. To isolate mitochondrial membranes, ~5 g of frozen bovine heart mitochondria were thawed on ice and quickly (5 s) blended in 200 ml of Milli- Q water in a Waring blender. KCl was added to a final concentration of 150 mM before blending the suspension again (10 s) until homogeneous. Mitochondrial membranes were pelleted by centrifugation (13,500 × *g*, 40 min, 4°C) and resuspended in homogenization buffer (20 mM Tris-HCl, 1 mM EDTA, 10% v/v glycerol, pH 7.55 at 4°C). The suspension was homogenized in a Potter-Elvehjem tissue grinder with 3 gentle strokes using a PTFE pestle. The

CellPress
OPEN ACCESS

protein concentration of the membranes was determined using a BCA assay kit with BSA as a standard, prior to aliquoting (at approx. 5 mg/ml) and snap freezing in liquid nitrogen followed by storage at −70°C.

Mouse heart mitochondrial membranes (MHMMs) were kindly provided by Injae Chung, Prof Judy Hirst's lab (MRC Mitochondrial Biology Unit, University of Cambridge, UK). Mouse hearts were excised and immersed immediately in ice-cold buffer containing 10 mM Tris-HCl (pH 7.4 at 4°C), 75 mM sucrose, 225 mM sorbitol, 1 mM EGTA and 0.1% (w/v) fatty acid-free bovine serum albumin (BSA), supplemented with one cOmplete™ EDTA-free protease inhibitor tablet (Roche) per 50 ml buffer. Mitochondria were prepared as described previously (Fernández-Vizarra et al., 2010; Agip et al., 2018), with all steps carried out at 4°C. The hearts were sliced finely, washed, resuspended in 10 ml buffer per gram of tissue, and homogenized with a Potter–Elvehjem homogenizer fitted with a Teflon pestle (seven to ten strokes at 1000 rpm). The homogenate was centrifuged (1000 × *g*, 10 min), then the supernatant was recentrifuged (9000 × *g*, 10 min) to collect crude mitochondria. The pellets were suspended in resuspension buffer (20 mM Tris-HCl (pH 7.4 at 4°C), 1 mM EDTA, 10% glycerol) to a protein concentration of ~10 mg/ml and stored at −80°C. Mitochondria suspensions were thawed on ice, diluted to 5 mg/ml, and sonicated using a Q700 Sonicator (Qsonica; 65% amplitude and three 5-s bursts of sonication interspersed by 30-s intervals on ice) and then centrifuged at 75,000 × *g* for 1 hr. The pellets containing the mitochondrial membranes were homogenized in resuspension buffer to approx. 5 mg/ml and stored at −80°C.

Mitochondrial isolations

Crude rat heart mitochondria were isolated by differential centrifugation based on a previously described method (Tyler and Gonze, 1967), with all steps being performed at 4°C using pre-cooled equipment. Rats were culled by stunning followed by cervical dislocation. Tissues were excised and immediately stored in ice-cold STEB buffer (250 mM sucrose, 10 mM Tris-HCl, 1 mM EGTA, 0.1% w/v fatty acid-free BSA, pH 7.4 at 4°C). Residual aorta, connective and fat tissue were removed and the heart was sliced into pieces and rinsed thoroughly in ice-cold STEB. The tissues pieces were chopped finely with a razor blade rinsed thoroughly with STEB and homogenized in a Potter-Elvehjem tissue grinder using first a loose-fitting PTFE pestle during 7–10 strokes followed by 10 strokes with a tight-fitting PTFE pestle. The homogenate was centrifuged twice (700 × *g*, 5 min, 4°C) and the filtered through a double layer of pre-wetted muslin. Crude mitochondria were pelleted by centrifugation (10,000 × *g*, 5 min, 4°C), washed once with STEB and centrifuged again. Mitochondria were resuspended in STE buffer (without BSA) and the protein concentration was determined using a BCA assay kit with BSA as standard. Crude mitochondria were stored on ice and used within 2 hrs.

Mitochondrial membrane incubations

Isolated mitochondrial membranes were incubated in KPi buffer (50 mM KPi, pH 7.8 at 30°C), shaking at 37°C for 20 (BHMMs) or 30 (MHMMs) min, in order to deplete substrates and thereby deactivate complex I (thermal deactivation; (Babot et al., 2014; Kotlyar and Vinogradov, 1990)). Active samples were prepared by incubating deactivated samples, or alternatively samples that were kept on ice, in the presence of NADH (1 mM) for 5 min on ice. Subsequently, membranes were pelleted (17,000 × *g*, 3 min, 4°C) and thiols were labelled if specified.

For the comparison of nicotinamide adenine dinucleotides, BHMMs were incubated in presence of 1 mM NADH or NADPH for 30 min at 23°C. Then, membranes were pelleted (17,000 × *g*, 3 min, 4°C) and thiols were differentially labelled.

Labelling of membranes for activity assays

First, mitochondrial membranes were pre-incubated (see *mitochondrial membrane incubations*) and pelleted (17,000 × *g*, 3 min, 4°C). The pellet was resuspended in ice-cold KPi buffer (50 mM KPi, pH 7.8 at 30°C) containing IAM, MMTS, NEM or TPP-IAM at indicated concentrations and samples were labelled for 5 min on ice (TPP-IAM labelling was performed at RT with 0.1 mM NADH present in active samples during the labelling; unlabelled controls were resuspended in KPi buffer). Samples were pelleted (17,000 × *g*, 3 min, 4°C), resuspended in cold KPi buffer (6 μg protein/100 μl) and NADH/dQ oxidoreductase activity was measured.

Complex I activity assay

NADH/dQ oxidoreductase

Complex I activity was determined by measuring the rotenone-sensitive rate of NADH oxidation in the presence of decylubiquinone (dQ) as electron acceptor in presence of antimycin A and KCN to block downstream electron flow through the respiratory chain. NADH absorbance was measured with a two-wavelength UV-Vis microplate reader (SPECTRAmax Plus 384 plate reader (Molecular Devices, UK)). The assay was performed in 96-well plates with a total assay volume of 200 μl per well. First, 50 μl of assay buffer (final assay concentration: 0.2 mM KCN, 0.3 μM antimycin A, 100 μM decylubiquinone and ethanol or 0.5 μM rotenone in KPi buffer (50 mM KPi, pH 7.8 at 30°C)) were distributed into the wells and kept on ice. Next, 100 μl of sample solution (as specified) were added and the assay was started with 50 μl of NADH (0.2 mM final). The NADH oxidation was measured for 30 min at 30°C monitoring the absorbance at λ = 340 and 380 nm in 8–12 second intervals. The maximum linear rate of NADH oxidation was calculated by subtracting the absorbance at 340–380 nm of at least duplicate samples, followed by the subtraction of the rotenone-insensitive background oxidation rate, measured in samples with rotenone. The NADH concentration was determined using the extinction coefficient $\varepsilon_{340\text{-}380}$ = 4.81 $mM^{-1}cm^{-1}$ (Fedor et al., 2017; Sharpley et al., 2006).

NADH/O_2 oxidoreductase (combined labelling)

BHMMs (2 μg protein/well in 100 μl KPi buffer (50 mM KPi, pH 7.8 at 30°C)) were added to 25 μl of turnover buffer (KPi buffer containing 1.5 μM Cyt *c* and 15 μg/ml alamethicin (final for 200 μl volume)) and prewarmed at 32°C for 1.5 min in a two-wavelength UV-Vis

microplate reader (SPECTRAmax Plus 384 plate reader (Molecular Devices, UK)). The assay was started by addition of 50 μl NADH (0.8 mM stock) and NADH oxidation was determined by monitoring the absorbance at λ = 340 and 380 nm in 12 second intervals at 32°C shaking the plate for 2 s before each measurement. After 2 min 5 μl of iodoacetamide (180 mM/5 mM final) or KPi buffer for control was added to indicated samples (Set 1) and measurements were continued for 8 min. The labelling reaction was quenched by addition of 20 μl GSH (200 mM, in KPi buffer additionally pH-ed with KOH to pH 7.4, 20 mM final) and measurements were continued. Once NADH was depleted, the measurement was continued for 20 min to deactivate complex I and 5 μl of iodoacetamide (180 mM/5 mM final) or KPi buffer for control was added to indicated samples (Set 2). Samples were labelled for 8 min and the labelling was quenched by addition of 20 μl GSH (200 mM, in KPi buffer additionally pH-ed with KOH to pH 7.4, 20 mM final). Then, 10.52 μl of NADH (4 mM stock) was added and NADH oxidation was monitored for 30 min. Complex I independent NADH oxidation was monitored in equally treated wells containing 2 μM piericidin A. The NADH concentration was determined using the extinction coefficient $\varepsilon_{340\text{-}380}$ = 4.81 $mM^{-1}cm^{-1}$ (Fedor et al., 2017; Sharpley et al., 2006).

Tissue homogenisation for activity assays

To prepare tissue homogenate for enzyme assays, ~5 mg of tissue was weighed into lysis tubes (Precellys, CK14, Bertin Instruments, France or 1.4 mm ceramic bead filled tubes, Fisher Scientific, USA) that were pre-cooled on dry ice. In the following steps only two samples were processed at a time to allow rapid handling. Ice-cold KPi buffer (50 mM KPi, pH 7.8 at 30°C) (400 μl) was added, and tissue was rapidly homogenized in the tissue homogenizer (Precellys 24, Bertin Instruments, France) at 6500 rpm for 15 s. The homogenate was transferred into fresh Eppendorf tubes on ice, and aliquoted/snap-frozen (20 μl) into 0.5 ml Eppendorf tubes, which were pre-cooled on dry ice. The protein concentration of the homogenate was determined after freezing using a BCA assay kit with BSA as a standard. The homogenates were stored at −70°C.

Complex I activity in tissue homogenate

To assess complex I activity, tissue homogenate (preparation described above) was thawed on ice directly before performing the assay and diluted in KPi buffer (50 mM KPi, pH 7.8 at 30°C) containing 0.05% DDM (0.025% final, if not otherwise specified) to a protein concentration of 10 μg protein/100 μl that was added to each well. The NADH/dQ oxidoreductase activity of the diluted tissue homogenate was measured as described above.

Citrate synthase activity assay

Citrate synthase activity was determined by measurement of the citrate synthase-catalyzed formation of citrate from acetyl-CoA and oxaloacetate by detecting the amount of produced CoA with DTNB (Ellman's reagent, 5,5-dithio-bis-(2-nitrobenzoic acid)) (Srere, 1969; Wiegand and Remington, 1986). The free thiol of CoA reacts with DTNB to form a mixed disulfide and the yellow colored TNB^{2-} (2-nitro-5-thiobenzoic acid), which is quantified by measuring its absorbance at λ = 412 nm (Ellman, 1959; Srere, 1969). Tissue homogenate was thawed on ice and diluted in KPi buffer (50 mM KPi, pH 7.8 at 30°C) (containing DDM if indicated) to a concentration 10 μg protein/100 μl that was added to each well. The KPi reaction buffer (80 μl/well), containing DTNB (100 μM) and acetyl-CoA (300 μM) (both final assay concentrations) was pre-plated and 100 μl of diluted tissue homogenate was added (triplicates per sample). The reaction was started by addition of 20 μl oxaloacetate (500 μM final). The 412 nm absorbance was measured in a SPECTRAmax Plus 384 plate reader (Molecular Devices, UK) for 10 min in cycles of 7 s at RT. The concentration of TNB^{2-} was calculated with ε_{412} = 13,600 $M^{-1}cm^{-1}$.

Differential thiol labelling for LC-MS

Mitochondrial membranes and mitochondria

Mitochondrial membranes or isolated mitochondria were pre-incubated (as specified), pelleted (17,000 × *g*, 3 min, 4°C) and resuspended in KPi buffer (50 mM KPi, pH 7.8 at 30°C) (if not otherwise specified), containing IAM (light or heavy, 20 mM), MMTS (0.5 mM), d_5-NEM (10 mM) or TPP-IAM (20 mM). Samples were labelled for 5 min on ice (if not otherwise indicated) and pelleted (17,000 × *g*, 3 min, 4°C). Samples were washed with KPi buffer and centrifuged as before.

- IAM, NEM or TPP-IAM labelled samples were denatured in lysis buffer (50 mM NaPi pH 7.8, 2% SDS) containing 2–10 mM TCEP and 10 or 20 mM IAM (different isotope version than in the first step), NEM or d_{15}-TPP-IAM and labelled for 15–30 min at 37°C.
- MMTS labelled samples were denatured in lysis buffer containing 50 mM light IAM and labelled for 15 min at 37°C. Then samples were passed twice through Micro Bio-Spin 6 columns (Bio-Rad, UK), that were pre-equilibrated with lysis buffer in order to remove excess IAM. Next, 10 mM TCEP was added and samples were incubated for 5 min at 37°C, before 20 mM heavy IAM was added and samples were alkylated for 15 min at 37°C.

Finally, SDS sample buffer (Laemmli) was added and proteins were separated by SDS-PAGE followed by in-gel trypsin cleavage. Alternatively, proteins were precipitated with 30 vol. of ice-cold ethanol before in-solution trypsin proteolysis.

Sequential activity assay and labelling

BHMMs or RHM were deactivated, shaking at 37°C for 20 or 30 min, respectively. Active samples were prepared by reactivating deactive samples in the presence of NADH (1 mM, BHMMs) or glutamate and malate (5 mM) and 0.5 mM ADP (RHM) for 5 min on ice in KPi buffer (50 mM KPi, pH 7.8 at 30°C; if not specified otherwise). Mitochondria were additionally permeabilized with alamethicin (30 μg/μl) and $MgCl_2$ (2.5 mM) (Gostimskaya et al., 2003). RHM or BHMMs were pelleted (17,000 × *g*, 3 min, 4°C) and resuspended

in ice-cold KPi buffer containing IAM (20 mM) or MMTS (0.5 mM) and samples were labelled for 5 min on ice. Samples were pelleted (17,000 × *g*, 3 min, 4 °C), washed once with KPi buffer (containing 1 mM GSH if indicated) and then resuspended in KPi buffer and NADH/dQ oxidoreductase activity was measured (at 10 μg protein/well) as detailed above. Following the activity assay, samples (of three replicates) were retrieved from the 96-well plate, combined and split into two for each condition. Then, samples were subjected to the second labelling step (as specified in the differential labelling section) and further processed for analysis by UTP or MRM.

Labelling of Cys39 during respiration

BHMMs/MHMMs (35/28.5 μg) were incubated in 450 μl of turnover buffer (KPi buffer (50 mM KPi, pH 7.8 at 30°C) containing 1.5 μM Cyt *c* and 15 μg/ml alamethicin (both final for 500 μl volume)), containing no substrate, 10 mM NADH or 10 mM succinate and if indicated inhibitors (rotenone (2 μM), piericidin A (2 μM) or antimycin A (5 μM)). Deactive samples were deactivated for 20 min at 37°C in 50 μl KPi, prior to resuspending in turnover buffer. Samples were incubated at 37°C in open Eppendorf tubes and shaken vigorously. After 1 min of incubation 50 μl of 200 mM IAM (20 mM final) were added and samples were labelled for 5 min during respiration. Samples were rapidly transferred onto ice, pelleted (17,000 × *g*, 3 min, 4°C) and washed with 1 ml KPi buffer. Proteins were denatured and labelled with heavy IAM (as described above: differential thiol labelling) and analyzed by UTP or MRM.

Method development and validation. In order to quantify the Cys39 exposure on complex I in BHMMs, exposed Cys39 residues on intact complex I are initially labelled with (light) IAM. Then complex I is denatured and reduced to expose all remaining Cys39 for labelling with heavy ($^{13}C_2$, 2-d_2) IAM. Following in-gel or in-solution trypsin cleavage, light and heavy isotope labelled Cys39-containing ND3 peptides are quantified by LC-MS (Figure 3A). Both the light and heavy IAM labelled Cys39-containing ND3 tryptic peptides (TSPYEC(carbamidomethyl)GFDPMGSAR) were detected when this analysis was applied to BHMMs (Figure S2A). The peak volumes of the monoisotopic peak from light and heavy labelled peptides were compared from extracted ion chromatograms (XIC), enabling us to quantify the percentage exposure of Cys39 in complex I in BHMMs (Figure S2B). Quantifying light and heavy labelled peptides using the MaxQuant software (v1.6.10.43 and v1.6.17.1), gave comparable results (data not shown). Additional technical information as well as calculated monoisotopic peptide masses are specified in the STAR Methods section below.

To develop a high-throughput assay without the necessity for scanning the whole proteome, we set up a targeted multiple reaction monitoring (MRM) method on a Waters TQ-S triple quadrupole mass spectrometer for the detection of the H/L-labelled ND3 tryptic peptide. For this we focused on the tryptic Cys39-containing ND3 peptide from rat and mouse (peptide sequence is identical for both species: ANPYECGFDPTSSAR), allowing for the analysis of samples from mouse heart mitochondrial membranes, isolated mitochondria and *in vivo*/tissue experiments in mice. For the method development, we used synthetic light and heavy IAM labelled ND3 peptides, as well as a heavy internal standard (IS) peptide containing d_4-alanines (Figures S2C–S2E). This approach showed that the MS response of the heavy or light labelled peptide relative to the IS was identical, as expected. Thus, this approach would in principle enable us to construct standard curves and thereby provide absolute quantification of the H and L peptides (Figure S2E). However, for our purposes this was not necessary as MS response was identical for H and L peptides when measured by MRM and therefore enabled us to calculate the proportions of each peptide and thus quantify the level of occupancy of Cys39 (Figure S2E). Therefore, we have developed a viable MRM method to quantify the exposure of Cys39 in complex I from mouse and rat samples.

From previous reports, we had expected to find negligible Cys39 labelling in the catalytic A-state of complex I. Therefore, we carried out numerous controls to eliminate possible artifactual explanations for our findings that Cys39 was largely exposed by complex I in the catalytic A-state. We tested if prolonged reactivation with NADH would increase complex I activity compared to deactive samples, which was not the case (Figure S3A). Next, we explored the possibility that a fraction of complex I was enclosed by a membrane and therefore NADH was unable to reactivate that portion of complex I. This fraction would appear as a fraction with exposed Cys39 in our labelling approach. However, the NADH/dQ oxidoreductase activity was unchanged by addition of alamethicin (Figure S3B), which would allow NADH access into closed membrane compartments. Furthermore, we assessed if the deactivation or activation procedures would impact on complex I integrity that would result in broken or disassembled complex I which might distort measurement of the apparent Cys39 exposure by appearing as exposed in the active preparation. Analyzing complex I by BN-PAGE did not show any evidence for complex I disruption (Figure S3C). We also explored the hypothesis of unassembled or disassembled ND3 subunits, which might have occurred during membrane preparation. To do this we employed a labelling strategy involving two-dimensional electrophoresis to select only intact complex I for differential labelling and MS analysis. Following the initial labelling with IAM, complex I was solubilized with DDM (*n*-Dodecyl β-D-maltoside) or digitonin, resolved by BN-PAGE and intact complex I as either monomer or supercomplexes was subjected to second labelling. DDM treated catalytically active samples gave qualitatively similar results to previous data (Figure S3D). The same was found for digitonin treated samples with no observable difference between complex I in its monomeric or supercomplex forms observable (Figure S3D). This clearly indicated that Cys39 is exposed to a large extent in catalytically active and fully assembled complex I. Next, we applied the combined assessment of complex I activity (NADH:dQ oxidoreductase activity) and Cys39 exposure to test if differential thiol labelling with the more rapid and reversible MMTS, followed by reductive replacement with iodoacetamide and LC-MS analysis, would provide the same results. We found that Cys39 exposure again was high in catalytically active samples (Figure S3E). Further, we showed that the combined analysis provided similar results, independent of the buffer system used for the experiment (Figure S3F). Finally, we tested the completeness of labelling of exposed Cys39 with IAM for 5 min on ice and found that extending the labelling to 1 hr only marginally increased the proportion of Cys39 detected as exposed (Figure S3G).

RHM (50 μg) were incubated in 100 μl KCl buffer (120 mM KCl, 10 mM HEPES, 1 mM EGTA, pH 7.2 at 37°C), containing no substrate, 10 mM glutamate/malate (each) or 10 mM succinate and if indicated inhibitors (rotenone (2 μM), piericidin A (2 μM) or antimycin A (5 μM)). Samples were incubated at 37°C in open Eppendorf tubes and shaken vigorously. After 1.5 min of incubation 11.1 μl of

200 mM IAM (20 mM final) were added and samples were labelled for 10 min during respiration. Deactive samples were incubated for 30 min at 37°C prior to addition of iodoacetamide. Samples were transferred onto ice, pelleted (17,000 × *g*, 3 min, 4°C) and washed with 1 ml KPi buffer. Proteins were denatured and labelled with heavy IAM (as described above: differential thiol labelling) and analyzed by UTP.

Tissue homogenate

For labelling of exposed cysteine residues, frozen heart tissue (~5 mg) was weighed into lysis tubes (Precellys, CK14, Bertin Instruments, France or 1.4 mm ceramic bead filled tubes, Fisher Scientific, USA), pre-cooled on dry ice. The tissue was homogenized in 400 µl of ice-cold KPi buffer (50 mM KPi, pH 7.8 at 30°C) containing 20 mM (or 50 mM) light IAM and 10 mM of TCEP, using a tissue homogenizer (Precellys 24, Bertin Instruments, France) for 15 s at 6500 rpm. The homogenate was transferred into pre-cooled Eppendorf tubes and thiols were labelled for 5 min on ice (if not otherwise specified). The reaction was quenched by adding 1 ml of KPi buffer and pelleting the membranous fraction (17,000 × *g*, 5 min, 4°C). Pellets were washed with 1 ml of KPi buffer and centrifuged as before. Residual thiols were labelled by resuspending the pellets in 45 µl of lysis buffer (2 or 4% SDS, 50 mM NaPi, pH 7.8) containing 20 mM of heavy ($^{13}C_2$, 2-d_2) IAM and 10 mM TCEP and incubation at 37°C for 30–60 min. After addition of SDS sample buffer (4× Laemmli), proteins were separated by SDS-PAGE or frozen at −20°C until further processing. Proteins were cleaved in-gel with trypsin and peptides were analyzed by MRM or UTP.

Method development. In order to measure NADH/dQ oxidoreductase activity in tissues, first tissue homogenate was prepared from clamp-frozen tissue and snap-frozen (Figure S5A). Tissue homogenate was thawed on ice and NADH/dQ oxidoreductase activity was measured. This approach allowed for the comparison of multiple samples in one assay without delays due to sample processing which might impact experimental results. First, we explored complex I integrity in tissue homogenate by assessing levels of complex I in its monomeric and supercomplex forms, which were found to be unchanged in normoxic and ischemic mouse heart (Figures S5B and S5C). Therefore, we reasoned that all complex I activity changes can be attributed to the A/D transition. To ensure that the tissue homogenization was sufficient to disrupt membranes and make all complex I accessible to NADH, DDM was titrated into the activity assay. Low DDM concentrations (0.025%) shifted the rate of highest NADH oxidation to the beginning of the assay, allowing for easier quantification, while higher concentrations impaired complex I activity (Figure S5D). In contrast, citrate synthase activity was not altered by addition of DDM, indicating successful disruption of membranous compartments by homogenization (Figure S5E). To explore the effect of DDM on the catalytic A/D transition, NADH/dQ oxidoreductase activity in preparations of catalytically active and deactive BHMMs was assessed. This demonstrated that DDM selectively inhibits reactivation of catalytically deactive complex I, more effectively than thiol-reactive agents (Figure S5F). The result is intriguing as DDM was found to bind into the CoQ channel and proposed to promote deactivation (Grba and Hirst, 2020), similar to Triton X-100, which in contrast seems to inhibit complex I irrespective of its conformational state (Ushakova et al., 1999). Consequently, DDM seemed to have the necessary properties to stabilize the catalytic complex I state in tissue homogenates. Next, NADH/dQ oxidoreductase activity was measured with and without addition of DDM, to stabilize catalytically deactive complex I, in normoxic and ischemic mouse heart homogenates. These hearts were either clamp-frozen rapidly following excision of the heart or exposed to ischemia by incubation at 37°C. In the absence of DDM a lag in NADH oxidation was observed in ischemic samples, which can be attributed to slow complex I reactivation, similar as the lag observed with BHMMs (Figure S5G). In addition, there was a small reduction in complex I activity upon ischemia. In contrast, the addition of DDM resulted in a strong reduction of complex I activity in ischemic samples, compared to the normoxic control, consistent with stabilization of catalytically deactive complex I (Figure S5H). Consequently, DDM was added routinely to tissue homogenate for complex I activity measurements. We also found a gradual deactivation of complex I with increasing length of ischemia, consistent with previous work (Figure S5I) (Gorenkova et al., 2013).

We also aimed at exploring and quantifying Cys39 exposure in tissue samples and correlating it with complex I activity. To do this, the MRM mass spectrometry method was used as described in Figure S2 and in the STAR Methods section . Clamp frozen tissue samples were lysed in IAM containing buffer and labelled on ice, prior to denaturation of all proteins and labelling of residual thiols (Figure S6A). Proteins were cleaved with trypsin and analyzed by LC-MS using MRM or UTP. First, we tested if 20 mM IAM, as used in previous experiments, would be sufficient to completely label all thiols of ~5 mg tissue within 5 min on ice. We assumed that 1 mg of heart tissue protein, corresponding to ~5 mg tissue wet weight, would contain 10–20 µg of complex I (~10% of mitochondrial protein mass) (Benson, 1955; Pfanner et al., 2019; Pryde and Hirst, 2011). Furthermore, we calculated that 100 nmol/mg protein of exposed thiols, would result in a free thiol concentration of 250 µM if ~5 mg tissue was homogenized in a volume of 400 µl (Requejo et al., 2010). Consequently, we incubated 250 µM reduced glutathione (GSH) with 20 mM IAM for different periods on ice and measured the proportions of labelled (GS-CAM) and unlabeled (GSH) glutathione by direct infusion into a mass spectrometer. We found a rapid time dependent increase in labelling over the first 5 minutes which started to plateau at around 7.5 min, indicating that 5 min of labelling were sufficient (Figure S6B). In an initial test, we labelled exposed cysteines in normoxic and ischemic mouse heart with either 20 or 50 mM IAM. Although, significantly lower Cys39 exposure was detected in tissue homogenates than in BHMMs (Figure S6C), Cys39 exposure in ischemic tissues was markedly increased compared to normoxic controls, as was expected by a shift of complex I towards the catalytic D-state. The amount of exposed Cys39 in samples labelled with 20 mM IAM was much lower than in samples labelled with 50 mM, indicating that the low Cys39 exposure observed with 20 mM is due to incomplete labelling. This was confirmed by extending the labelling period with 20 mM IAM up to 1 hr on ice, which resulted in increased labelling (Figure S6D). Nevertheless, as sufficient labelling was achieved with 20 mM IAM during 5 min, we decided to routinely used this setting as the ratio of Cys39 exposure may be distorted by prolonged labelling. Therefore, we have set up a differential labelling strategy for tissues which allows to establish Cys39 exposure by complex I in a semi-quantitative way.

Combined with 2D electrophoresis

To ensure analysis of intact complex I as well as to differentiate between its monomeric and supercomplex populations, two-dimensional electrophoresis was combined with differential labelling. Hereby, exposed thiols of native proteins in BHMM (see mitochondrial membrane incubations) were labelled in an initial step with 20 mM light IAM for 5 min on ice. Tissue samples were homogenized and labelled with 20 mM light IAM and 10 mM TCEP for 5 min on ice (see subsection tissue homogenate, above). The labelling was quenched by centrifugation (17,000 × *g*, 5 min, 4°C) and washing the membranous fraction with KPi buffer (50 mM KPi, pH 7.8 at 30°C). Then, proteins were extracted as outlined below (see BN-PAGE section) and resolved by BN-PAGE, followed by in-gel complex I flavin staining. Gel sections containing complex I (in monomeric or supercomplex forms as specified) were then excised with a scalpel and sliced into very small pieces and transferred into Eppendorf tubes. Next, ~40 μl of lysis buffer (4% SDS, 50 mM NaPi, pH 7.8) containing 20–50 mM of heavy IAM and 10 mM TCEP was added, the solution was thoroughly squashed with a cell lysis pestle and thiols were labelled for 30–45 min 37°C. Finally, SDS sample buffer (4× Laemmli) was added to the samples and all liquid and gel-remnants were loaded into the wells of a SDS-PAGE gel and proteins were resolved. Sample were cleaved in-gel with trypsin and peptides were analyzed by UTP or MRM.

Fluorescent labelling of Cys39 exposure

Standard protocol (this study)

BHMMs were resuspended in KPi buffer (50 mM KPi, pH 7.8 at 30°C) and deactivated (20 min at 37°C) or activated (kept on ice for 15 min followed by incubation with 1 mM NADH on ice. Membranes were pelleted (17,000 × *g*, 3 min, 4°C) and exposed thiols were blocked with 10 mM NEM (in KPi buffer) for 15 min on ice. Membranes were washed with 35 mM cysteine and subsequently with plain KPi buffer. Then, membranes were deactivated (20 min at 37°C) and labelled with 0.5 mM N-fluorescein maleimide for 10 min at RT in the dark. Membranes were washed with 35 mM cysteine and subsequently twice with plain KPi buffer. Next, proteins were extracted with 1% DDM and separated by BN-PAGE. The band corresponding to complex I was excised, cut into very small pieces, sample buffer (4× Laemmli) was added and all liquid and gel-remnants were loaded into the wells of a SDS-PAGE gel and proteins were resolved. The fluorescent labelling in the gel was scanned using an Amersham Typhoon RGB scanner. Then, the gel was fixed (50% methanol and 10% acetic acid) and stained with QC colloidal brilliant blue staining solution (Bio-Rad, UK).

Protocol based on Galkin et al. (2008)

BHMMs were activated in KPi buffer (50 mM KPi, pH 7.8 at 30°C; incubated in presence of 0.4 mM NADPH, shaking at RT for 30 min). Membranes were washed and exposed thiols were blocked with 30 mM NEM (incubating for 30 min at 15°C) in KPi buffer (pH 9). Membranes were washed with 35 mM cysteine and subsequently with plain KPi buffer (pH 7.8). The samples were split into two. One fraction (active) was incubated on ice for 90 min while the other fraction (deactive) was incubated for 90 min at 37°C. Then, membranes were labelled with 0.5 mM N-fluorescein maleimide for 20 min at 15°C in the dark. Membranes were washed with 35 mM cysteine and subsequently twice with plain KPi buffer. Next, proteins were extracted with 1% DDM and separated by BN-PAGE. The band corresponding to complex I was excised, cut into very small pieces, sample buffer (4× Laemmli) was added and all liquid and gel-remnants were loaded into the wells of a SDS-PAGE gel and proteins were resolved. The fluorescent labelling in the gel was scanned using an Amersham Typhoon RGB scanner. Then, the gel was fixed (50% methanol and 10% acetic acid) and stained with QC colloidal brilliant blue staining solution (Bio-Rad, UK) (Galkin et al., 2008).

Combined protocol (three conditions)

BHMMs were resuspended in KPi buffer (50 mM KPi, pH 7.8 at 30°C) and deactivated (30 min at 37°C) or activated (kept on ice for 25 min followed by incubation with 1 mM NADH on ice. Membranes were pelleted (17,000 × *g*, 3 min, 4°C) and exposed thiols were blocked with 0.5 mM NEM (in KPi buffer) for 30 min on ice. Membranes were washed twice with 35 mM cysteine and subsequently once with plain KPi buffer. Then samples were kept either on ice or deactivated at 37°C for 30 min, pelleted (17,000 × *g*, 3 min, 4°C) and resuspended in 50 μl KPi containing 0.5 mM N-fluorescein maleimide or Cy5-NEM dye (sufficient dye to label 50 μg of unlabeled protein, according to manufacturer) and labelled for 30 min on ice in the dark. Membranes were washed twice with 35 mM cysteine and subsequently once with plain KPi buffer. Next, proteins were extracted with 1% DDM and separated by BN-PAGE. The band corresponding to complex I was excised, cut into very small pieces, sample buffer (4× Laemmli) was added and all liquid and gel-remnants were loaded into the wells of a SDS-PAGE gel and proteins were resolved. The fluorescent labelling in the gel was scanned using an Amersham Typhoon RGB scanner. Then, the gel was fixed (50% methanol and 10% acetic acid) and stained with QC colloidal brilliant blue staining solution (Bio-Rad, UK).

Labelling for comparative LC-MS analysis

Samples were subjected to two different labelling regimens using the following labelling agents:

1. Light-IAM → Heavy-IAM → NEM
2. NEM → Light-IAM → Heavy-IAM

BHMMs (100 μg protein/sample) were resuspended in KPi buffer (50 mM KPi, pH 7.8 at 30°C) and deactivated (20 min at 37°C) or activated (kept on ice for 15 min followed by incubation with 1 mM NADH on ice. Membranes were pelleted (17,000 × *g*, 3 min, 4°C) and exposed thiols were blocked with 20 mM L-IAM(1) or 10 mM NEM(2) for 30 min on ice (in 100 μl KPi buffer). Membranes were washed twice with 35 mM cysteine and subsequently with plain KPi buffer. Then, membranes were resuspended in 50 μl KPi buffer and deactivated (30 min at 37°C), pelleted and labelled with 20 mM H-IAM(1) or 20 mM L-IAM(2) for 30 min at RT (in 50 μl KPi buffer).

Membranes were washed with 35 mM cysteine and subsequently with plain KPi buffer. Proteins were extracted with 1% DDM and separated by BN-PAGE. The complex I gel band was excised and sliced into very small pieces and proteins were denatured by addition of 15 μl 4× Laemmli loading dye containing 1 mM TCEP and 20 mM NEM(1) or 20 mM H-IAM(2). Residual thiols were labelled for 30 min at RT and proteins were separated by SDS-PAGE. In-gel trypsin cleavage was performed as detailed below and samples were analyzed by UTP.

Mitochondrial ROS measurements

ROS production by isolated RHM was measured via AmplexRed, which is converted into the fluorescent molecule resorufin by horseradish peroxidase (HRP) in presence of H_2O_2. Superoxide dismutase (SOD) is added to ensure that superoxide, produced by mitochondria, is dismutated into H_2O_2 and therefore is detected in the assay. First, active and deactive samples were prepared by incubating RHM in KCl buffer (120 mM KCl, 10 mM HEPES, 1 mM EGTA, pH 7.2 at 37°C) for 30 min at 37°C to deactivate complex I, or for 30 min on ice with 10 min of incubation in the presence of 1 mM glutamate/malate (each) with a short incubation (3 min) at RT to initiate respiration and fully activate complex I. Then IAM (20 mM final) was added to label exposed cysteine residues for 10 min on ice. Mitochondria were pelleted (17,000 × *g*, 3 min, 4°C) and washed once with 1 ml KCl buffer. A 96-well plate was prepared by adding 80 μl of KCl buffer containing HRP (20 μg/ml), SOD (40 μg/ml) and BSA (0.2 mg/ml) (all final concentrations) into the wells on ice. Then, mitochondria (30 μg protein in 20 μl in KCl buffer) were plated and the plate was transferred onto a heat block at 37°C to warm the plate quickly and respiration was immediately initiated by addition of KCl buffer containing 10 mM succinate, AmplexRed (12.5 μM) and ±5 μM FCCP (or ethanol). Formation of the fluorescent resorufin product was monitored with a ClarioSTAR Plus (BMG Labtech, Germany) fluorescent plate reader at λ_{Ex} = 560-8 nm and λ_{Em} = 590-8 nm during 150 cycles of 12 s at RT and with 3 s of orbital shaking before each cycle. Fluorescence was detected in the top reading mode using a focal height of 7.5 mm and a gain of 2000. For the calculation of absolute H_2O_2 concentrations a standard curve was established using known concentrations of H_2O_2 ranging from 0 to 2.5 μM, while omitting SOD and mitochondria from the reaction solution. The concentration of H_2O_2 was determined after dilution in H_2O by measuring the absorbance at 240 nm with a UV-2600 UV-Vis spectrophotometer (Shimadzu, Japan) and calculating the final concentration with ε_{240} = 43.5 $M^{-1}cm^{-1}$.

Blue native (BN)-PAGE

Protein extraction

Proteins for BN-PAGE analysis were extracted from the membranes in samples (BHMMs or membranous fraction of tissue homogenate), by resuspending the membrane pellet in BN extraction buffer (0.75 M aminocaproic acid, 50 mM BisTris-HCl, pH 7.0 at 4°C)) containing either 1% (w/v) DDM or 8 g/g protein digitonin, while avoiding bubble formation. Samples were incubated for 10 min on ice and the insoluble fraction was subsequently pelleted by centrifugation (17,000 × *g*, 15 min, 4°C). The supernatant was combined with BN loading dye (5% (w/v) Serva Blue G in 0.5 M aminocaproic acid), carefully mixed and proteins were resolved by BN-PAGE.

Separation of native proteins by BN-PAGE

Protein extracts were resolved by precast NativePAGE 3–12% Bis-Tris gels (10-well; Thermo Fisher Scientific, UK). Proteins were separated using BN cathode buffer and BN anode buffer (100 V, ~4–6 hrs, 4°C) in a XCell SureLock Mini-Cell Vertical Electrophoresis cell (Thermo Fisher Scientific, UK) and electrophoresed until the dye front reached the bottom of the gel.

In-gel complex I flavin staining with NTB

For complex I in-gel staining, the BN-PAGE gel was rinsed initially with Milli-Q water and then incubated in assay buffer (2.5 mg/ml nitrotetrazolium blue (NTB) and 0.1 mg/ml NADH in 5 mM Tris-HCl, pH 7.4 at RT) for 10 min (Wittig et al., 2007). Then, the gel was rinsed three times for 5 min with Milli-Q water.

SDS-PAGE

Proteins were solubilized in Laemmli sample buffer. For second dimension protein separation, the complex I specific band was precisely excised from the BN gel and cut into very fine slices. Laemmli sample buffer (and if indicated reduction and alkylating agents) were added and proteins were denature/labelled at 37°C. The total mixture of gel pieces and sample buffer was transferred into the loading wells of an SDS-gel.

Proteins samples for analysis by mass spectrometry were loaded into the loading wells and separated on 12% Mini-PROTEAN TGX Gels (Bio-Rad, UK) in SDS-separation buffer (25 mM Tris, 192 mM glycine, 3.5 mM SDS) at 120 V for ~1 hr. The gel was fixed in 50% methanol, 10% acetic acid and proteins were stained with QC colloidal brilliant blue staining solution (Bio-Rad, UK), followed by washing the gel with Milli-Q water.

In-gel protein cleavage and desalting

The in-gel protein cleavage protocol is based on the method described by Shevchenko et al. (1996, 2006). Throughout the entire protocol autoclaved Milli-Q water and Eppendorf Protein LoBind tubes (Eppendorf, Germany) were used. Excised gel pieces, containing the proteins of interest (region between 10 and 20 kDa for ND3), were rinsed in water and cut into small 1 mm cubes using a scalpel. The cubes were incubated for 30 min in water followed by two incubations in 20 mM Tris-HCl (pH 8) for 45 min. Then, gel cubes were partially dehydrated by incubation in 50% acetonitrile (ACN), 20 mM Tris-HCl (pH 8), followed by complete dehydration with pure ACN and evaporation of all residual liquid during 30–60 min in a SpeedVac at 40°C. Gel pieces were rehydrated with trypsin solution (12.5 ng/μl trypsin, 5 mM $CaCl_2$, 20 mM Tris-HCl, pH 8). Proteins were cleaved overnight at 37°C and peptides were extracted twice in

60% ACN, 4% formic acid (FA) and then the gel cubes were completely dehydrated with pure ACN. All extraction fractions were combined and dried in a SpeedVac at 40°C and subsequently resuspended in 0.1% trifluoroacetic acid (TFA). Samples were desalted with C18 Bond Elut OMIX tips (Agilent, UK) (this step was omitted in the complex I cysteine survey to reduce peptide loss). The tips were washed initially with 50% ACN and then equilibrated with 0.1% TFA. Samples were aspirated and passed through the C18 matrix at least ten times. Peptides were washed with 5% ACN, 0.1% TFA and eluted in 60% ACN, 0.1% FA. Peptides were dried again and redissolved in the respective buffers for analysis by UTP or MRM.

In-solution protein cleavage

For in-solution trypsin cleavage, labelled proteins were precipitated with 30 vol. of ice-cold ethanol for 30 min on dry ice. Proteins were pelleted for 15–30 min at 17,000 × *g* at 4°C. The protein pellet was washed with 0.5 ml of ice-cold ethanol, proteins were incubated for 15 min on dry ice and pelleted by centrifugation as before. The supernatant was discarded and excess ethanol was evaporated at 37°C on a heat block. Proteins resuspended in 12.5 ng/μl trypsin in 50 mM ammonium bicarbonate buffer (pH 7.8) and cleaved over-night. Peptides were subsequently dried and redissolved in the respective buffers for analysis by UTP or MRM.

LC-MS analysis of labelled ND3 peptides

Cys39 exposure was determined by differential labelling followed by LC-MS analysis using UTP or MRM. The ND3 tryptic peptide containing Cys39 differs amongst species. The sequence is conserved for rats and mice, while the bovine sequence differs. The predicted peptide masses of light and heavy ($^{13}C_2$, 2-d_2) iodoacetamide (IAM), light and heavy (d_5-)*N*-ethylmaleimide (NEM) as well as light and heavy (d_{15}-)TPP-IAM labelled peptides are listed below.

Untargeted proteomics (UTP)

Peptides were resuspended in 3% ACN, 0.1% TFA buffer and were fractionated by liquid chromatography on an Acclaim PepMap C18 reversed-phase column (Thermo Scientific, UK), 50 μM internal diameter, 150 mm length in a Proxeon EASY-nLC 1000 system using a gradient of 5–40% ACN in 0.1% (v/v) formic acid, over 84 min at a flow rate of 300 nl/min, followed by an increase in acetonitrile concentration to 90% and re-equilibration with 5% ACN within a 105-minute period. The eluate was transferred in-line to a Q-Exactive Plus Orbitrap mass spectrometer (Thermo Scientific, UK). Peptides were analyzed by positive ion electrospray mass spectrometry using a method programmed to fragment the top 10 most abundant multiply charged peptide ions each second. Full scan MS data (400–1600 m/z) were recorded at a resolution of 70,000 with an automatic gain control (AGC) target of 1×10^6 ions and a maximum ion transfer of 20 ms. Ions selected for MS/MS were analyzed using the following parameters: resolution 17,500; AGC target of 5×10^4; maximum ion transfer of 100 ms; 2 m/z isolation window; for HCD a normalized collision energy 28% was used; and dynamic exclusion of 30 s. A lock mass ion (polysiloxane, m/z = 445.1200) was used for internal MS calibration. For protein identification the fragment patterns were compared to the UniProt database using the Mascot search engine with the Thermo Proteome Discoverer (v1.4) software. Relative quantification was performed by comparing the peak area of XICs (extracted ion chromatograms) for the monoisotopic peak using the Thermo Xcalibur software or alternatively by using the MaxQuant (v1.6.10.43 or v.1.6.17.1) software.

Species	Sequence	Label	Isotope	Label	M^{2+} (m/z)	M^{3+} (m/z)
Bovine	TSPYECxGFDPMGSAR	IAM	–	C2 H3 N O	837.8480	
			$^{13}C_2$, 2-d_2	13C2 2H2 H N O	839.8576	
		NEM	–	C6 H7 N O2	871.8611	
			d_5	C6 2H5 H2 N O2	874.3768	
		TPP-IAM	–	C25 H27 N O P	–	669.2883
			d_{15}	C25 H12 2H15 N O P	–	674.3196
Rat/Mouse	ANPYECxGFDPTSSAR	IAM	–	C2 H3 N O	836.3570	
			$^{13}C_2$, 2-d_2	13C2 2H2 H N O	838.3667	

Targeted MRM analysis of ND3 peptides

LC-MS/MS analyses of iodoacetamide labelled ND3 (tryptic) peptides was performed using a Xevo TQ-S triple quadrupole mass spectrometer (Waters, UK). Samples (in 20% ACN, 0.1% FA) were kept at 8°C prior to sampling of 5–10 μl of the peptide extracts by the autosampler into a 15 μl flow-through needle. Separations were performed on a I-Class ACQUITY UPLC BEH C18 column (1 × 50 mm, 130 A, 1.7 μm; Waters, UK) with a UPLC filter (0.2 μm; Waters, UK) at 30°C using a ACQUITY UPLC I-Class system (Waters, UK). The mobile phases were MS solvent A (5% ACN, 0.1% FA) and B (90% ACN, 0.1% FA) at a flow rate of 0.2 ml/min with the following gradient (the proportion of MS solvent B is given in %): 0–0.3 min: 5%, 0.3–3 min: 5–100%, 3–4 min: 100%, 4–4.1 min: 100-5%, 4.1–5 min: 5%. The eluate was analyzed by MS for the complete 5 min UPLC gradient. Peptides were detected by multiple reaction monitoring (MRM) with electrospray ionization in positive ion mode using the following MS method settings: source spray voltage – 3.0 kV; cone voltage – 2 V; ion source temperature – 150°C; collision energy – 25 V. Nitrogen and argon were used as the curtain and the collision gases, respectively. MS/MS transitions used for quantification are shown below.

ND3 peptide	IAM	Transition (m/z)
light	light	836.7 > 744.0
	$^{13}C_2$, 2-d_2	838.7 > 746.0
d_8 (2x d_4 alanine)	$^{13}C_2$, 2-d_2	842.8 > 748.2

The product mass corresponds to the individual y_{13}^{2+} fragment ion.

Standard curves were prepared with known amounts of light and heavy iodoacetamide labelled ND3 peptide, spiked with internal standard (IS) (heavy labelled d_8-ND3 peptide), were prepared. The peak area of the ND3 peptides and IS of samples were quantified using the MassLynx 4.1 software. The standard curves confirmed, that light and heavy labelled ND3 tryptic peptides were detected with equal sensitivity, allowing for relative quantification of these peptides.

Iodoacetamide labelling kinetics of GSH

In order to determine the labelling kinetics of GSH with IAM by MS, GSH (250 μM) was incubated in 50 mM $(NH_4)HCO_3$ (ammonium bicarbonate) buffer (pH 7.8) on ice and the reaction was started by addition of 20 mM IAM. The reaction mix was incubated on ice and samples of 20 μl were taken at indicated time points and mixed 1:1 with dithiothreitol (DTT) at a final concentration of 100 mM to quench the reaction, vortexed and instantaneously snap-frozen in liquid nitrogen. For the zero minute time point, 20 μl of 200 mM DTT was incubated with 10 μl of 100 mM $(NH_4)HCO_3$ buffer (pH 7.8) and 5 μl of 1 mM GSH. As soon as 5 μl of 80 mM IAM were added, the sample was vortexed and snap-frozen. Samples were thawed on ice upon addition of 960 μl of MS sample solvent (20% ACN, 0.1% FA). GSH and GS-CAM (GS-carbamidomethyl reaction product) levels were measured by direct infusion into a Xevo TQ-S mass spectrometer (Waters, UK) operating in negative ion electrospray mode. Spectra were recorded in a range of 50–1200 m/z every 0.5 s for 1 min, while samples were introduced with a flow rate of 50 μl/min.

MS Parameters for the direct infusion of GSH and GS-CAM

Parameter	Setting
Desolvation temperature	150°C
Capillary voltage	3 kV
Cone voltage	set to 0 V (was actually 25 V)
Source offset	set to 0 V

The intensities for GSH (308 m/z) and GS-CAM (363 m/z) peaks were measured and the proportion was calculated.

Comparative analysis of all cysteine residues

FASTA files of the protein sequences for the following species (for which complex I was shown to have the ability to undergo the A/D transition) were obtained from the Uniprot database (if available) and aligned using Clustal Omega (Sievers et al., 2011): *Homo sapiens*, *Bos taurus*, *Sus scrofa*, *Ovis aries*, *Rattus norvegicus*, *Mus musculus*, *Gallus gallus*, *Lithobates catesbeiana*, *Cyprinus carpio*. Cysteine residues in the mouse sequences were used as reference and were numbered according to their position in the complete mitochondrial precursor sequence. Conserved cysteine residues were identified in the sequence alignments and marked. The solvent accessible surface exposure of the γ-Sulfur atom was calculated for all modelled cysteine residues in the cryoEM structures of active (PDB:6G2J) and deactive (PDB:6G72) mouse complex I (Agip et al., 2018) using MacPymol (1.8.4.0). A 5 $Å^2$ cut-off was applied to classify cysteine residues and estimate their chance of being modified and detected as exposed (James et al., 2018). Cysteines found within the cleavable mitochondrial targeting sequence and cysteines contributing to FeS clusters were identified (cryoEM structures of active (PDB:6G2J) and deactive (PDB:6G72) mouse complex I (Agip et al., 2018)) and indicated. In addition, cysteine residues modeled as intramolecular disulfides in these structures and/or predicted to form intramolecular disulfides (Uniprot PROSITE-ProRule annotation) were indicated. Cysteine exposure was determined by differential labelling of active and deactive complex I in BHMMs with IAM (as detailed above). The proportions of exposed cysteine residues (light IAM labelled) were determined using MaxQuant (v.1.6.17.1). Cysteine residues that can only be found in tryptic peptides that contain multiple cysteine residues were indicated. Cysteines in peptides with <7 amino acids and/or <400 m/z (MH^{2+}) or >6000 Da monoisotopic mass, or more than 3 cysteines were classified as undetectable.

QUANTIFICATION AND STATISTICAL ANALYSIS

All data are represented as mean ± S.E.M. or mean ± range (n = 2), as indicated in the corresponding figure legend. The given 'n' values represent either the number of biological replicates or the number of independent experiments (or as specified otherwise) and are indicated in the figure legends. Within activity assays, usually technical replicates (duplicates or triplicates) of the same

CellPress
OPEN ACCESS

sample were analyzed. Data was visualized and statistical analysis was performed using the Prism 9.0 software (Graphpad, USA). For the comparison of two independent datasets two-tailed unpaired Student's t tests were applied, assuming equal variance. Multiple datasets were statistically compared via one- or two-way analysis of variance (ANOVA) and the appropriate correction for multiple comparisons (indicated in the figure legend) was applied. The p (associated probability) value was considered significant if < 0.05 and significance was indicated as follows: *$p < 0.05$; **$p < 0.01$; ***$p < 0.001$, ****$p < 0.0001$.

Cell Chemical Biology, Volume *29*

Supplemental information

ND3 Cys39 in complex I is exposed during mitochondrial respiration

Nils Burger, Andrew M. James, John F. Mulvey, Kurt Hoogewijs, Shujing Ding, Ian M. Fearnley, Marta Loureiro-López, Abigail A.I. Norman, Sabine Arndt, Amin Mottahedin, Olga Sauchanka, Richard C. Hartley, Thomas Krieg, and Michael P. Murphy

SUPPLEMENTAL INFORMATION

ND3 Cys39 in Complex I is Exposed During Mitochondrial Respiration

Nils Burger[1], Andrew M. James[1], John F. Mulvey[2], Kurt Hoogewijs[1,3,4], Shujing Ding[1], Ian M. Fearnley[1], Marta Loureiro-López[1], Abigail A. I. Norman[5], Sabine Arndt[1], Amin Mottahedin[1,2,6], Olga Sauchanka[2], Richard C. Hartley[5], Thomas Krieg[2], Michael P. Murphy[1,7,*]

[1]Medical Research Council-Mitochondrial Biology Unit, University of Cambridge, Cambridge CB2 0XY, UK

[2]Department of Medicine, University of Cambridge, Addenbrooke's Hospital, Cambridge CB2 0QQ, UK

[3]The Wellcome Trust Centre for Mitochondrial Research, Institute for Cell and Molecular Biosciences, Newcastle University, Newcastle upon Tyne, NE2 4HH, UK

[4]Medical Research Council-Laboratory of Molecular Biology, Cambridge, CB2 0QH, UK

[5]School of Chemistry, University of Glasgow, Glasgow G12 8QQ, UK

[6]Department of Physiology, Institute of Neuroscience and Physiology, Sahlgrenska Academy, University of Gothenburg, 405 30 Gothenburg, Sweden

[7]Lead Contact

[*] Correspondence: mpm@mrc-mbu.cam.ac.uk (M.P.M.)

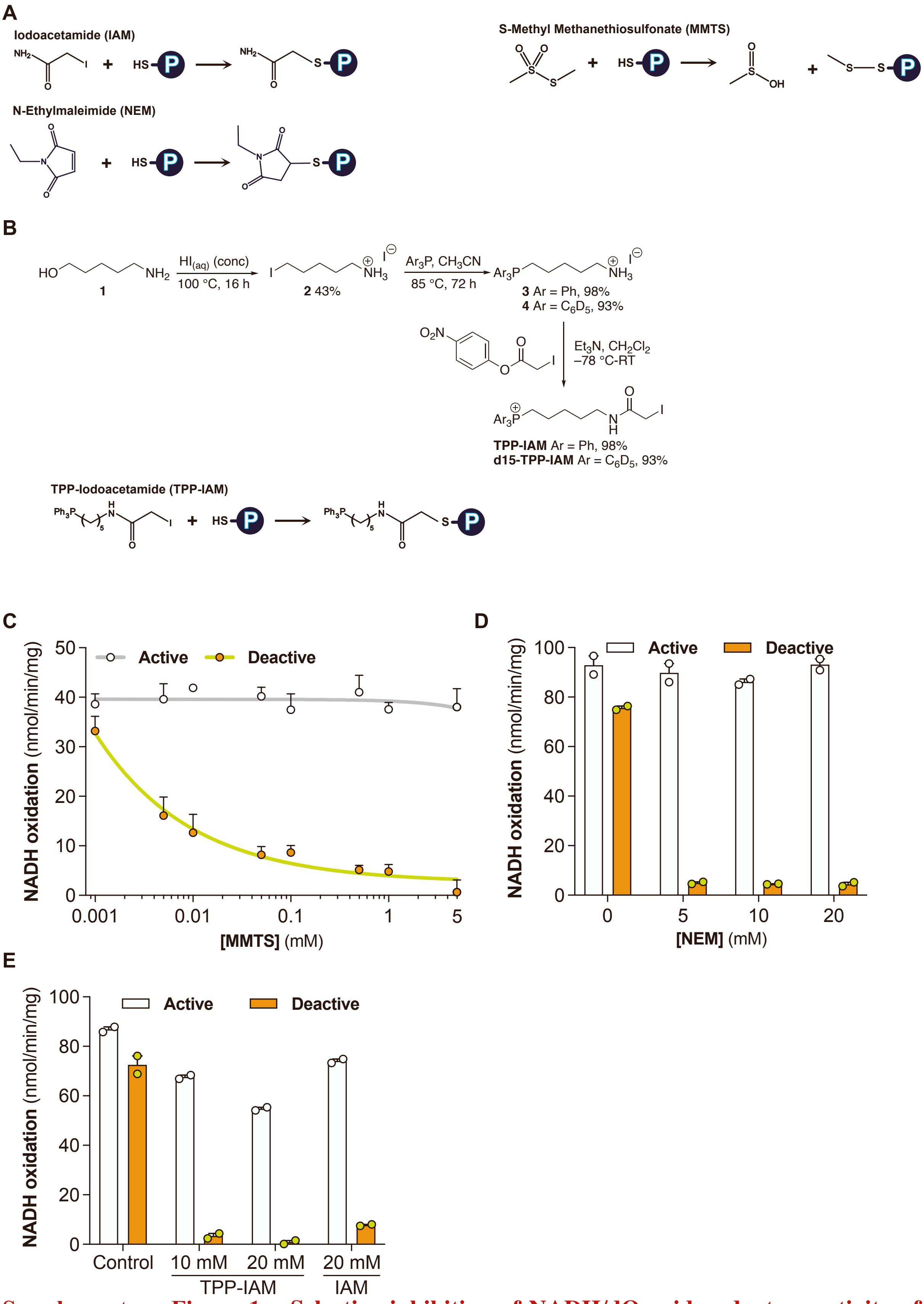

Supplementary Figure 1 – Selective inhibition of NADH/dQ oxidoreductase activity of catalytically deactive complex I by thiol reactants – Related to Figure 2

A Schematics of the reaction mechanism of the thiol reactive molecules iodoacetamide (IAM), *S*-methyl methanethiosulfonate (MMTS) and *N*-ethylmaleimide (NEM).

B Synthesis of TPP-Iodoacetamide (TPP-IAM) and schematics of its reaction mechanism.
C NADH/dQ oxidoreductase activity in catalytically active and deactive BHMMs upon labelling with increasing concentrations of MMTS for 5 min on ice. Data are presented as mean ± S.E.M. of three independent experiments. Each experiment was measured in triplicate.
D NADH/dQ oxidoreductase activity in catalytically active (kept on ice prior to activation with NADH) and deactive BHMMs upon labelling with increasing concentrations of NEM for 5 min on ice. Data are presented as mean ± range of two independently processed samples. Each sample was measured in duplicate.
E NADH/dQ oxidoreductase activity in catalytically active (kept on ice prior to activation with NADH) and deactive BHMMs upon labelling with different concentrations of TPP-IAM or IAM for 5 min at RT (active samples received 0.1 mM NADH during labelling). Data are presented as mean ± range of two independently processed samples. Each sample was measured in duplicate.

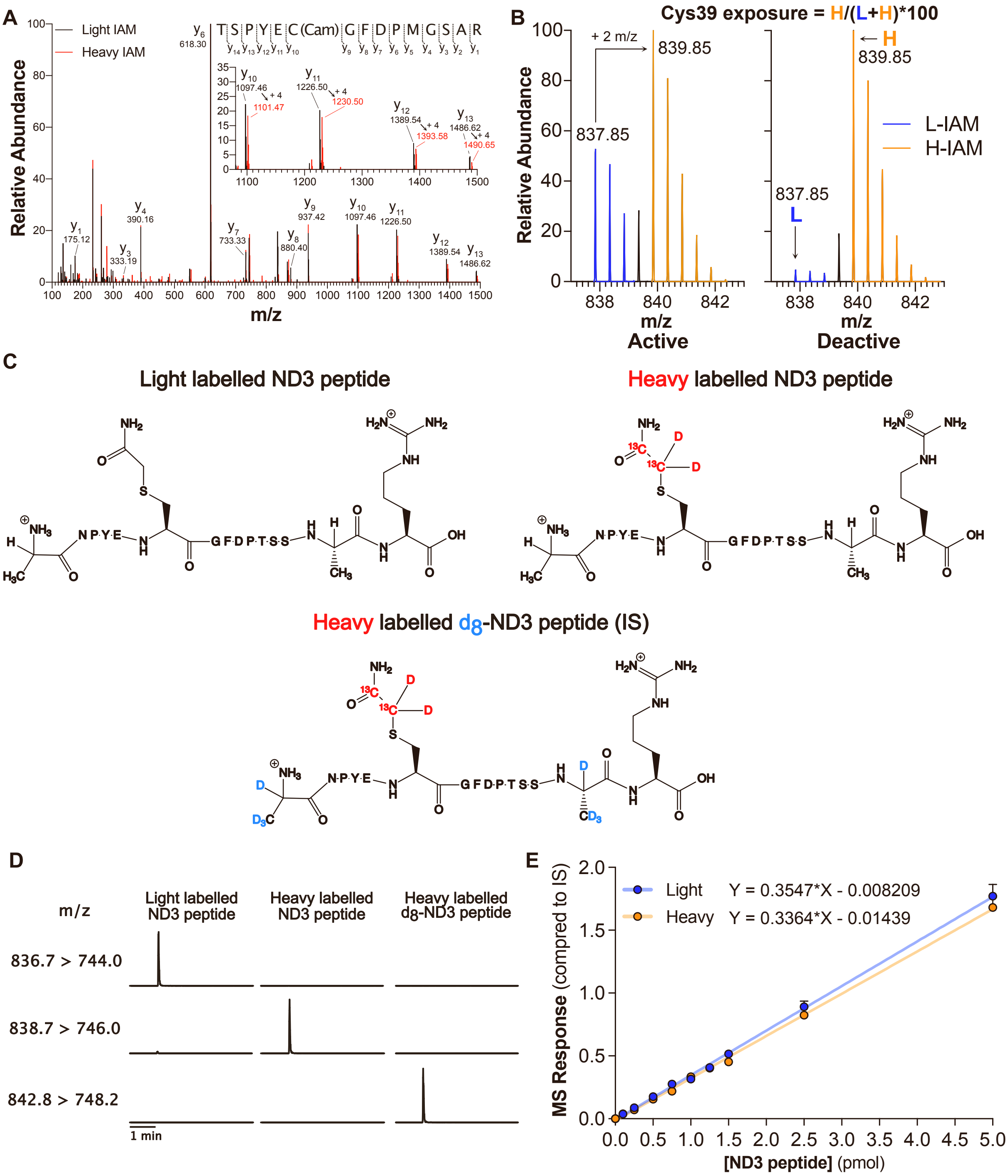

Supplementary Figure 2 – Detection and quantification of iodoacetamide labelled tryptic ND3 peptides by UTP and MRM – Related to Supplementary Results 1 and 2 and to STAR Methods

A Representative MS/MS fragmentation spectra for light (black) and heavy (red) labelled bovine tryptic ND3 peptide (precursors 837.85 m/z (light IAM labelled) and 839.85 m/z (heavy IAM labelled)) containing Cys39. The MS analysis was performed on a Q-Exactive Plus Orbitrap mass spectrometer. All detected peptide fragments are annotated. The mass shift for y-ions 10-13 labelled with heavy IAM are enhanced in the inset.

B Representative MS spectra of catalytically active and deactive BHMMs labelled with heavy IAM for 5 min on ice. Residual thiols were labelled with light IAM upon denaturation of the

proteins. The proportion of exposed Cys39 was calculated using the peak volume of the monoisotopic peak of light and heavy labelled ND3 peptides.
C Structure of light and heavy IAM labelled synthetic ND3 peptides (mouse/rat sequence) as well as a heavy isotope internal standard (IS) d_8-ND3 peptide.
D Representative MRM chromatograms from LC-MS/MS analyses showing the simultaneously measured m/z transitions of 0.5 pmol of light and heavy labelled ND3 peptide as well as the heavy labelled deuterated internal standard (IS). All chromatograms are normalized to the highest peak for each sample. Negligible bleed-through of light labelled ND3 peptide is detected in the transitions for heavy labelled ND3 peptide. The duration of both LC and MS detection were 5 min.
E Standard curve of light and heavy labelled ND3 peptides. The MS response of different peptide concentrations compared to 2.5 pmol internal standard (heavy labelled d_8-ND3 peptide) is shown. Data are presented as mean ± range of two replicates. The linear regression equations are shown.

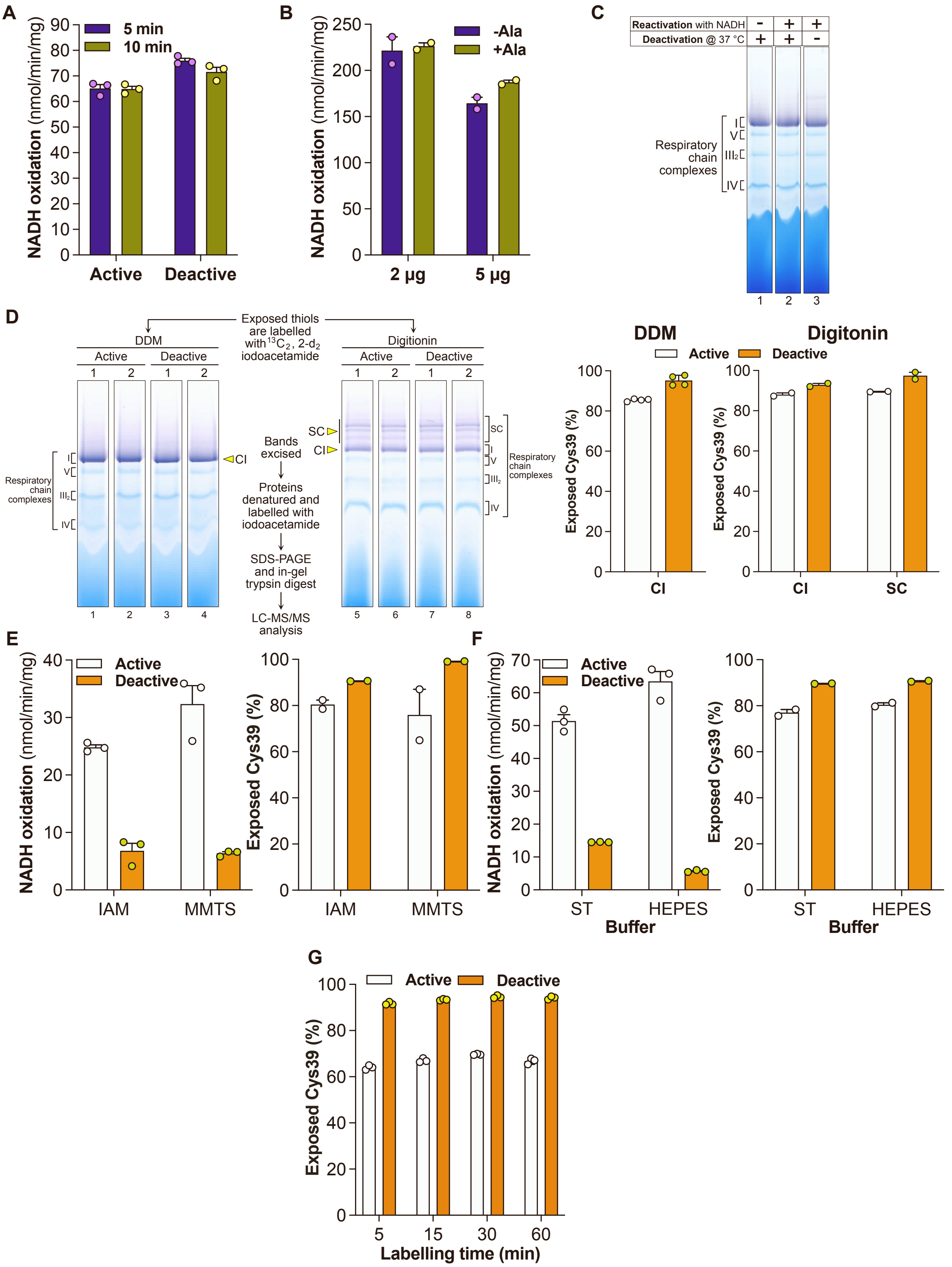

Supplementary Figure 3 – Cys39 is exposed by catalytically active complex I in BHMMs – Related to Figure 3

A NADH/dQ oxidoreductase activity in BHMMs that were deactivated for 20 min at 37 °C, followed by reactivation (catalytically active samples) with NADH for either 5 or 10 min on ice (catalytically deactive samples received buffer). Rates are presented as mean ± S.E.M. of three technical replicates each measured in duplicate.

B NADH/dQ oxidoreductase activity in BHMMs without any experimental pre-treatment, at 2 and 5 μg protein/well concentrations and if indicated in presence of 30 μg/ml alamethicin. Rates are presented as mean ± range of two independent samples, each measured in duplicate.
C BN-PAGE analysis of proteins solubilized with 1% DDM from BHMMs incubated at 37 °C or on ice ± reactivation with 1 mM NADH for 5 min on ice. In-gel staining of complex I flavin with nitrotetrazolium blue was performed.
D Proportion of exposed Cys39 by catalytically active and deactive complex I in BHMMs using a two-dimensional protein separation and differential labelling strategy. Exposed thiols were labelled with 20 mM IAM for 5 min on ice followed by BN-PAGE analysis upon solubilization with DDM or Digitonin. Proteins were denatured and a second labelling step was performed, followed by SDS-PAGE separation. Cys39 exposure is given for monomeric complex I (DDM); and for a combination of complex I in both monomeric form and as part of supercomplexes (Digitonin). Data are represented as mean ± S.E.M. (DDM: n=4) or mean ± range (Digitonin: n=2). SC are supercomplexes, CI is the monomeric complex I enzyme.
E NADH/dQ oxidoreductase activity and proportion of Cys39 exposure by catalytically active and deactive complex I in BHMMs in a combined sequential analysis upon labelling with 20 mM IAM or 0.5 mM MMTS for 5 min on ice. Data are presented as mean ± S.E.M. (complex I activity: n=3) or mean ± range (Cys39 exposure n=2).
F NADH/dQ oxidoreductase activity and proportion of Cys39 exposure by catalytically active and deactive complex I in BHMMs incubated in either ST (250 mM sucrose, 10 mM Tris pH 7.8 at 30 °C) or HEPES (20 mM HEPES pH 7.8 at 30 °C) buffers in a combined sequential analysis upon labelling with 20 mM IAM for 5 min on ice, followed by washing with 1 mM GSH. Data are presented as mean ± S.E.M. (complex I activity: n=3) or mean ± range (Cys39 exposure n=2).
G Cys39 exposure by catalytically active and deactive complex I in BHMMs upon labelling with 20 mM IAM for 5-60 min on ice. Data are presented as mean ± S.E.M. of three independently processed samples.

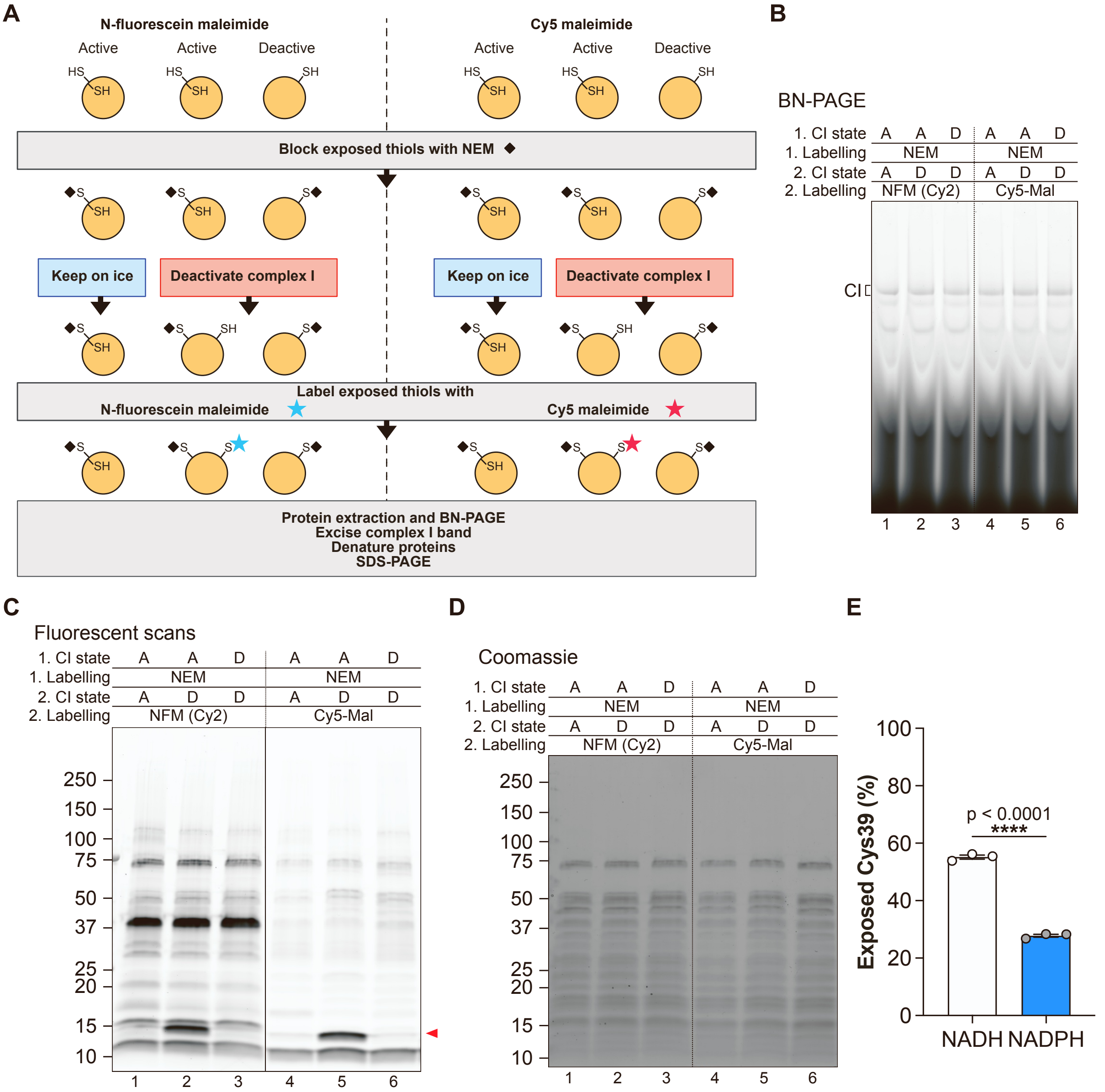

Supplementary Figure 4 - Cys39 exposure by complex I detected by fluorescent labelling – Related to Figure 4

A Schematic of the fluorescent labelling approach for Cys39. Two different fluorescent maleimides were used for labelling of occluded Cys39 within catalytically active complex I, followed by separation of labelled mitochondrial proteins via BN-PAGE and SDS-PAGE separation of subunits from the complex I band.

B-D Bovine heart mitochondrial membranes were subjected to the differential labelling with NEM and N-fluorescein maleimide/Cy5 maleimide as detailed in Figure S4A. Following the labelling proteins were extracted (with 1% DDM) and separated by BN-PAGE (B). The complex I band was excised, proteins were denatured and separated by SDS-PAGE, followed by fluorescence scanning of the gel (C) (composite: left: N-fluorescein maleimide (Cy2 filter); right: Cy5 maleimide (Cy5 filter); samples were loaded in adjacent lanes on one gel) and coomassie staining (D).

E Proportion of exposed Cys39 by bovine heart mitochondrial membranes that were activated for 30 min on ice in the presence of 1 mM NADH or NADPH followed by labelling with 20 mM IAM for 5 min on ice. Data are represented as mean ± S.E.M. of three independently processed samples. Data were evaluated using an unpaired Student's *t*-test.

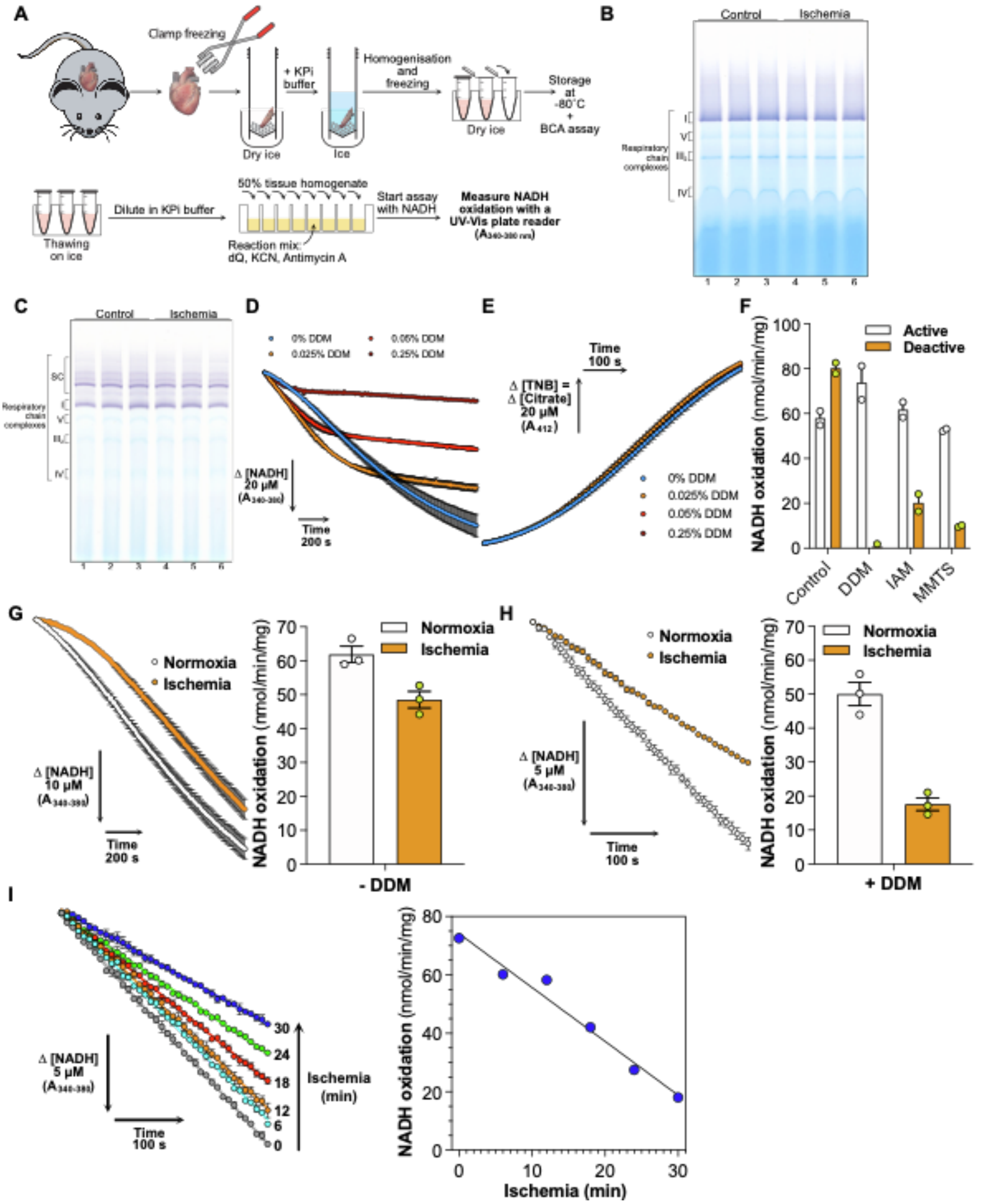

Supplementary Figure 5 – Setting up the NADH/dQ oxidoreductase activity assay for tissues – Related to Figure 6

A Schematic of the preparation of tissue homogenate as well as the subsequent analysis of complex I activity within the homogenate.

B+C BN page analysis of proteins solubilized with 1% DDM (B) or 8 g/g protein digitonin (C) from freshly prepared homogenate of normoxic or ischemic mouse hearts (n=3 individual hearts). In-gel staining of complex I flavin with nitrotetrazolium blue was performed.
D Representative traces of NADH oxidation by normoxic mouse heart homogenate upon addition of indicated amounts of DDM to the assay solution. Data are presented as mean ± S.E.M. of three individual wells within one experiment.
E Representative traces of DTNB conversion into TNB^{2-} by citrate synthase in normoxic mouse heart homogenate upon addition of indicated amounts of DDM to the assay solution. Data are presented as mean ± S.E.M. of three individual wells within one experiment.
F NADH/dQ oxidoreductase activity in catalytically active and deactive BHMMs upon labelling with 20 mM IAM or 0.5 mM MMTS for 5 min on ice or upon addition of 0.025% DDM during the assay. Data are presented as mean ± range of two independent experiments with each sample being measured in duplicate.
G Left: Representative traces of NADH oxidation by normoxic and ischemic mouse heart homogenate. Right: NADH/dQ oxidoreductase activity in normoxic and ischemic mouse heart homogenate. Traces of the NADH oxidation (before subtraction of rotenone control) are presented as mean ± S.E.M. of three individual hearts with each sample measured in duplicate. Complex I activity is quantified as rate of NADH oxidation and presented as mean ± S.E.M. of three individual hearts with samples being analyzed in two independent experiments and with each sample measured in duplicate.
H Left: Representative traces of NADH oxidation by normoxic and ischemic mouse heart homogenate in the presence of 0.025 % DDM. Right: NADH/dQ oxidoreductase activity in normoxic and ischemic mouse heart homogenate in the presence of 0.025 % DDM.
Traces of the NADH oxidation (before subtraction of rotenone control) are presented as mean ± S.E.M. of three individual hearts with each sample measured in duplicate. Complex I activity is quantified as rate of NADH oxidation and presented as mean ± S.E.M. of three individual hearts with samples being analyzed in two independent experiments and with each sample measured in duplicate.
I Representative traces of NADH oxidation in the presence of 0.025 % DDM by homogenate of mouse hearts, exposed to increasing periods of ischemia (left). Traces of the NADH oxidation (before subtraction of rotenone control) are presented as mean ± range of two individual wells. Quantified NADH/dQ oxidoreductase activity plotted against the period of ischemia (right). Each time point represents one individual heart, corresponding to the traces on the left.

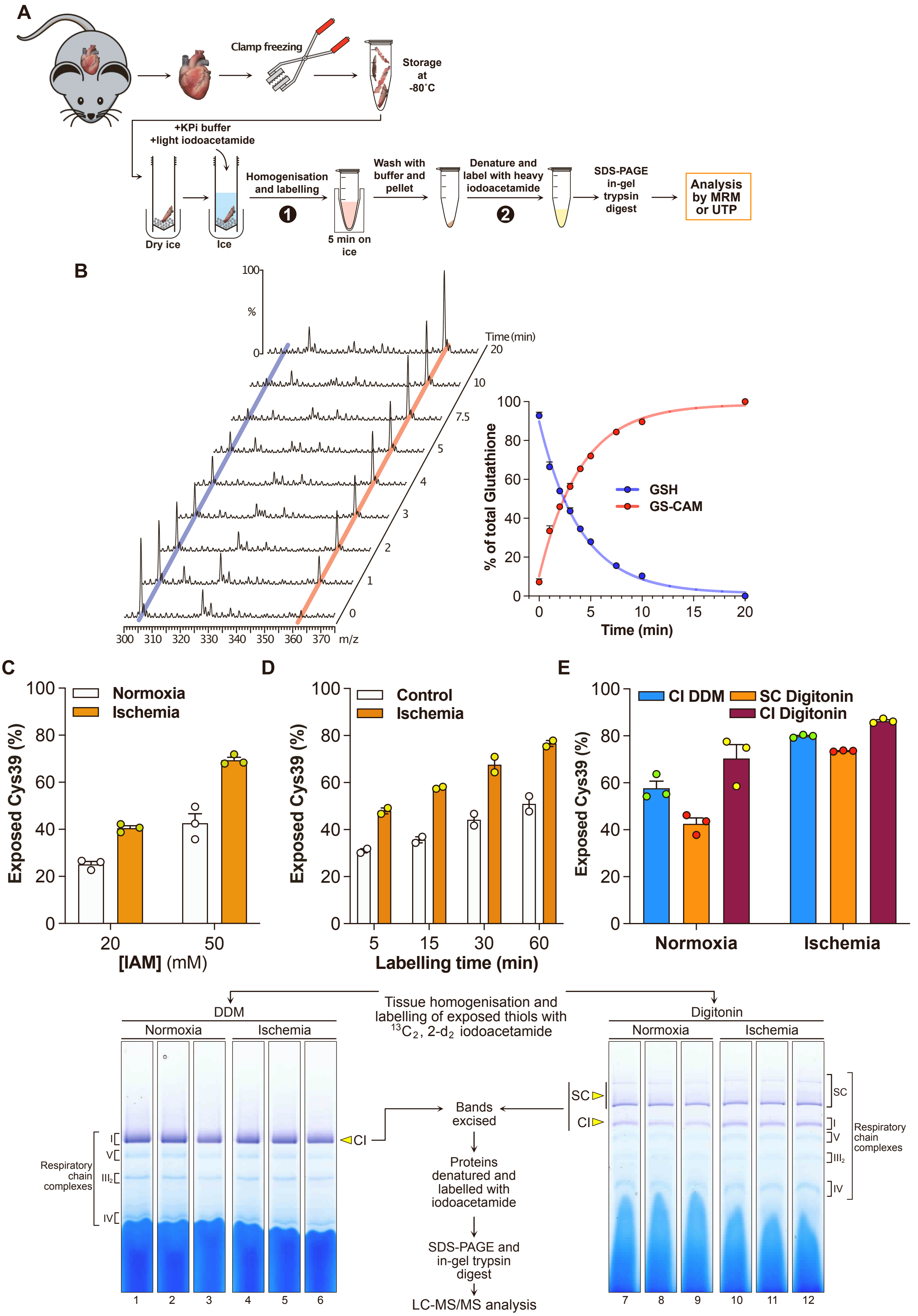

Supplementary Figure 6 – Method development to assess Cys39 exposure in tissues – Related to Figure 6

A Schematic of the differential labelling strategy to determine exposure of Cys39 by complex I in mouse heart via MRM or UTP
B Representative MS scans of GSH upon labelling with 20 mM IAM on ice for increasing time periods. The proportions of GSH and IAM labelled GS-CAM peaks are compared to the total peak intensity. Data are presented as mean ± range of two independent experiments.
C Cys39 exposure by complex I in normoxic and ischemic mouse heart, assessed by labelling of exposed thiols with 20 or 50 mM of IAM for 5 min on ice. Data are presented as mean ± S.E.M. of three individual hearts.
D Cys39 exposure by complex I in normoxic and ischemic mouse heart, assessed by labelling of exposed thiols with 20 mM of IAM for 5, 15, 30 and 60 min on ice. Data are presented as mean ± range of two individual hearts.
E Cys39 exposure by complex I in normoxic and ischemic mouse heart, assessed by labelling of exposed thiols with 20 mM of IAM using a two-dimensional protein separation and differential labelling strategy. Exposed thiols were labelled for 5 min on ice and proteins were analyzed by BN-PAGE upon solubilization with DDM (1% DDM; monomeric) and digitonin (8 g/g protein; monomeric and supercomplex forms), followed by a second labelling step and separation of proteins by SDS-PAGE. Cys39 exposure is given for monomeric complex I (DDM) and complex I in monomeric form and as part of supercomplexes (Digitonin). Data are represented as mean ± S.E.M. of three individual hearts.

Sup Table 1. Comparative analysis of cysteine residues within complex I - Related to Figures 3 and 4

Subunit	Cys residue (Mouse)	Mouse	Human	Bovine	Pig	Sheep	Rat	Chicken	Frog	Carp	Active (PDB: 6G2J) Surface Exposure (Å^2)	Deactive (PDB: 6G72) Surface Exposure (Å^2)	Presequence	FeS cluster	Disulfide predicted or modeled	MS detected	MS quantified	Active (% exposed)	Deactive (% exposed)	Peptide too short	Multiple Cys in peptide	Undetectable
ND1	301	X	-	X	X	X	X	X	-	-	0.98	2.24	-	-	-	-	-	n.d.	n.d.	-	-	-
ND2	275	X	-	-	-	-	X	-	-	-	0.21	1.61	-	-	-	n.e.	n.e.	n.e.	n.e.	n.e.	n.e.	n.e.
ND3	39	X	X	X	X	X	X	X	X	X	0.00	n.d.	-	-	-	X	X	61.9 ± 0.8[a]	92.0 ± 0.1[a]	-	-	-
ND4	200	X	-	X	X	X	X	-	-	X	0.00	0.00	-	-	-	-	-	n.d.	n.d.	-	-	x
	277	X	X	X	X	X	X	X	X	X	0.00	0.60	-	-	-	-	-	n.d.	n.d.	-	-	-
	329	X	X	X	X	X	X	X	X	X	0.00	0.00	-	-	-	-	-	n.d.	n.d.	-	-	-
ND4L	32	X	X	X	X	X	X	X	X	X	0.00	0.00	-	-	-	-	-	n.d.	n.d.	-	x	x
	69	X	X	X	X	X	X	X	X	X	1.61	1.93	-	-	-	-	-	n.d.	n.d.			x
	98	X	X	X	X	X	X	X	X	X	6.21	21.39	-	-	-	-	-	n.d.	n.d.			x
ND5	279	X	X	X	X	X	X	X	X	X	13.21	9.14	-	-	-	-	-	n.d.	n.d.	-	x	-
	291	X	X	X	X	X	X	X	-	X	1.60	2.72	-	-	-	-	-	n.d.	n.d.			-
	330	X	X	X	X	X	X	-	X	X	0.39	2.30	-	-	-	-	-	n.d.	n.d.	-	-	-
	342	X	X	X	X	X	X	X	X	X	0.19	0.00	-	-	-	-	-	n.d.	n.d.	-	-	-
	372	X	-	-	-	-	X	X	X	-	0.00	0.00	-	-	-	n.e.	n.e.	n.e.	n.e.	n.e.	n.e.	n.e.
	402	X	-	-	-	-	X	-	-	-	32.94	32.66	-	-	-	n.e.	n.e.	n.e.	n.e.	n.e.	n.e.	n.e.
ND6	16	X	-	-	-	-	X	-	-	-	1.47	2.04	-	-	-	n.e.	n.e.	n.e.	n.e.	n.e.	n.e.	n.e.
	40	X	X	X	X	X	X	X	X	X	8.83	11.38	-	-	-	-	-	n.d.	n.d.	-	-	x
	104	X	-	-	-	-	-	-	-	-	36.46	0.00	-	-	-	n.e.	n.e.	n.e.	n.e.	n.e.	n.e.	n.e.
	147	X	-	-	-	-	X	X	-	-	33.26	31.47	-	-	-	n.e.	n.e.	n.e.	n.e.	n.e.	n.e.	n.e.
NDUFA1	15	X	X	X	X	n.s.	X	X	n.s.	n.s.	5.92	8.72	-	-	-	-	-	n.d.	n.d.	-	-	-
NDUFA2	24	X	X	X	X	X	X	X	n.s.	n.s.	6.28	4.91	-	-	24-58	-	-	n.d.	n.d.	x	-	x
	58	X	X	X	X	X	X	X	n.s.	n.s.	2.70	3.90	-	-		-	-	n.d.	n.d.	-	-	-
NDUFA3																						
NDUFA5	17	X	X	X	X	X	X	-	n.s.	n.s.	21.35	57.89	-	-	-	X	X	42.2 ± 0.6[a]	45.8 ± 1.0[a]	-	-	-
NDUFA6																						
NDUFA7	55	X	X	X	X	X	X	X	n.s.	n.s.	0.00	4.37	-	-	-	X	X	1.5 ± 0.1[a]	2.1 ± 0.1[b]	-	-	-
NDUFA8	36	X	X	X	X	X	X	X	n.s.	n.s.	4.71	3.81	-	-	36-66	-	-	n.d.	n.d.	-	-	-
	46	X	X	X	X	X	X	X	n.s.	n.s.	0.00	0.19	-	-	46-56	-	-	n.d.	n.d.	x	-	x
	56	X	X	X	X	X	X	X	n.s.	n.s.	0.00	0.00	-	-		-	-	n.d.	n.d.	x	-	x
	66	X	X	X	X	X	X	X	n.s.	n.s.	23.55	22.73	-	-	36-66	X	X	0 ± 0[a]	6.4 ± 1.1[a]	-	-	-
	78	X	X	X	X	X	X	X	n.s.	n.s.	0.00	0.00	-	-	78-110	-	-	n.d.	n.d.	-	X	-
	88	X	X	X	X	X	X	X	n.s.	n.s.	0.19	1.95	-	-	88-100	-	-	n.d.	n.d.			-
	100	X	X	X	X	X	X	X	n.s.	n.s.	6.88	10.97	-	-		-	-	n.d.	n.d.	x	-	x
	110	X	X	X	X	X	X	X	n.s.	n.s.	9.36	15.82	-	-	78-110	X	X	6.1[c]	13.4 ± 9.9[b,d]	-	-	-
NDUFA9	29	X	-	-	-	-	X	-	n.s.	n.s.	n.e.	n.e.	X	-	-	n.e.	n.e.	n.e.	n.e.	n.e.	n.e.	n.e.
	86	X	X	X	X	X	X	X	n.s.	n.s.	0.39	1.32	-	-	-	X	X	10.5 ± 5.9[b]	n.d.	-	-	-
NDUFA10	34	X	X	-	X	-	X	-	n.s.	n.s.	n.e.	n.e.	X	-	-	n.e.	n.e.	n.e.	n.e.	n.e.	n.e.	n.e.
	67	X	X	X	X	X	X	-	n.s.	n.s.	0.62	2.63	-	-	-	X	X	2.9 ± 0.8[a]	4.4 ± 0.9[a]	-	-	-
	112	X	X	X	X	X	X	X	n.s.	n.s.	0.60	0.00	-	-	-	X	X	4.7 ± 0.2[a]	6.9 ± 0.7[a]	-	-	-
	183	X	X	X	X	X	X	X	n.s.	n.s.	0.00	0.00	-	-	-	X	-	4.9 ± 0.2[a]	n.d.	-	-	-
	253	X	X	X	X	X	X	-	n.s.	n.s.	0.00	0.00	-	-	-	X	-	0 ± 0[a]	n.d.	-	-	-
NDUFA11	18	X	X	X	X	X	X	X	n.s.	n.s.	14.60	11.40	-	-	18-74	X	X	11.2[c]	0.0[c]	-	-	-
	32	X	-	-	-	-	X	-	n.s.	n.s.	47.82	60.34	-	-	-	n.e.	n.e.	n.e.	n.e.	n.e.	n.e.	n.e.
	75	X	X	X	X	X	X	X	n.s.	n.s.	0.00	2.21	-	-	18-74	-	-	n.d.	n.d.	-	-	-
	95	X	X	X	X	X	X	X	n.s.	n.s.	28.74	21.18	-	-	95-115	X	X	0.7 ± 0.1[a,d]	1.0 ± 0.0[a,d]	-	-	-
	115	X	X	X	X	X	X	X	n.s.	n.s.	30.43	29.20	-	-		-	-	n.d.	n.d.	-	-	-
NDUFA12	92	X	-	X	X	X	X	-	n.s.	n.s.	4.65	5.09	-	-	-	X	X	6.1 ± 0.4[a]	5.9 ± 0.2[a]	-	-	-
NDUFA13																						
NDUFAB1	7	X	-	X	X	-	X	-	X	n.s.	n.e.	n.e.	X	-	-	n.e.	n.e.	n.e.	n.e.	n.e.	n.e.	n.e.
	9	X	-	X	X	-	X	X	X	n.s.	n.e.	n.e.	X	-	-	n.e.	n.e.	n.e.	n.e.	n.e.	n.e.	n.e.
	37	X	X	-	-	-	X	X	X	n.s.	n.e.	n.e.	X	-	-	n.e.	n.e.	n.e.	n.e.	n.e.	n.e.	n.e.
	65	X	X	X	X	X	X	X	X	n.s.	n.e.	n.e.	X	-	-	n.e.	n.e.	n.e.	n.e.	n.e.	n.e.	n.e.
	140	X	X	X	X	X	X	X	-	n.s.	13.57/ 23.21	13.58/ 23.19	-	-	-	X	X	19.8 ± 0.2[a]	20.6 ± 0.1[a]	-	-	-
NDUFB1	24	X	X	-	X	n.s.	-	X	n.s.	n.s.	35.50	32.80	-	-	-	n.e.	n.e.	n.e.	n.e.	n.e.	n.e.	n.e.
NDUFB2	21	X	-	-	-	-	X	-	n.s.	n.s.	n.e.	n.e.	X	-	-	n.e.	n.e.	n.e.	n.e.	n.e.	n.e.	n.e.
NDUFB3																						
NDUFB4																						
NDUFB5	20	X	-	-	-	-	-	n.s.	n.s.	n.s.	n.e.	n.e.	X	-	-	n.e.	n.e.	n.e.	n.e.	n.e.	n.e.	n.e.
NDUFB6																						
NDUFB7	59	X	X	X	X	X	X	X	n.s.	n.s.	7.88	6.52	-	-	59-90	-	-	n.d.	n.d.	-	-	-
	69	X	X	X	X	X	X	X	n.s.	n.s.	0.00	0.00	-	-	69-80	-	-	n.d.	n.d.	x	-	x
	80	X	X	X	X	X	X	X	n.s.	n.s.	0.56	4.01	-	-		X	X	1.2 ± 0.2[a]	0.9 ± 0.3[a]	-	-	-
	90	X	X	X	X	X	X	X	n.s.	n.s.	20.30	23.55	-	-	59-90	X	-	n.d.	13.7[c]	-	-	-
NDUFB8	130	X	X	X	X	X	X	-	-	n.s.	35.29	52.12	-	-	-	X	X	4.4 ± 0.4[a]	3.6 ± 0.1[a]	-	-	-
NDUFB9	4	X	-	-	-	-	X	-	n.s.	n.s.	6.18	10.55	-	-	-	n.e.	n.e.	n.e.	n.e.	n.e.	n.e.	n.e.
	31	X	X	X	X	X	X	X	n.s.	n.s.	0.00	0.00	-	-	-	X	X	1.5 ± 0.1[a]	2.5 ± 0.9[a]	-	-	-
	42	X	X	X	X	X	X	-	n.s.	n.s.	6.08	3.67	-	-	-	X	X	1.8 ± 0.2[a]	1.6 ± 0.1[a]	-	-	-
	96	X	X	X	X	X	X	-	n.s.	n.s.	0.58	1.43	-	-	-	-	-	n.d.	n.d.	x	-	x
	103	X	X	X	X	X	X	X	n.s.	n.s.	0.84	2.29	-	-	-	X	X	6.5 ± 0.9[a]	2.1 ± 0.5[a]	-	-	-
	77	X	X	X	X	X	X	X	X	n.s.	0.00	0.00	-	-	77-84	X	X	3.8 ± 1.0[a,d]	2.8 ± 0.1[a,d]	-	-	-
	84	X	X	X	X	X	X	X	X	n.s.	12.51	19.57	-	-		-	-	n.d.	n.d.	-	-	-

NDUFB10	113	X	X	X	X	X	X	X	n.s.	n.s.	0.65	5.65	-	-	113-125	-	-	n.d.	n.d.	x	-	x
	125	X	X	X	X	X	X	X	n.s.	n.s.	0.00	0.00	-	-		-	-	n.d.	n.d.	x	-	x
	155	X	X	X	X	X	X	X	n.s.	n.s.	1.13	4.24	-	-	-	-	-	n.d.	n.d.	x	-	x
NDUFB11	12	X	-	-	-	-	-	n.s.	n.s.	n.s.	n.e.	n.e.	X	-	-	n.e.	n.e.	n.e.	n.e.	n.e.	n.e.	n.e.
NDUFC1	22	X	-	-	-	n.s.	X	n.s.	n.s.	n.s.	n.e.	n.e.	X	-	-	n.e.	n.e.	n.e.	n.e.	n.e.	n.e.	n.e.
NDUFC2	40	X	X	X	X	X	X	-	n.s.	n.s.	32.38	37.58	-	-	-	X	X	0 ± 0[a]	0 ± 0[b]	-	-	-
NDUFS1	53	X	X	X	X	X	X	X	n.s.	n.s.	0.00	1.29	-	-	-	-	-	n.d.	n.d.	-	-	-
	64	X	X	X	X	X	X	X	n.s.	n.s.	3.88	2.23	-	X	-	-	-	n.d.	n.d.	x	-	x
	75	X	X	X	X	X	X	X	n.s.	n.s.	1.16	1.51	-	X	-	X	-	7.0[c]	n.d.	-	-	-
	78	X	X	X	X	X	X	X	n.s.	n.s.	4.75	2.43	-	X	-	X	X	6.0 ± 1.0[a]	11.9 ± 2.0[a]	-	-	-
	92	X	X	X	X	X	X	X	n.s.	n.s.	0.58	1.35	-	X	-	X	X	6.1 ± 0.5[a]	14.5 ± 5.0[a]	-	-	-
	128	X	X	X	X	X	X	X	n.s.	n.s.	11.14	8.52	-	X	-	X	-	0 ± 0[a]	n.d.	-	X	-
	131	X	X	X	X	X	X	X	n.s.	n.s.	13.91	10.23	-	X	-	X	-		n.d.			-
	137	X	X	X	X	X	X	X	n.s.	n.s.	12.81	12.25	-	X	-	X	-		n.d.			-
	176	X	X	X	X	X	X	X	n.s.	n.s.	14.64	14.03	-	X	-	-	-	n.d.	n.d.	x	X	x
	179	X	X	X	X	X	X	X	n.s.	n.s.	11.68	10.91	-	X	-	-	-	n.d.	n.d.			x
	182	X	X	X	X	X	X	X	n.s.	n.s.	15.97	17.95	-	X	-	-	-	n.d.	n.d.	x	-	x
	226	X	X	X	X	X	X	X	n.s.	n.s.	17.88	16.76	-	X	-	X	X	2.3 ± 0.3[a]	2.3 ± 0.9[a]	-	-	-
	367	X	X	X	X	X	X	X	n.s.	n.s.	0.38	0.53	-	-	-	X	X	4.4 ± 0.7[a]	4.9 ± 1.0[a]	-	-	-
	463	X	-	-	-	-	-	X	n.s.	n.s.	28.93	21.93	-	-	-	n.e.	n.e.	n.e.	n.e.	n.e.	n.e.	n.e.
	554	X	X	X	X	X	X	X	n.s.	n.s.	35.93	30.31	-	-	-	X	X	4.7 ± 0.6[a]	7.2 ± 1.5[a]	-	-	-
	564	X	X	X	X	X	X	X	n.s.	n.s.	0.44	0.44	-	-	-	X	X	3.3 ± 0.6[b]	3.8 ± 0.9[a]	-	-	-
	710	X	X	X	X	X	X	X	n.s.	n.s.	0.21	0.21	-	-	-	-	-	n.d.	n.d.	x	-	x
	727	X	X	X	X	X	X	X	n.s.	n.s.	n.e.	n.e.	-	-	-	X	X	17.1 ± 0.9[a]	15.4 ± 0.1[a]	-	-	-
NDUFS2	9	X	-	-	-	-	-	-	n.s.	n.s.	n.e.	n.e.	X	-	-	n.e.	n.e.	n.e.	n.e.	n.e.	n.e.	n.e.
	109	X	X	X	X	X	X	X	n.s.	n.s.	1.23	1.96	-	-	-	-	-	n.d.	n.d.	-	-	-
	146	X	X	X	X	X	X	X	n.s.	n.s.	1.77	1.74	-	-	-	X	X	26.7 ± 7.0[a]	44.5 ± 18[a]	-	-	-
	326	X	X	X	X	X	X	X	n.s.	n.s.	0.00	0.19	-	-	-	-	-	n.d.	n.d.	x	-	x
	332	X	X	X	X	X	X	X	n.s.	n.s.	0.00	1.36	-	-	-	-	-	n.d.	n.d.	x	-	x
	347	X	X	X	X	X	X	X	n.s.	n.s.	0.34	3.28	-	-	-	X	X	1.7 ± 0.1[a]	1.9 ± 0.2[a]	-	-	-
	422	X	X	X	X	X	X	X	n.s.	n.s.	0.00	0.57	-	-	-	-	-	n.d.	n.d.	x	-	x
NDUFS3	10	X	-	-	-	-	X	-	n.e.	n.s.	n.e.	n.e.	X	-	-	n.e.	n.e.	n.e.	n.e.	n.e.	n.e.	n.e.
	79	X	X	X	X	X	X	X	X	n.s.	18.73	14.51	-	-	-	-	-	n.d.	n.d.	-	X	-
	86	X	X	X	-	X	X	-	-	n.s.	1.40	2.86	-	-	-	-	-	n.d.	n.d.			-
NDUFS4	28	X	-	-	-	-	X	-	n.s.	n.s.	n.e.	n.e.	X	-	-	n.e.	n.e.	n.e.	n.e.	n.e.	n.e.	n.e.
NDUFS5	33	X	X	X	X	X	X	X	n.s.	n.s.	0.41	0.00	-	-	33-66	-	-	n.d.	n.d.	x	-	x
	43	X	X	X	X	X	X	X	n.s.	n.s.	3.67	1.87	-	-	43-56	X	-	n.d.	0 ± 0[b]	-	-	-
	56	X	X	X	X	X	X	X	n.s.	n.s.	0.00	0.00	-	-		-	-	n.d.	n.d.	x	-	x
	66	X	X	X	X	X	X	X	n.s.	n.s.	2.79	3.20	-	-	33-66	-	-	n.d.	n.d.	x	-	x
NDUFS6	79	X	X	X	X	n.s.	X	X	n.s.	n.s.	1.31	3.22	-	-	-	X	X	1.4 ± 0.1[a]	1.6 ± 0.4[a]	-	-	-
	104	X	X	X	X	n.s.	X	-	n.s.	n.s.	0.00	0.00	-	-	-	X	X	0 ± 0[a]	1.9 ± 1.1[a]	-	X	-
	107	X	X	X	X	n.s.	X	-	n.s.	n.s.	2.90	4.28	-	-	-	X	X					-
NDUFS7	99	X	X	X	X	X	X	X	n.s.	n.s.	16.20	17.11	-	X	-	-	-	n.d.	n.d.	-	X	-
	100	X	X	X	X	X	X	X	n.s.	n.s.	7.43	5.86	-	X	-	-	-	n.d.	n.d.			-
	164	X	X	X	X	X	X	X	n.s.	n.s.	23.81	21.70	-	X	-	X	-	0.0[c]	n.d.	-	-	-
	181	X	X	X	X	X	X	X	n.s.	n.s.	0.00	2.17	-	-	-	-	-	n.d.	n.d.	x	-	x
	194	X	X	X	X	X	X	X	n.s.	n.s.	7.22	6.37	-	X	-	X	X	1.0 ± 1.0[a]	8.6 ± 6.5[a]	-	-	-
NDUFS8	113	X	X	X	X	X	X	X	n.s.	n.s.	11.42	11.94	-	X	-	-	-	n.d.	n.d.	X	X	x
	116	X	X	X	X	X	X	X	n.s.	n.s.	10.57	9.96	-	X	-	-	-	n.d.	n.d.			x
	119	X	X	X	X	X	X	X	n.s.	n.s.	13.44	12.00	-	X	-	X	X	2.0 ± 0.2[a]	3.4 ± 1.0[a]	-	X	-
	123	X	X	X	X	X	X	X	n.s.	n.s.	14.36	14.19	-	X	-	X	X			-		-
	152	X	X	X	X	X	X	X	n.s.	n.s.	7.87	8.30	-	X	-	-	-	n.d.	n.d.	-	X	x
	155	X	X	X	X	X	X	X	n.s.	n.s.	11.56	10.32	-	X	-	-	-	n.d.	n.d.			x
	158	X	X	X	X	-	X	X	n.s.	n.s.	10.22	12.73	-	X	-	-	-	n.d.	n.d.			x
	162	X	X	X	X	-	X	X	n.s.	n.s.	13.13	10.64	-	X	-	-	-	n.d.	n.d.			x
NDUFV1	125	X	X	X	X	X	X	n.s.	n.s.	n.s.	3.61	5.75	-	-	-	X	X	2.0 ± 0.3[a]	3.3 ± 0.4[a]	-	-	-
	142	X	X	X	X	X	X	n.s.	n.s.	n.s.	0.00	0.00	-	-	-	X	X	2.5 ± 0.7[a]	6.8 ± 4.3[a]	-	-	-
	187	X	X	X	X	X	X	n.s.	n.s.	n.s.	15.76	4.29	-	-	-	X	X	6.0 ± 1.4[a]	12.8 ± 2.3[a]	-	-	-
	206	X	X	X	X	X	X	n.s.	n.s.	n.s.	0.00	0.00	-	-	-	X	X	2.2 ± 0.3[a]	1.5 ± 0.5[a]	-	-	-
	238	X	X	X	X	X	X	n.s.	n.s.	n.s.	0.21	0.00	-	-	-	X	X	0.4 ± 0.1[a]	0.6 ± 0.1[a]	-	X	-
	255	X	X	X	X	X	X	n.s.	n.s.	n.s.	0.00	0.00	-	-	-	X	X			-		-
	286	X	X	X	X	X	X	n.s.	n.s.	n.s.	0.00	0.00	-	-	-	X	X	1.1 ± 0.1[a]	1.4 ± 0.0[a]	-	-	-
	332	X	X	X	X	X	X	n.s.	n.s.	n.s.	1.62	3.70	-	-	-	-	-	n.d.	n.d.	-	-	-
	379	X	X	X	X	X	X	n.s.	n.s.	n.s.	10.97	10.30	-	X	-	-	-	n.d.	n.d.	-	X	-
	382	X	X	X	X	X	X	n.s.	n.s.	n.s.	11.81	10.62	-	X	-	-	-	n.d.	n.d.			-
	385	X	X	X	X	X	X	n.s.	n.s.	n.s.	13.38	12.89	-	X	-	-	-	n.d.	n.d.			-
	425	X	X	X	X	X	X	n.s.	n.s.	n.s.	15.37	14.40	-	X	-	X	X	2.2 ± 1.5[a]	2.4 ± 0.4[a]	-	-	-
NDUFV2	134	X	X	X	X	n.s.	X	X	n.s.	n.s.	1.75	0.79	-	X	-	-	-	n.d.	n.d.	-	X	-
	139	X	X	X	X	n.s.	X	X	n.s.	n.s.	2.46	1.87	-	X	-	-	-	n.d.	n.d.			-
	175	X	X	X	X	n.s.	X	X	n.s.	n.s.	0.38	0.16	-	X	-	-	-	n.d.	n.d.	-	X	-
	179	X	X	X	X	n.s.	X	X	n.s.	n.s.	1.92	1.34	-	X	-	-	-	n.d.	n.d.			-
	223	X	-	-	-	n.s.	X	-	n.s.	n.s.	0.19	0.77	-	-	-	n.e.	n.e.	n.e.	n.e.	n.e.	n.e.	n.e.
	224	X	X	X	X	n.s.	X	X	n.s.	n.s.	4.81	2.44	-	-	-	X	X	1.7 ± 0.5[a]	8.8 ± 3.8[a]	-	-	-
NDUFV3																						

Legend:

n.s. = no sequence
n.e. = not existing
n.d. = no data
X yes/positive
- no/negative

Exposure γ-sulfur atom (Å^2):
No exposure
Exposure <5 Å^2
Exposure >5 Å^2
5 Å^2 cutoff for solvent exposure
(James et al.,2018, Cell Reports 24, 1445-1455)

Remarks:
a N=3 ± S.E.M.
b N=2 ± range
c N=1
d Peptide with missed cleavage

Sup Table 2. Quantitation of the cysteine survey by MS - related to Sup Table 1

138 Cysteines found in mouse
116 (84.1 %) of these cysteines are conserved in bovine
114* (82.6/98.3 %) of these residues are found in the mature bovine enzyme

	Total (114*)	Cys detected	Cys quantified	Cys unquantified	Cys undetected	FeS cluster	Disulfide	Undetectable	Detectable
Total	100 % (114)	45.6 % (52)	37.7 % (43)	62.3 % (71)	54.4 % (62)	27.2 % (31)	22.8 % (26)	28.1 % (32)	72.9 % (82)
Cys detected	45.6 % (52)	-	-	12.7 % (9)	-	38.7 % (12)	30.8 % (8)	-	63.4 % (52)
Cys quantified	37.7 % (43)	82.7 % (43)	-	-	-	22.6 % (7)	23.1 % (6)	-	52.4 % (43)
Cys unquantified	62.3 % (71)	17.3 % (9)	-	-	-	77.4 % (24)	77.0 % (20)	-	47.6 % (39)
Cys undetected	54.4 % (62)	-	-	87.3 % (62)	-	61.3 % (19)	69.2 % (18)	100.0 % (32)	-
FeS cluster	27.2 % (31)	23.1 % (12)	16.3 % (7)	33.8 % (24)	30.7 % (19)	-	-	31.3 % (10)	25.6 % (21)
Disulfide	22.8 % (26)	15.4 % (8)	11.6 % (6)	28.2 % (20)	29.0 % (18)	-	-	31.3 % (10)	19.5 % (16)
Undetectable	28.1 % (32)	-	-	45.1 % (32)	51.6 % (32)	32.3 % (10)	38.5 % (10)	-	-
Detectable	72.9 % (82)	100 % (52)	100 % (43)	54.9 % (39)	48.3 % (30)	67.7 % (21)	61.5 % (16)	-	-

* NDUFAB1 is present in two copies within complex I but the cysteines are indistinguishable by mass spectrometry and therefore counted as a unique cysteine
