# Supplementary material for: ND3 Cys39 in complex I is exposed during mitochondrial respiration
Source: Cell Chem Biol. Author manuscript; Available in PMC 2022 May 18. (PMC9076552; doi:10.1016/j.chembiol.2021.10.010)
Supplement: Tables S1-S2 & Figures 1-6 [file EMS144932-supplement-Tables_S1_S2___Figures_1_6.pdf]

Cell Chemical Biology, Volume *29*

Supplemental information

ND3 Cys39 in complex I is exposed during mitochondrial respiration

Nils Burger, Andrew M. James, John F. Mulvey, Kurt Hoogewijs, Shujing Ding, Ian M. Fearnley, Marta Loureiro-López, Abigail A.I. Norman, Sabine Arndt, Amin Mottahedin, Olga Sauchanka, Richard C. Hartley, Thomas Krieg, and Michael P. Murphy

SUPPLEMENTAL INFORMATION

ND3 Cys39 in Complex I is Exposed During Mitochondrial Respiration

Nils Burger[1], Andrew M. James[1], John F. Mulvey[2], Kurt Hoogewijs[1,3,4], Shujing Ding[1], Ian M. Fearnley[1], Marta Loureiro-López[1], Abigail A. I. Norman[5], Sabine Arndt[1], Amin Mottahedin[1,2,6], Olga Sauchanka[2], Richard C. Hartley[5], Thomas Krieg[2], Michael P. Murphy[1,7,*]

[1]Medical Research Council-Mitochondrial Biology Unit, University of Cambridge, Cambridge CB2 0XY, UK

[2]Department of Medicine, University of Cambridge, Addenbrooke's Hospital, Cambridge CB2 0QQ, UK

[3]The Wellcome Trust Centre for Mitochondrial Research, Institute for Cell and Molecular Biosciences, Newcastle University, Newcastle upon Tyne, NE2 4HH, UK

[4]Medical Research Council-Laboratory of Molecular Biology, Cambridge, CB2 0QH, UK

[5]School of Chemistry, University of Glasgow, Glasgow G12 8QQ, UK

[6]Department of Physiology, Institute of Neuroscience and Physiology, Sahlgrenska Academy, University of Gothenburg, 405 30 Gothenburg, Sweden

[7]Lead Contact

[*] Correspondence: mpm@mrc-mbu.cam.ac.uk (M.P.M.)

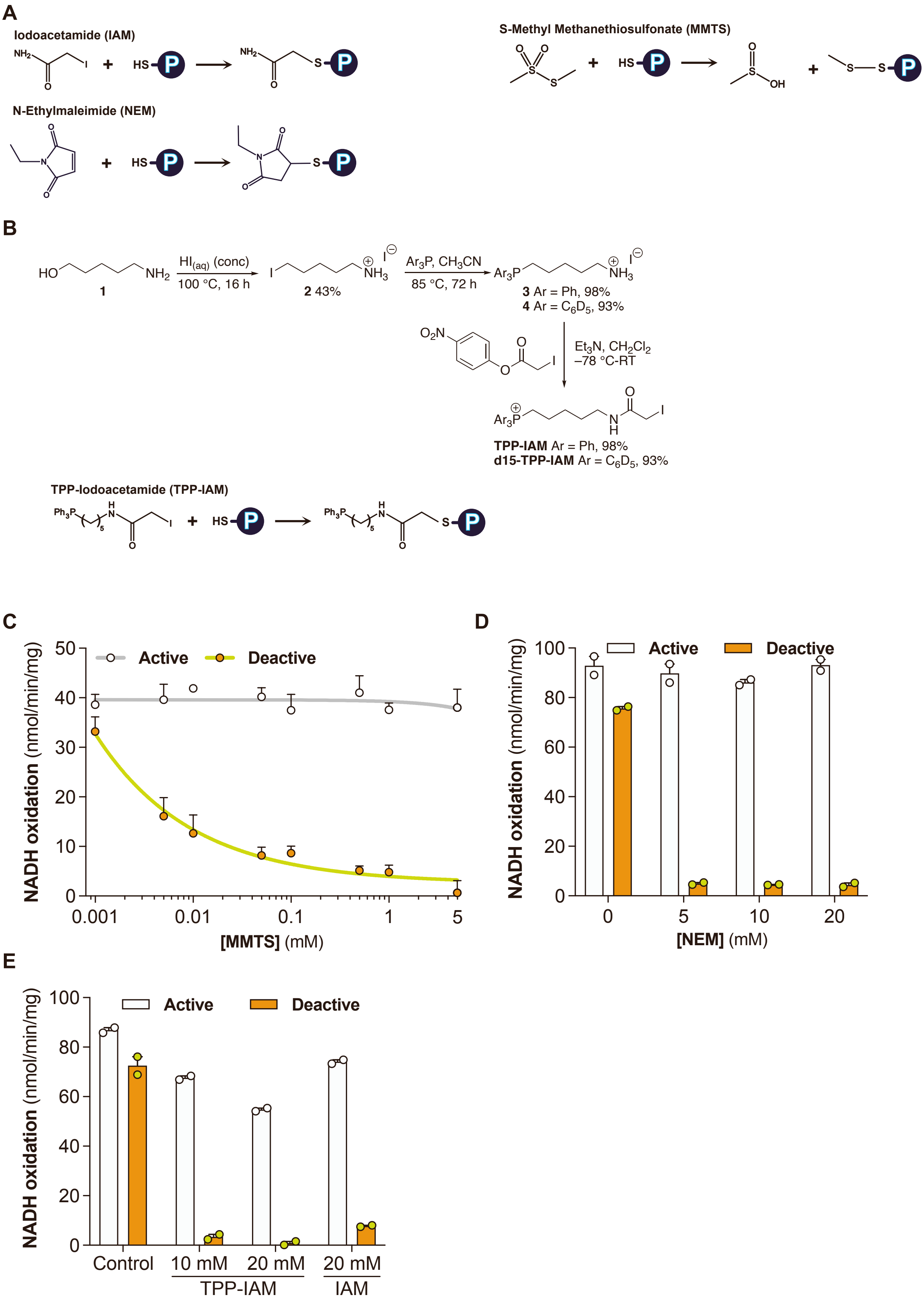

Supplementary Figure 1 – Selective inhibition of NADH/dQ oxidoreductase activity of catalytically deactive complex I by thiol reactants – Related to Figure 2

A Schematics of the reaction mechanism of the thiol reactive molecules iodoacetamide (IAM), *S*-methyl methanethiosulfonate (MMTS) and *N*-ethylmaleimide (NEM).

B Synthesis of TPP-Iodoacetamide (TPP-IAM) and schematics of its reaction mechanism.
C NADH/dQ oxidoreductase activity in catalytically active and deactive BHMMs upon labelling with increasing concentrations of MMTS for 5 min on ice. Data are presented as mean ± S.E.M. of three independent experiments. Each experiment was measured in triplicate.
D NADH/dQ oxidoreductase activity in catalytically active (kept on ice prior to activation with NADH) and deactive BHMMs upon labelling with increasing concentrations of NEM for 5 min on ice. Data are presented as mean ± range of two independently processed samples. Each sample was measured in duplicate.
E NADH/dQ oxidoreductase activity in catalytically active (kept on ice prior to activation with NADH) and deactive BHMMs upon labelling with different concentrations of TPP-IAM or IAM for 5 min at RT (active samples received 0.1 mM NADH during labelling). Data are presented as mean ± range of two independently processed samples. Each sample was measured in duplicate.

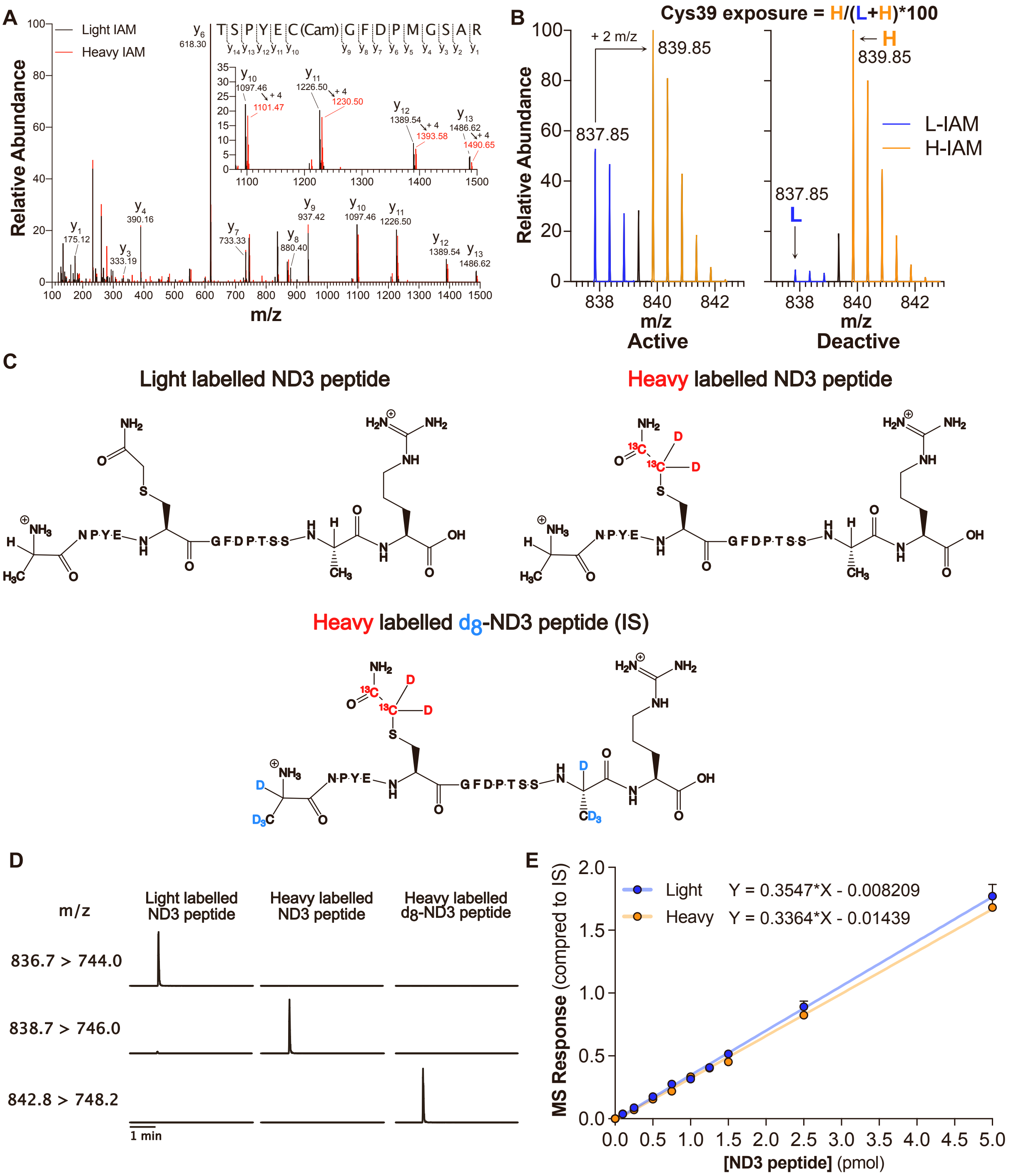

Supplementary Figure 2 – Detection and quantification of iodoacetamide labelled tryptic ND3 peptides by UTP and MRM – Related to Supplementary Results 1 and 2 and to STAR Methods

A Representative MS/MS fragmentation spectra for light (black) and heavy (red) labelled bovine tryptic ND3 peptide (precursors 837.85 m/z (light IAM labelled) and 839.85 m/z (heavy IAM labelled)) containing Cys39. The MS analysis was performed on a Q-Exactive Plus Orbitrap mass spectrometer. All detected peptide fragments are annotated. The mass shift for y-ions 10-13 labelled with heavy IAM are enhanced in the inset.

B Representative MS spectra of catalytically active and deactive BHMMs labelled with heavy IAM for 5 min on ice. Residual thiols were labelled with light IAM upon denaturation of the

proteins. The proportion of exposed Cys39 was calculated using the peak volume of the monoisotopic peak of light and heavy labelled ND3 peptides.

C Structure of light and heavy IAM labelled synthetic ND3 peptides (mouse/rat sequence) as well as a heavy isotope internal standard (IS) d_8-ND3 peptide.

D Representative MRM chromatograms from LC-MS/MS analyses showing the simultaneously measured m/z transitions of 0.5 pmol of light and heavy labelled ND3 peptide as well as the heavy labelled deuterated internal standard (IS). All chromatograms are normalized to the highest peak for each sample. Negligible bleed-through of light labelled ND3 peptide is detected in the transitions for heavy labelled ND3 peptide. The duration of both LC and MS detection were 5 min.

E Standard curve of light and heavy labelled ND3 peptides. The MS response of different peptide concentrations compared to 2.5 pmol internal standard (heavy labelled d_8-ND3 peptide) is shown. Data are presented as mean ± range of two replicates. The linear regression equations are shown.

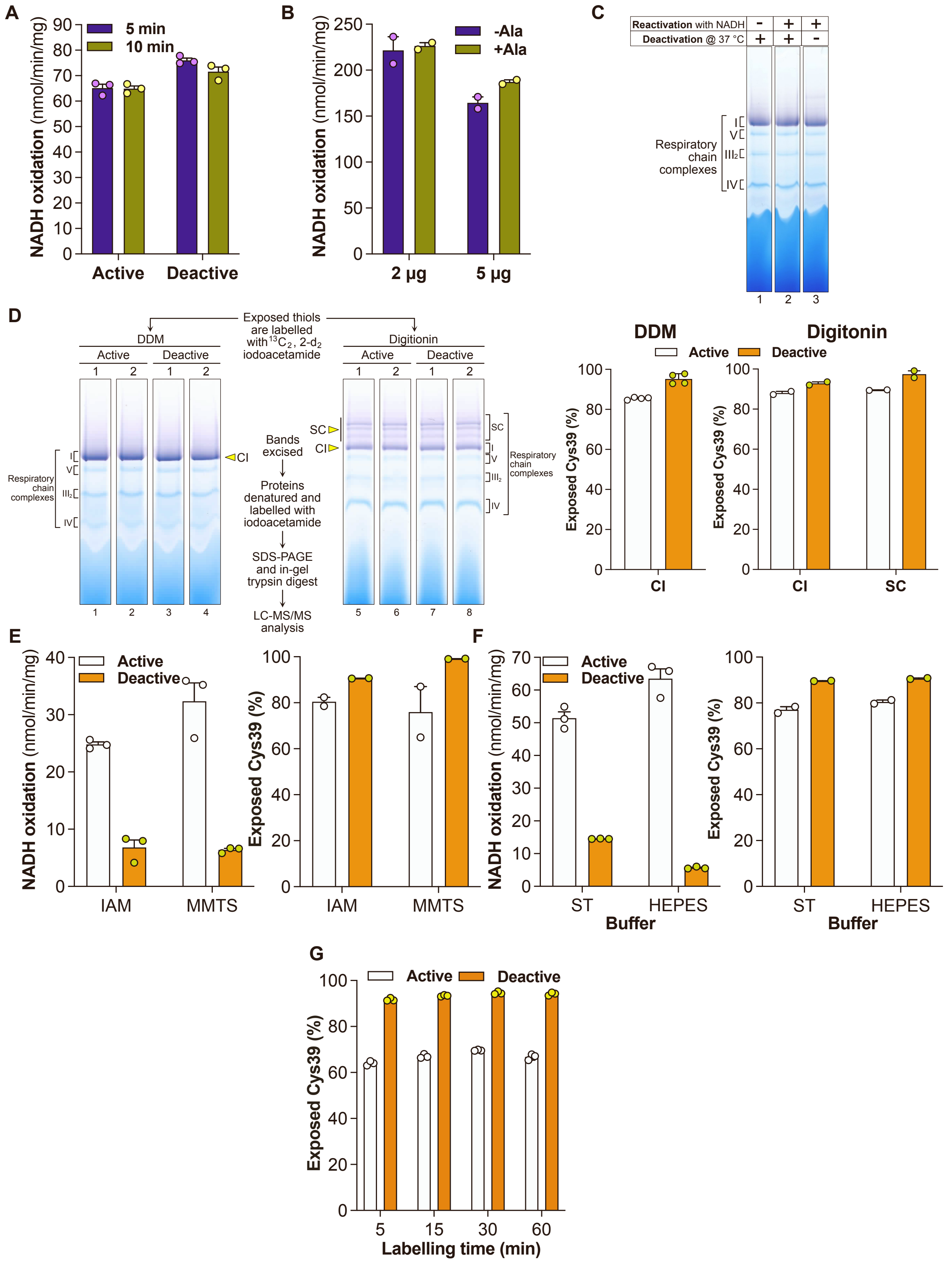

Supplementary Figure 3 – Cys39 is exposed by catalytically active complex I in BHMMs – Related to Figure 3

A NADH/dQ oxidoreductase activity in BHMMs that were deactivated for 20 min at 37 °C, followed by reactivation (catalytically active samples) with NADH for either 5 or 10 min on ice (catalytically deactive samples received buffer). Rates are presented as mean ± S.E.M. of three technical replicates each measured in duplicate.

B NADH/dQ oxidoreductase activity in BHMMs without any experimental pre-treatment, at 2 and 5 μg protein/well concentrations and if indicated in presence of 30 μg/ml alamethicin. Rates are presented as mean ± range of two independent samples, each measured in duplicate.
C BN-PAGE analysis of proteins solubilized with 1% DDM from BHMMs incubated at 37 °C or on ice ± reactivation with 1 mM NADH for 5 min on ice. In-gel staining of complex I flavin with nitrotetrazolium blue was performed.
D Proportion of exposed Cys39 by catalytically active and deactive complex I in BHMMs using a two-dimensional protein separation and differential labelling strategy. Exposed thiols were labelled with 20 mM IAM for 5 min on ice followed by BN-PAGE analysis upon solubilization with DDM or Digitonin. Proteins were denatured and a second labelling step was performed, followed by SDS-PAGE separation. Cys39 exposure is given for monomeric complex I (DDM); and for a combination of complex I in both monomeric form and as part of supercomplexes (Digitonin). Data are represented as mean ± S.E.M. (DDM: n=4) or mean ± range (Digitonin: n=2). SC are supercomplexes, CI is the monomeric complex I enzyme.
E NADH/dQ oxidoreductase activity and proportion of Cys39 exposure by catalytically active and deactive complex I in BHMMs in a combined sequential analysis upon labelling with 20 mM IAM or 0.5 mM MMTS for 5 min on ice. Data are presented as mean ± S.E.M. (complex I activity: n=3) or mean ± range (Cys39 exposure n=2).
F NADH/dQ oxidoreductase activity and proportion of Cys39 exposure by catalytically active and deactive complex I in BHMMs incubated in either ST (250 mM sucrose, 10 mM Tris pH 7.8 at 30 °C) or HEPES (20 mM HEPES pH 7.8 at 30 °C) buffers in a combined sequential analysis upon labelling with 20 mM IAM for 5 min on ice, followed by washing with 1 mM GSH. Data are presented as mean ± S.E.M. (complex I activity: n=3) or mean ± range (Cys39 exposure n=2).
G Cys39 exposure by catalytically active and deactive complex I in BHMMs upon labelling with 20 mM IAM for 5-60 min on ice. Data are presented as mean ± S.E.M. of three independently processed samples.

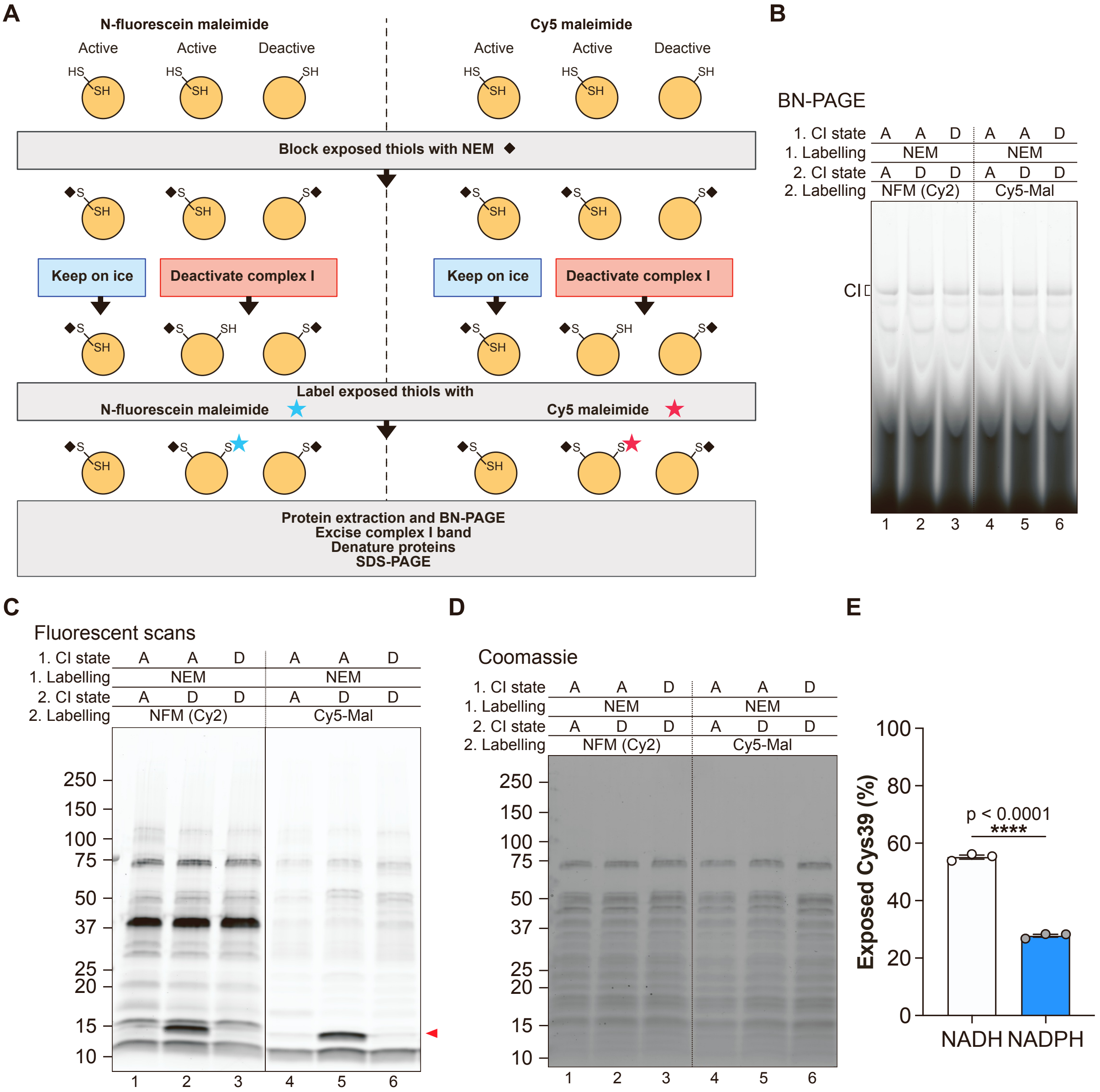

Supplementary Figure 4 - Cys39 exposure by complex I detected by fluorescent labelling – Related to Figure 4

A Schematic of the fluorescent labelling approach for Cys39. Two different fluorescent maleimides were used for labelling of occluded Cys39 within catalytically active complex I, followed by separation of labelled mitochondrial proteins via BN-PAGE and SDS-PAGE separation of subunits from the complex I band.

B-D Bovine heart mitochondrial membranes were subjected to the differential labelling with NEM and N-fluorescein maleimide/Cy5 maleimide as detailed in Figure S4A. Following the labelling proteins were extracted (with 1% DDM) and separated by BN-PAGE (B). The complex I band was excised, proteins were denatured and separated by SDS-PAGE, followed by fluorescence scanning of the gel (C) (composite: left: N-fluorescein maleimide (Cy2 filter); right: Cy5 maleimide (Cy5 filter); samples were loaded in adjacent lanes on one gel) and coomassie staining (D).

E Proportion of exposed Cys39 by bovine heart mitochondrial membranes that were activated for 30 min on ice in the presence of 1 mM NADH or NADPH followed by labelling with 20 mM IAM for 5 min on ice. Data are represented as mean ± S.E.M. of three independently processed samples. Data were evaluated using an unpaired Student's *t*-test.

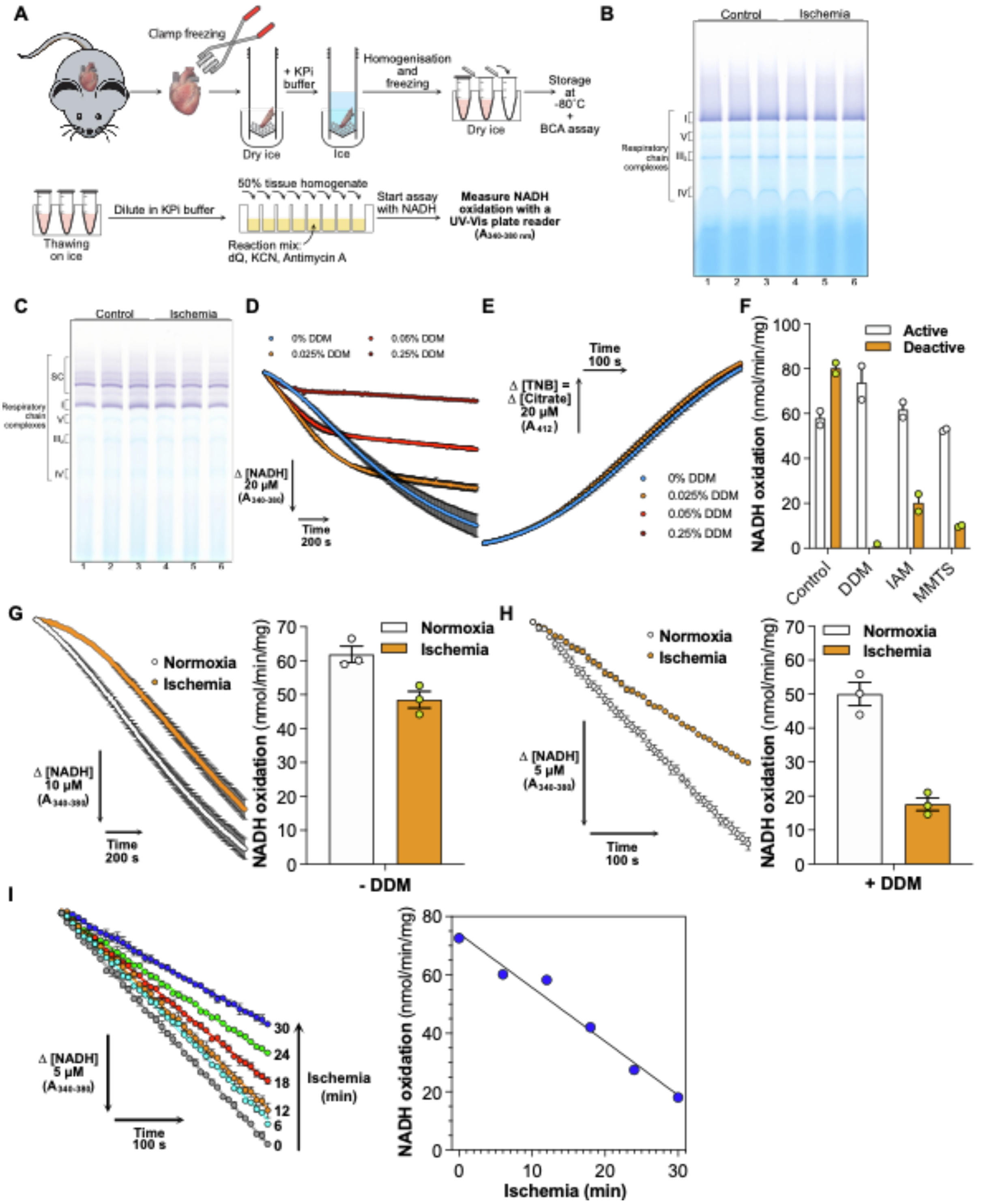

Supplementary Figure 5 – Setting up the NADH/dQ oxidoreductase activity assay for tissues – Related to Figure 6

A Schematic of the preparation of tissue homogenate as well as the subsequent analysis of complex I activity within the homogenate.

B+C BN page analysis of proteins solubilized with 1% DDM (B) or 8 g/g protein digitonin (C) from freshly prepared homogenate of normoxic or ischemic mouse hearts (n=3 individual hearts). In-gel staining of complex I flavin with nitrotetrazolium blue was performed.
D Representative traces of NADH oxidation by normoxic mouse heart homogenate upon addition of indicated amounts of DDM to the assay solution. Data are presented as mean ± S.E.M. of three individual wells within one experiment.
E Representative traces of DTNB conversion into TNB^{2-} by citrate synthase in normoxic mouse heart homogenate upon addition of indicated amounts of DDM to the assay solution. Data are presented as mean ± S.E.M. of three individual wells within one experiment.
F NADH/dQ oxidoreductase activity in catalytically active and deactive BHMMs upon labelling with 20 mM IAM or 0.5 mM MMTS for 5 min on ice or upon addition of 0.025% DDM during the assay. Data are presented as mean ± range of two independent experiments with each sample being measured in duplicate.
G Left: Representative traces of NADH oxidation by normoxic and ischemic mouse heart homogenate. Right: NADH/dQ oxidoreductase activity in normoxic and ischemic mouse heart homogenate. Traces of the NADH oxidation (before subtraction of rotenone control) are presented as mean ± S.E.M. of three individual hearts with each sample measured in duplicate. Complex I activity is quantified as rate of NADH oxidation and presented as mean ± S.E.M. of three individual hearts with samples being analyzed in two independent experiments and with each sample measured in duplicate.
H Left: Representative traces of NADH oxidation by normoxic and ischemic mouse heart homogenate in the presence of 0.025 % DDM. Right: NADH/dQ oxidoreductase activity in normoxic and ischemic mouse heart homogenate in the presence of 0.025 % DDM.
Traces of the NADH oxidation (before subtraction of rotenone control) are presented as mean ± S.E.M. of three individual hearts with each sample measured in duplicate. Complex I activity is quantified as rate of NADH oxidation and presented as mean ± S.E.M. of three individual hearts with samples being analyzed in two independent experiments and with each sample measured in duplicate.
I Representative traces of NADH oxidation in the presence of 0.025 % DDM by homogenate of mouse hearts, exposed to increasing periods of ischemia (left). Traces of the NADH oxidation (before subtraction of rotenone control) are presented as mean ± range of two individual wells. Quantified NADH/dQ oxidoreductase activity plotted against the period of ischemia (right). Each time point represents one individual heart, corresponding to the traces on the left.

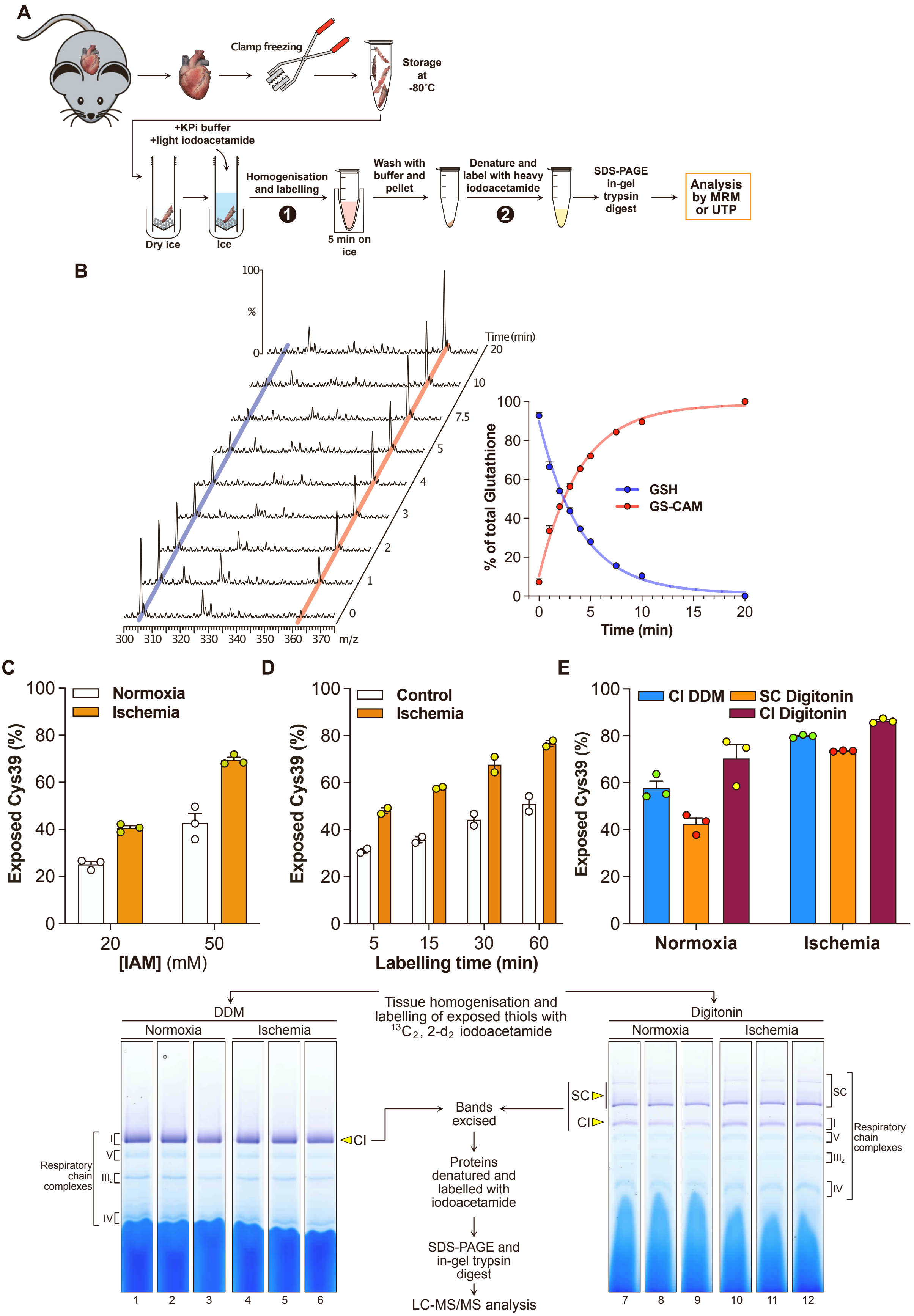

Supplementary Figure 6 – Method development to assess Cys39 exposure in tissues – Related to Figure 6

A Schematic of the differential labelling strategy to determine exposure of Cys39 by complex I in mouse heart via MRM or UTP

B Representative MS scans of GSH upon labelling with 20 mM IAM on ice for increasing time periods. The proportions of GSH and IAM labelled GS-CAM peaks are compared to the total peak intensity. Data are presented as mean ± range of two independent experiments.

C Cys39 exposure by complex I in normoxic and ischemic mouse heart, assessed by labelling of exposed thiols with 20 or 50 mM of IAM for 5 min on ice. Data are presented as mean ± S.E.M. of three individual hearts.

D Cys39 exposure by complex I in normoxic and ischemic mouse heart, assessed by labelling of exposed thiols with 20 mM of IAM for 5, 15, 30 and 60 min on ice. Data are presented as mean ± range of two individual hearts.

E Cys39 exposure by complex I in normoxic and ischemic mouse heart, assessed by labelling of exposed thiols with 20 mM of IAM using a two-dimensional protein separation and differential labelling strategy. Exposed thiols were labelled for 5 min on ice and proteins were analyzed by BN-PAGE upon solubilization with DDM (1% DDM; monomeric) and digitonin (8 g/g protein; monomeric and supercomplex forms), followed by a second labelling step and separation of proteins by SDS-PAGE. Cys39 exposure is given for monomeric complex I (DDM) and complex I in monomeric form and as part of supercomplexes (Digitonin). Data are represented as mean ± S.E.M. of three individual hearts.

Sup Table 1. Comparative analysis of cysteine residues within complex I - Related to Figures 3 and 4

Subunit	Cys residue (Mouse)	Mouse	Human	Bovine	Pig	Sheep	Rat	Chicken	Frog	Carp	Active (PDB: 6G2J) Surface Exposure (Å²)	Deactive (PDB: 6G72) Surface Exposure (Å²)	Presequence	FeS cluster	Disulfide predicted or modeled	MS detected	MS quantified	Active (% exposed)	Deactive (% exposed)	Peptide too short	Multiple Cys in peptide	Undetectable
ND1	301	X	-	X	X	X	X	X	-	-	0.98	2.24	-	-	-	-	-	n.d.	n.d.	-	-	-
ND2	275	X	-	-	-	-	X	-	-	-	0.21	1.61	-	-	-	n.e.	n.e.	n.e.	n.e.	n.e.	n.e.	n.e.
ND3	39	X	X	X	X	X	X	X	X	X	0.00	n.d.	-	-	-	X	X	61.9 ± 0.8[a]	92.0 ± 0.1[a]	-	-	-
ND4	200	X	-	X	X	X	X	-	-	X	0.00	0.00	-	-	-	-	-	n.d.	n.d.	-	-	x
	277	X	X	X	X	X	X	X	X	X	0.00	0.60	-	-	-	-	-	n.d.	n.d.	-	-	-
	329	X	X	X	X	X	X	X	X	X	0.00	0.00	-	-	-	-	-	n.d.	n.d.	-	-	-
ND4L	32	X	X	X	X	X	X	X	X	X	0.00	0.00	-	-	-	-	-	n.d.	n.d.	-	x	x
	69	X	X	X	X	X	X	X	X	X	1.61	1.93	-	-	-	-	-	n.d.	n.d.			x
	98	X	X	X	X	X	X	X	X	X	6.21	21.39	-	-	-	-	-	n.d.	n.d.			x
ND5	279	X	X	X	X	X	X	X	X	X	13.21	9.14	-	-	-	-	-	n.d.	n.d.	-	x	-
	291	X	X	X	X	X	X	X	-	X	1.60	2.72	-	-	-	-	-	n.d.	n.d.			-
	330	X	X	X	X	X	X	-	X	X	0.39	2.30	-	-	-	-	-	n.d.	n.d.	-	-	-
	342	X	X	X	X	X	X	X	X	X	0.19	0.00	-	-	-	-	-	n.d.	n.d.	-	-	-
	372	X	-	-	-	-	X	X	X	-	0.00	0.00	-	-	-	n.e.	n.e.	n.e.	n.e.	n.e.	n.e.	n.e.
	402	X	-	-	-	-	X	-	-	-	32.94	32.66	-	-	-	n.e.	n.e.	n.e.	n.e.	n.e.	n.e.	n.e.
ND6	16	X	-	-	-	-	X	-	-	-	1.47	2.04	-	-	-	n.e.	n.e.	n.e.	n.e.	n.e.	n.e.	n.e.
	40	X	X	X	X	X	X	X	X	X	8.83	11.38	-	-	-	-	-	n.d.	n.d.	-	-	x
	104	X	-	-	-	-	-	-	-	-	36.46	0.00	-	-	-	n.e.	n.e.	n.e.	n.e.	n.e.	n.e.	n.e.
	147	X	-	-	-	-	X	X	-	-	33.26	31.47	-	-	-	n.e.	n.e.	n.e.	n.e.	n.e.	n.e.	n.e.
NDUFA1	15	X	X	X	X	n.s.	X	X	n.s.	n.s.	5.92	8.72	-	-	-	-	-	n.d.	n.d.	-	-	-
NDUFA2	24	X	X	X	X	X	X	X	n.s.	n.s.	6.28	4.91	-	-	24-58	-	-	n.d.	n.d.	x	-	x
	58	X	X	X	X	X	X	X	n.s.	n.s.	2.70	3.90	-	-		-	-	n.d.	n.d.	-	-	-
NDUFA3																						
NDUFA5	17	X	X	X	X	X	X	-	n.s.	n.s.	21.35	57.89	-	-	-	X	X	42.2 ± 0.6[a]	45.8 ± 1.0[a]	-	-	-
NDUFA6																						
NDUFA7	55	X	X	X	X	X	X	X	n.s.	n.s.	0.00	4.37	-	-	-	X	X	1.5 ± 0.1[a]	2.1 ± 0.1[b]	-	-	-
NDUFA8	36	X	X	X	X	X	X	X	n.s.	n.s.	4.71	3.81	-	-	36-66	-	-	n.d.	n.d.	-	-	-
	46	X	X	X	X	X	X	X	n.s.	n.s.	0.00	0.19	-	-	46-56	-	-	n.d.	n.d.	x	-	x
	56	X	X	X	X	X	X	X	n.s.	n.s.	0.00	0.00	-	-		-	-	n.d.	n.d.	x	-	x
	66	X	X	X	X	X	X	X	n.s.	n.s.	23.55	22.73	-	-	36-66	X	X	0 ± 0[a]	6.4 ± 1.1[a]	-	-	-
	78	X	X	X	X	X	X	X	n.s.	n.s.	0.00	0.00	-	-	78-110	-	-	n.d.	n.d.	-	X	-
	88	X	X	X	X	X	X	X	n.s.	n.s.	0.19	1.95	-	-	88-100	-	-	n.d.	n.d.			-
	100	X	X	X	X	X	X	X	n.s.	n.s.	6.88	10.97	-	-		-	-	n.d.	n.d.	x	-	x
	110	X	X	X	X	X	X	X	n.s.	n.s.	9.36	15.82	-	-	78-110	X	X	6.1[c]	13.4 ± 9.9[b,d]	-	-	-
NDUFA9	29	X	-	-	-	-	X	-	n.s.	n.s.	n.e.	n.e.	X	-	-	n.e.	n.e.	n.e.	n.e.	n.e.	n.e.	n.e.
	86	X	X	X	X	X	X	X	n.s.	n.s.	0.39	1.32	-	-	-	X	X	10.5 ± 5.9[b]	n.d.	-	-	-
NDUFA10	34	X	X	-	X	-	X	-	n.s.	n.s.	n.e.	n.e.	X	-	-	n.e.	n.e.	n.e.	n.e.	n.e.	n.e.	n.e.
	67	X	X	X	X	X	X	-	n.s.	n.s.	0.62	2.63	-	-	-	X	X	2.9 ± 0.8[a]	4.4 ± 0.9[a]	-	-	-
	112	X	X	X	X	X	X	X	n.s.	n.s.	0.60	0.00	-	-	-	X	X	4.7 ± 0.2[a]	6.9 ± 0.7[a]	-	-	-
	183	X	X	X	X	X	X	X	n.s.	n.s.	0.00	0.00	-	-	-	X	-	4.9 ± 0.2[a]	n.d.	-	-	-
	253	X	X	X	X	X	X	-	n.s.	n.s.	0.00	0.00	-	-	-	X	-	0 ± 0[a]	n.d.	-	-	-
NDUFA11	18	X	X	X	X	X	X	X	n.s.	n.s.	14.60	11.40	-	-	18-74	X	X	11.2[c]	0.0[c]	-	-	-
	32	X	-	-	-	-	X	-	n.s.	n.s.	47.82	60.34	-	-	-	n.e.	n.e.	n.e.	n.e.	n.e.	n.e.	n.e.
	75	X	X	X	X	X	X	X	n.s.	n.s.	0.00	2.21	-	-	18-74	-	-	n.d.	n.d.	-	-	-
	95	X	X	X	X	X	X	X	n.s.	n.s.	28.74	21.18	-	-	95-115	X	X	0.7 ± 0.1[a,d]	1.0 ± 0.0[a,d]	-	-	-
	115	X	X	X	X	X	X	X	n.s.	n.s.	30.43	29.20	-	-		-	-	n.d.	n.d.	-	-	-
NDUFA12	92	X	-	X	X	X	X	-	n.s.	n.s.	4.65	5.09	-	-	-	X	X	6.1 ± 0.4[a]	5.9 ± 0.2[a]	-	-	-
NDUFA13																						
NDUFAB1	7	X	-	X	X	-	X	-	X	n.s.	n.e.	n.e.	X	-	-	n.e.	n.e.	n.e.	n.e.	n.e.	n.e.	n.e.
	9	X	-	X	X	-	X	X	X	n.s.	n.e.	n.e.	X	-	-	n.e.	n.e.	n.e.	n.e.	n.e.	n.e.	n.e.
	37	X	X	-	-	-	X	X	X	n.s.	n.e.	n.e.	X	-	-	n.e.	n.e.	n.e.	n.e.	n.e.	n.e.	n.e.
	65	X	X	X	X	X	X	X	X	n.s.	n.e.	n.e.	X	-	-	n.e.	n.e.	n.e.	n.e.	n.e.	n.e.	n.e.
	140	X	X	X	X	X	X	X	-	n.s.	13.57/ 23.21	13.58/ 23.19	-	-	-	X	X	19.8 ± 0.2[a]	20.6 ± 0.1[a]	-	-	-
NDUFB1	24	X	X	-	X	n.s.	-	X	n.s.	n.s.	35.50	32.80	-	-	-	n.e.	n.e.	n.e.	n.e.	n.e.	n.e.	n.e.
NDUFB2	21	X	-	-	-	-	X	-	n.s.	n.s.	n.e.	n.e.	X	-	-	n.e.	n.e.	n.e.	n.e.	n.e.	n.e.	n.e.
NDUFB3																						
NDUFB4																						
NDUFB5	20	X	-	-	-	-	-	n.s.	n.s.	n.s.	n.e.	n.e.	X	-	-	n.e.	n.e.	n.e.	n.e.	n.e.	n.e.	n.e.
NDUFB6																						
NDUFB7	59	X	X	X	X	X	X	X	n.s.	n.s.	7.88	6.52	-	-	59-90	-	-	n.d.	n.d.	-	-	-
	69	X	X	X	X	X	X	X	n.s.	n.s.	0.00	0.00	-	-	69-80	-	-	n.d.	n.d.	x	-	x
	80	X	X	X	X	X	X	X	n.s.	n.s.	0.56	4.01	-	-		X	X	1.2 ± 0.2[a]	0.9 ± 0.3[a]	-	-	-
	90	X	X	X	X	X	X	X	n.s.	n.s.	20.30	23.55	-	-	59-90	X	-	n.d.	13.7[c]	-	-	-
NDUFB8	130	X	X	X	X	X	X	-	-	n.s.	35.29	52.12	-	-	-	X	X	4.4 ± 0.4[a]	3.6 ± 0.1[a]	-	-	-
NDUFB9	4	X	-	-	-	-	X	-	n.s.	n.s.	6.18	10.55	-	-	-	n.e.	n.e.	n.e.	n.e.	n.e.	n.e.	n.e.
	31	X	X	X	X	X	X	X	n.s.	n.s.	0.00	0.00	-	-	-	X	X	1.5 ± 0.1[a]	2.5 ± 0.9[a]	-	-	-
	42	X	X	X	X	X	X	-	n.s.	n.s.	6.08	3.67	-	-	-	X	X	1.8 ± 0.2[a]	1.6 ± 0.1[a]	-	-	-
	96	X	X	X	X	X	X	-	n.s.	n.s.	0.58	1.43	-	-	-	-	-	n.d.	n.d.	x	-	x
	103	X	X	X	X	X	X	X	n.s.	n.s.	0.84	2.29	-	-	-	X	X	6.5 ± 0.9[a]	2.1 ± 0.5[a]	-	-	-
	77	X	X	X	X	X	X	X	X	n.s.	0.00	0.00	-	-	77-84	X	X	3.8 ± 1.0[a,d]	2.8 ± 0.1[a,d]	-	-	-
	84	X	X	X	X	X	X	X	X	n.s.	12.51	19.57	-	-		-	-	n.d.	n.d.	-	-	-

NDUFB10	113	X	X	X	X	X	X	X	n.s.	n.s.	0.65	5.65	-	-	113-125	-	-	n.d.	n.d.	x	-	x
	125	X	X	X	X	X	X	X	n.s.	n.s.	0.00	0.00	-	-		-	-	n.d.	n.d.	x	-	x
	155	X	X	X	X	X	X	X	n.s.	n.s.	1.13	4.24	-	-	-	-	-	n.d.	n.d.	x	-	x
NDUFB11	12	X	-	-	-	-	-	n.s.	n.s.	n.s.	n.e.	n.e.	X	-	-	n.e.	n.e.	n.e.	n.e.	n.e.	n.e.	n.e.
NDUFC1	22	X	-	-	-	n.s.	X	n.s.	n.s.	n.s.	n.e.	n.e.	X	-	-	n.e.	n.e.	n.e.	n.e.	n.e.	n.e.	n.e.
NDUFC2	40	X	X	X	X	X	X	-	n.s.	n.s.	32.38	37.58	-	-	-	X	X	0 ± 0[a]	0 ± 0[b]	-	-	-
NDUFS1	53	X	X	X	X	X	X	X	n.s.	n.s.	0.00	1.29	-	-	-	-	-	n.d.	n.d.	-	-	-
	64	X	X	X	X	X	X	X	n.s.	n.s.	3.88	2.23	-	X	-	-	-	n.d.	n.d.	x	-	x
	75	X	X	X	X	X	X	X	n.s.	n.s.	1.16	1.51	-	X	-	X	-	7.0[c]	n.d.	-	-	-
	78	X	X	X	X	X	X	X	n.s.	n.s.	4.75	2.43	-	X	-	X	X	6.0 ± 1.0[a]	11.9 ± 2.0[a]	-	-	-
	92	X	X	X	X	X	X	X	n.s.	n.s.	0.58	1.35	-	X	-	X	X	6.1 ± 0.5[a]	14.5 ± 5.0[a]	-	-	-
	128	X	X	X	X	X	X	X	n.s.	n.s.	11.14	8.52	-	X	-	X	-	0 ± 0[a]	n.d.	-	X	-
	131	X	X	X	X	X	X	X	n.s.	n.s.	13.91	10.23	-	X	-	X	-		n.d.			-
	137	X	X	X	X	X	X	X	n.s.	n.s.	12.81	12.25	-	X	-	X	-		n.d.			-
	176	X	X	X	X	X	X	X	n.s.	n.s.	14.64	14.03	-	X	-	-	-	n.d.	n.d.	x	X	x
	179	X	X	X	X	X	X	X	n.s.	n.s.	11.68	10.91	-	X	-	-	-	n.d.	n.d.			x
	182	X	X	X	X	X	X	X	n.s.	n.s.	15.97	17.95	-	X	-	-	-	n.d.	n.d.	x	-	x
	226	X	X	X	X	X	X	X	n.s.	n.s.	17.88	16.76	-	X	-	X	X	2.3 ± 0.3[a]	2.3 ± 0.9[a]	-	-	-
	367	X	X	X	X	X	X	X	n.s.	n.s.	0.38	0.53	-	-	-	X	X	4.4 ± 0.7[a]	4.9 ± 1.0[a]	-	-	-
	463	X	-	-	-	-	-	X	n.s.	n.s.	28.93	21.93	-	-	-	n.e.	n.e.	n.e.	n.e.	n.e.	n.e.	n.e.
	554	X	X	X	X	X	X	X	n.s.	n.s.	35.93	30.31	-	-	-	X	X	4.7 ± 0.6[a]	7.2 ± 1.5[a]	-	-	-
	564	X	X	X	X	X	X	X	n.s.	n.s.	0.44	0.44	-	-	-	X	X	3.3 ± 0.6[b]	3.8 ± 0.9[a]	-	-	-
	710	X	X	X	X	X	X	X	n.s.	n.s.	0.21	0.21	-	-	-	-	-	n.d.	n.d.	x	-	x
	727	X	X	X	X	X	X	X	n.s.	n.s.	n.e.	n.e.	-	-	-	X	X	17.1 ± 0.9[a]	15.4 ± 0.1[a]	-	-	-
NDUFS2	9	X	-	-	-	-	-	-	n.s.	n.s.	n.e.	n.e.	X	-	-	n.e.	n.e.	n.e.	n.e.	n.e.	n.e.	n.e.
	109	X	X	X	X	X	X	X	n.s.	n.s.	1.23	1.96	-	-	-	-	-	n.d.	n.d.	-	-	-
	146	X	X	X	X	X	X	X	n.s.	n.s.	1.77	1.74	-	-	-	X	X	26.7 ± 7.0[a]	44.5 ± 18[a]	-	-	-
	326	X	X	X	X	X	X	X	n.s.	n.s.	0.00	0.19	-	-	-	-	-	n.d.	n.d.	x	-	x
	332	X	X	X	X	X	X	X	n.s.	n.s.	0.00	1.36	-	-	-	-	-	n.d.	n.d.	x	-	x
	347	X	X	X	X	X	X	X	n.s.	n.s.	0.34	3.28	-	-	-	X	X	1.7 ± 0.1[a]	1.9 ± 0.2[a]	-	-	-
	422	X	X	X	X	X	X	X	n.s.	n.s.	0.00	0.57	-	-	-	-	-	n.d.	n.d.	x	-	x
NDUFS3	10	X	-	-	-	-	X	-	n.e.	n.s.	n.e.	n.e.	X	-	-	n.e.	n.e.	n.e.	n.e.	n.e.	n.e.	n.e.
	79	X	X	X	X	X	X	X	X	n.s.	18.73	14.51	-	-	-	-	-	n.d.	n.d.	-	X	-
	86	X	X	X	-	X	X	-	-	n.s.	1.40	2.86	-	-	-	-	-	n.d.	n.d.			-
NDUFS4	28	X	-	-	-	-	X	-	n.s.	n.s.	n.e.	n.e.	X	-	-	n.e.	n.e.	n.e.	n.e.	n.e.	n.e.	n.e.
NDUFS5	33	X	X	X	X	X	X	X	n.s.	n.s.	0.41	0.00	-	-	33-66	-	-	n.d.	n.d.	x	-	x
	43	X	X	X	X	X	X	X	n.s.	n.s.	3.67	1.87	-	-	43-56	X	-	n.d.	0 ± 0[b]	-	-	-
	56	X	X	X	X	X	X	X	n.s.	n.s.	0.00	0.00	-	-		-	-	n.d.	n.d.	x	-	x
	66	X	X	X	X	X	X	X	n.s.	n.s.	2.79	3.20	-	-	33-66	-	-	n.d.	n.d.	x	-	x
NDUFS6	79	X	X	X	X	n.s.	X	X	n.s.	n.s.	1.31	3.22	-	-	-	X	X	1.4 ± 0.1[a]	1.6 ± 0.4[a]	-	-	-
	104	X	X	X	X	n.s.	X	-	n.s.	n.s.	0.00	0.00	-	-	-	X	X	0 ± 0[a]	1.9 ± 1.1[a]	-	X	-
	107	X	X	X	X	n.s.	X	-	n.s.	n.s.	2.90	4.28	-	-	-	X	X					-
NDUFS7	99	X	X	X	X	X	X	X	n.s.	n.s.	16.20	17.11	-	X	-	-	-	n.d.	n.d.	-	X	-
	100	X	X	X	X	X	X	X	n.s.	n.s.	7.43	5.86	-	X	-	-	-	n.d.	n.d.			-
	164	X	X	X	X	X	X	X	n.s.	n.s.	23.81	21.70	-	X	-	X	-	0.0[c]	n.d.	-	-	-
	181	X	X	X	X	X	X	X	n.s.	n.s.	0.00	2.17	-	-	-	-	-	n.d.	n.d.	x	-	x
	194	X	X	X	X	X	X	X	n.s.	n.s.	7.22	6.37	-	X	-	X	X	1.0 ± 1.0[a]	8.6 ± 6.5[a]	-	-	-
NDUFS8	113	X	X	X	X	X	X	X	n.s.	n.s.	11.42	11.94	-	X	-	-	-	n.d.	n.d.	X	X	x
	116	X	X	X	X	X	X	X	n.s.	n.s.	10.57	9.96	-	X	-	-	-	n.d.	n.d.			x
	119	X	X	X	X	X	X	X	n.s.	n.s.	13.44	12.00	-	X	-	X	X	2.0 ± 0.2[a]	3.4 ± 1.0[a]	-	X	-
	123	X	X	X	X	X	X	X	n.s.	n.s.	14.36	14.19	-	X	-	X	X			-		-
	152	X	X	X	X	X	X	X	n.s.	n.s.	7.87	8.30	-	X	-	-	-	n.d.	n.d.	-	X	x
	155	X	X	X	X	X	X	X	n.s.	n.s.	11.56	10.32	-	X	-	-	-	n.d.	n.d.			x
	158	X	X	X	X	-	X	X	n.s.	n.s.	10.22	12.73	-	X	-	-	-	n.d.	n.d.			x
	162	X	X	X	X	-	X	X	n.s.	n.s.	13.13	10.64	-	X	-	-	-	n.d.	n.d.			x
NDUFV1	125	X	X	X	X	X	X	n.s.	n.s.	n.s.	3.61	5.75	-	-	-	X	X	2.0 ± 0.3[a]	3.3 ± 0.4[a]	-	-	-
	142	X	X	X	X	X	X	n.s.	n.s.	n.s.	0.00	0.00	-	-	-	X	X	2.5 ± 0.7[a]	6.8 ± 4.3[a]	-	-	-
	187	X	X	X	X	X	X	n.s.	n.s.	n.s.	15.76	4.29	-	-	-	X	X	6.0 ± 1.4[a]	12.8 ± 2.3[a]	-	-	-
	206	X	X	X	X	X	X	n.s.	n.s.	n.s.	0.00	0.00	-	-	-	X	X	2.2 ± 0.3[a]	1.5 ± 0.5[a]	-	-	-
	238	X	X	X	X	X	X	n.s.	n.s.	n.s.	0.21	0.00	-	-	-	X	X	0.4 ± 0.1[a]	0.6 ± 0.1[a]	-	X	-
	255	X	X	X	X	X	X	n.s.	n.s.	n.s.	0.00	0.00	-	-	-	X	X			-		-
	286	X	X	X	X	X	X	n.s.	n.s.	n.s.	0.00	0.00	-	-	-	X	X	1.1 ± 0.1[a]	1.4 ± 0.0[a]	-	-	-
	332	X	X	X	X	X	X	n.s.	n.s.	n.s.	1.62	3.70	-	-	-	-	-	n.d.	n.d.	-	-	-
	379	X	X	X	X	X	X	n.s.	n.s.	n.s.	10.97	10.30	-	X	-	-	-	n.d.	n.d.	-	X	-
	382	X	X	X	X	X	X	n.s.	n.s.	n.s.	11.81	10.62	-	X	-	-	-	n.d.	n.d.			-
	385	X	X	X	X	X	X	n.s.	n.s.	n.s.	13.38	12.89	-	X	-	-	-	n.d.	n.d.			-
	425	X	X	X	X	X	X	n.s.	n.s.	n.s.	15.37	14.40	-	X	-	X	X	2.2 ± 1.5[a]	2.4 ± 0.4[a]	-	-	-
NDUFV2	134	X	X	X	X	n.s.	X	X	n.s.	n.s.	1.75	0.79	-	X	-	-	-	n.d.	n.d.	-	X	-
	139	X	X	X	X	n.s.	X	X	n.s.	n.s.	2.46	1.87	-	X	-	-	-	n.d.	n.d.			-
	175	X	X	X	X	n.s.	X	X	n.s.	n.s.	0.38	0.16	-	X	-	-	-	n.d.	n.d.	-	X	-
	179	X	X	X	X	n.s.	X	X	n.s.	n.s.	1.92	1.34	-	X	-	-	-	n.d.	n.d.			-
	223	X	-	-	-	n.s.	X	-	n.s.	n.s.	0.19	0.77	-	-	-	n.e.	n.e.	n.e.	n.e.	n.e.	n.e.	n.e.
	224	X	X	X	X	n.s.	X	X	n.s.	n.s.	4.81	2.44	-	-	-	X	X	1.7 ± 0.5[a]	8.8 ± 3.8[a]	-	-	-
NDUFV3																						

Legend:

n.s. = no sequence
n.e. = not existing
n.d. = no data
X yes/positive
- no/negative

Exposure γ-sulfur atom ($Å^2$):
No exposure
Exposure <5 $Å^2$
Exposure >5 $Å^2$
5 $Å^2$ cutoff for solvent exposure
(James et al.,2018, Cell Reports 24, 1445-1455)

Remarks:
a N=3 ± S.E.M.
b N=2 ± range
c N=1
d Peptide with missed cleavage

Sup Table 2. Quantitation of the cysteine survey by MS - related to Sup Table 1

138 Cysteines found in mouse
116 (84.1 %) of these cysteines are conserved in bovine
114* (82.6/98.3 %) of these residues are found in the mature bovine enzyme

	Total (114*)	Cys detected	Cys quantified	Cys unquantified	Cys undetected	FeS cluster	Disulfide	Undetectable	Detectable
Total	100 % (114)	45.6 % (52)	37.7 % (43)	62.3 % (71)	54.4 % (62)	27.2 % (31)	22.8 % (26)	28.1 % (32)	72.9 % (82)
Cys detected	45.6 % (52)	-	-	12.7 % (9)	-	38.7 % (12)	30.8 % (8)	-	63.4 % (52)
Cys quantified	37.7 % (43)	82.7 % (43)	-	-	-	22.6 % (7)	23.1 % (6)	-	52.4 % (43)
Cys unquantified	62.3 % (71)	17.3 % (9)	-	-	-	77.4 % (24)	77.0 % (20)	-	47.6 % (39)
Cys undetected	54.4 % (62)	-	-	87.3 % (62)	-	61.3 % (19)	69.2 % (18)	100.0 % (32)	-
FeS cluster	27.2 % (31)	23.1 % (12)	16.3 % (7)	33.8 % (24)	30.7 % (19)	-	-	31.3 % (10)	25.6 % (21)
Disulfide	22.8 % (26)	15.4 % (8)	11.6 % (6)	28.2 % (20)	29.0 % (18)	-	-	31.3 % (10)	19.5 % (16)
Undetectable	28.1 % (32)	-	-	45.1 % (32)	51.6 % (32)	32.3 % (10)	38.5 % (10)	-	-
Detectable	72.9 % (82)	100 % (52)	100 % (43)	54.9 % (39)	48.3 % (30)	67.7 % (21)	61.5 % (16)	-	-

* NDUFAB1 is present in two copies within complex I but the cysteines are indistinguishable by mass spectrometry and therefore counted as a unique cysteine
